# A reliable transcriptomic risk-score applicable to formalin-fixed paraffin-embedded biopsies improves outcome prediction in localized prostate cancer

**DOI:** 10.1186/s10020-024-00789-9

**Published:** 2024-02-01

**Authors:** Michael Rade, Markus Kreuz, Angelika Borkowetz, Ulrich Sommer, Conny Blumert, Susanne Füssel, Catharina Bertram, Dennis Löffler, Dominik J. Otto, Livia A. Wöller, Carolin Schimmelpfennig, Ulrike Köhl, Ann-Cathrin Gottschling, Pia Hönscheid, Gustavo B. Baretton, Manfred Wirth, Christian Thomas, Friedemann Horn, Kristin Reiche

**Affiliations:** 1 Department of Diagnostics, Fraunhofer Institute for Cell Therapy and Immunology, Leipzig, Germany; 2 Department of Urology, Faculty of Medicine, University Hospital, Technische Universität Dresden, Dresden, Germany; 3 Institute of Pathology, Faculty of Medicine, University Hospital, Technische Universität Dresden, Dresden, Germany; 4 Basic Science Division, Computational Biology Program, Fred Hutchinson Cancer Center, Seattle, WA USA; 5 Institute of Clinical Immunology, University of Leipzig, Leipzig, Germany; 6 Center for Scalable Data Analytics and Artificial Intelligence (ScaDS.AI), University of Leipzig, 04105 Leipzig, Germany

**Keywords:** Prognostic biomarker, Molecular diagnostic testing, Molecular pathology, Personalized medicine, Prostate cancer, Transcriptome

## Abstract

**Background:**

Clinical manifestation of prostate cancer (PCa) is highly variable. Aggressive tumors require radical treatment while clinically non-significant ones may be suitable for active surveillance. We previously developed the prognostic ProstaTrend RNA signature based on transcriptome‐wide microarray and RNA-sequencing (RNA-Seq) analyses, primarily of prostatectomy specimens. An RNA-Seq study of formalin-fixed paraffin-embedded (FFPE) tumor biopsies has now allowed us to use this test as a basis for the development of a novel test that is applicable to FFPE biopsies as a tool for early routine PCa diagnostics.

**Methods:**

All patients of the FFPE biopsy cohort were treated by radical prostatectomy and median follow-up for biochemical recurrence (BCR) was 9 years. Based on the transcriptome data of 176 FFPE biopsies, we filtered ProstaTrend for genes susceptible to FFPE-associated degradation via regression analysis. ProstaTrend was additionally restricted to genes with concordant prognostic effects in the RNA-Seq TCGA prostate adenocarcinoma (PRAD) cohort to ensure robust and broad applicability. The prognostic relevance of the refined Transcriptomic Risk Score (TRS) was analyzed by Kaplan–Meier curves and Cox-regression models in our FFPE-biopsy cohort and 9 other public datasets from PCa patients with BCR as primary endpoint. In addition, we developed a prostate single-cell atlas of 41 PCa patients from 5 publicly available studies to analyze gene expression of ProstaTrend genes in different cell compartments.

**Results:**

Validation of the TRS using the original ProstaTrend signature in the cohort of FFPE biopsies revealed a relevant impact of FFPE-associated degradation on gene expression and consequently no significant association with prognosis (Cox-regression, p-value > 0.05) in FFPE tissue. However, the TRS based on the new version of the ProstaTrend-ffpe signature, which included 204 genes (of originally 1396 genes), was significantly associated with BCR in the FFPE biopsy cohort (Cox-regression p-value < 0.001) and retained prognostic relevance when adjusted for Gleason Grade Groups. We confirmed a significant association with BCR in 9 independent cohorts including 1109 patients. Comparison of the prognostic performance of the TRS with 17 other prognostically relevant PCa panels revealed that ProstaTrend-ffpe was among the best-ranked panels. We generated a PCa cell atlas to associate ProstaTrend genes with cell lineages or cell types. Tumor-specific luminal cells have a significantly higher TRS than normal luminal cells in all analyzed datasets. In addition, TRS of epithelial and luminal cells was correlated with increased Gleason score in 3 studies.

**Conclusions:**

We developed a prognostic gene-expression signature for PCa that can be applied to FFPE biopsies and may be suitable to support clinical decision-making.

**Supplementary Information:**

The online version contains supplementary material available at 10.1186/s10020-024-00789-9.

## Background

The clinical manifestation of prostate cancer (PCa) is highly variable. Aggressive types of PCa require radical treatment such as radical prostatectomy (RPx) or radiotherapy, while low-risk PCa may be suitable for active surveillance or organ-preserving focal therapies. In clinically localized PCa, risk stratification is based on prostate-specific antigen (PSA), Gleason score (GS) and clinical or imaging T stage (Mottet et al. 2021). Multi-parametric magnetic resonance imaging (mpMRI) is also preferred for local tumor staging, which may thereby serve as a basis for therapy (Park et al. 2014; Abrams-Pompe et al. 2021).These clinicopathologic variables have been used to develop nomograms such as MSKCC (Cagiannos et al. 2003), Briganti (Briganti et al. 2012) and Partin (Makarov et al. 2007) tables, which are used to predict lymph node invasion. Other methods such as Stephenson nomogram or the Cancer of the Prostate Risk Assessment Postsurgical (CAPRA-S) score are used in the post-surgical setting to predict the likelihood of recurrence after RPx (Stephenson et al. 2005; Cooperberg et al. 2011). In addition, CT scan and mpMRI can evaluate the locoregional extension of the disease by detecting extracapsular extension and seminal vesicle invasion (Somford et al. 2013).

In localized PCa, these risk assessment tools are critical for the management of patients who can be treated with a wide range of strategies, including RPx, radiation, focal therapy or active surveillance (Mottet et al. 2021). Although essential in daily clinical practice, stratification by clinical risk categories or available tests lacks sufficient precision. For example, nomograms based on clinical parameters do not allow the precise distinction of an non-aggressive instead of an aggressive disease (Wang et al. 2014), which may lead to substantial overtreatment of patients.

Prognostic biomarkers can help to distinguish non-significant from aggressive cancers and could improve clinical decision-making. For example, such markers should be able to discriminate between aggressive diseases to enable therapeutic/surgical interventions that would be unnecessary for non-aggressive cancers. In recent years, several blood, urine, and tissue-based biomarkers for PCa have been introduced. Commercially available tissue-based mRNA gene expression classifiers include Decipher (Erho et al. 2013), Prolaris (Cuzick et al. 2011), and Oncotype Dx (Cullen et al. 2015).

Combining prognostic biomarkers that predict the likelihood of an adverse outcome with already available clinicopathological variables might aid in the decision-making process and result in a more individualized course of treatment for each patient (Fine et al. 2019). For example, the mRNA-based Prolaris signature assesses the expression of 31 genes related to the cell cycle and is represented as a cell cycle progression (CCP) score. In conjunction with the CAPRA score (referred to as the CCR score), the concordance index (c-index) for predicting 10-year PCa-specific mortality in 585 men with localized PCa was 0.74 for CAPRA and improved to 0.78 for CCR (Cuzick et al. 2015). This combined CCR score added significant risk stratification to what is available from clinicopathological variables alone.

We previously developed the transcriptome-based prognostic ProstaTrend signature, the only test besides the commercially available Prolaris (Cuzick et al. 2011, 2015) classifier suitable for long-term prognosis because it predicts the time to death of disease (DoD) after RPx (Kreuz et al. 2020). ProstaTrend comprises 1396 genes and predicts DoD and biochemical recurrence (BCR) in cohorts of PCa patients treated with RPx. The prognostic impact persisted after adjusting for the clinical risk factors Gleason Grade Group (GGG), resection status and pathological stage (pT).

To improve patient stratification and to assess risk of progression more accurately for clinical decision-making, prognostic performance of signatures needs to be verified for application in clinical routine material. The development of the ProstaTrend signature was mainly based on tissue specimens from RPx. Only 16 (7%) samples were obtained from prostate biopsies in our previous study (Kreuz et al. 2020). However, a prognostic signature is needed that can also be applied to biopsy cohorts eligible for active surveillance or focal therapy to assess the potential of the score to select patients for conservative therapy, focal therapy or radical treatment such as RPx or radiotherapy.

In addition, the majority of our training cohort comprised fresh-frozen (FF) specimens of optimal quality n = 204 (88%) and was restricted to samples with > 50% tumor cell content. For formalin-fixed paraffin-embedded (FFPE) specimens, the fixation process usually causes degradation and fragmentation, which limits gene detection and introduces sequencing artifacts (Groelz et al. 2013; Adiconis et al. 2013). A prognostic signature verified using FFPE specimens would have significant added value for both prospective and retrospective archive-based studies since FFPE preservation is common in clinical routine.

Furthermore, the ProstaTrend signature was developed from tumor bulk transcriptomes. Since the bulk transcriptome consists of multiple cell identities and biological programs, it cannot explain PCa heterogeneity at the single-cell level.

To transfer and validate the genes included in the ProstaTrend signature for the application in clinical routine at the time point of PCa diagnosis and before treatment, we used a cohort of 176 specimens obtained from FFPE-conserved prostate biopsies with varying tumor cell content and long clinical follow-up by transcriptome-wide total RNA-Seq. We developed a PCa single-cell atlas to analyze ProstaTrend genes in different cell compartments. Lastly, we validated the association of the transcriptomic risk score (TRS) with BCR in 9 publicly available cohorts and compared the prognostic performance of ProstaTrend with 17 other prognostically relevant PCa gene panels.

## Methods

### Data sources

#### Internal cohort (FFPE_Bx cohort)

We assessed tissue specimens with long term follow-up data (median follow-up 9 years) from FFPE biopsy specimens by transcriptome-wide total RNA-Seq. We refer to this cohort as the FFPE_Bx cohort. This study was designed, conducted and reported in accordance with the Reporting Recommendations for tumor marker prognostic studies (REMARK) guidelines (McShane et al. 2005).

From a cohort of biopsy specimens consisting of 543 PCa patients who underwent RPx between 2007 and 2013 at the Department of Urology of the University Hospital Dresden (Germany), we included 192 specimens in our study. We excluded 7 specimens after evaluation of RNA yield (RNA yield < 50 ng). None of the patients did receive neoadjuvant therapy prior to surgery. The Internal Review Board at the Technische Universität Dresden (EK194092004, EK59032007) approved the study, and all patients gave written informed consent. Clinicopathological parameters were obtained by routine histopathological examination of the surgical specimens. Serum levels of the prostate-specific antigen (PSA) were determined pre-biopsy and pre-RPx. The primary endpoint of this cohort was BCR and was defined as a PSA level ≥ 0.2 ng/mL after RPx. Information on the course of the disease, survival of the patients and the cause of death were obtained from treating urologists or the general practitioners or from records of the regional tumor registry. RNA isolation, quantification, cDNA library production and RNA-Seq of FFPE specimens are described in Additional file 1. See Additional file 1: Tables S1, S2 and Additional file 2: Table S19 for technical and clinicopathological characteristics.

#### Validation cohorts

To find appropriate transcriptome datasets of PCa cohorts for our analyses, we relied on the literature research of the study by Li et al. (2021). Furthermore, we performed a literature screening on https://pubmed.ncbi.nlm.nih.gov/ using the following keywords: “prostate AND cancer AND (bcr OR biochemical recurrence) AND (transcriptomics OR transcriptome OR rna-seq OR rna sequencing OR microarray)” (2022/06). Datasets that met the following inclusion criteria for PCa datasets were incorporated in this study: (1) The patients in the cohorts were required to have a complete record of time to BCR or time to last follow-up. (2) Cohorts were rejected if BCR has not occurred with at least 10 events after quality filtering of the samples (see below). (3) Tumors had to be derived from the primary site. (4) Raw or pre-processed gene expression data must be available (microarray or RNA-Seq). In total, we analyzed 9 publicly available cohorts (Li et al. 2020; Fraser et al. 2017; Luca et al. 2018; Long et al. 2014; Gerhauser et al. 2018; Jain et al. 2018; Ross-Adams et al. 2015; Taylor et al. 2010) to evaluate the prognostic performance of the ProstaTrend and ProstaTrend-ffpe signatures for predicting time to BCR. Additional file 1: Table S1 provides an overview of the technical parameter of the datasets analyzed in this study. Samples were quality filtered as described in the original publication of the datasets [details are outlined in Additional file 1: Table S1 (see column “Notes”)]. Additional file 1: Table S3 contains the clinicopathological parameters of patients fulfilling the above conditions.

In the case of Affymetrix array datasets, raw.CEL files including CPC_GENE_2017_Fraser (GSE84042), MSKCC_2010_Taylor (GSE21034) and CancerMap_2017_Luca (GSE94767) were downloaded from NBCI’s Gene Expression Omnibus (GEO) (Edgar et al. 2002) using the R package GEOquery v2.58.0 (Davis and Meltzer 2007). For array datasets from other platforms, the processed array data of cohorts CamCap_2016_Ross_Adams (GSE70768), Stockholm_2016_Ross_Adams (GSE70769) and Belfast_2018_Jain (GSE116918) were obtained using GEOquery. Processed RNA-Seq data (FPKM normalized) from the cohort DKFZ_2018_Gerhauser were downloaded from cBioPortal (Gao et al. 2013). The RPKM normalized counts from the Chinese Prostate Cancer Genome and Epigenome Atlas (CPGEA_2020_Li) were downloaded from http://www.cpgea.com. Raw sequencing data in FASTQ format from the RNA-Seq project Atlanta_2014_Long (GSE54460) were obtained using the prefetch and fastq-dump commands implemented in the SRA Toolkit v2.9.2 (https://github.com/ncbi/sratoolkit).

#### TCGA PRAD cohort

We used RNA-Seq data of the TCGA prostate adenocarcinoma (PRAD) cohort from the Cancer Genome Atlas (Abeshouse et al. 2015) for external gene filtering of ProstaTrend. See Additional file 1: Tables S1, S2 for technical and clinicopathological characteristics of TCGA PRAD. The Data source is described in Kreuz et al. (2020).

### Pre-processing

#### Internal FFPE_Bx cohort

To facilitate the multi-step analysis of the RNA-Seq data, we applied the workflow-manager uap v1.0.1 (Kämpf et al. 2019). A detailed description of all processing steps from FASTQ files to gene quantification can be found in Additional file 1. Gene counts were adjusted for library size and normalized with the variance-stabilizing transformation (vst) as implemented in DESeq2 v1.30.1 (Love et al. 2014). The vst method was run with the option “blind = TRUE” to compare samples in an unbiased manner. We used the R package geneFilter (Gentleman et al. 2018) to remove low expressed genes using the function varFilter() with parameters var.func = IQR and var.cutoff = 0.25. Genes with an interquartile range (IQR) smaller than the 25th percentile of all IQR in the vst normalized expression data were filtered out.

Overall, 6 samples were excluded after quality filtering (see Additional file 1 for details). In addition, 3 samples were excluded because the time to BCR was not recorded.

#### Validation cohorts and TCGA PRAD

A detailed description of all processing steps for the validation cohorts is provided in Additional file 1. The pre-processing steps of the TCGA PRAD cohort are described in Kreuz et al. (2020).

Normalized expression values (within and between sample normalization) from all cohorts were transformed to log or vst-space (Additional file 1: Table S1). Each cohort was individually standardized by calculating gene-wise z-scores. The same gene expression filter as for FFPE_Bx was used for all cohorts.

### Statistical analysis

#### Calculation of transcriptomic risk score (TRS) per sample

To validate the ProstaTrend signature, we applied the TRS as published previously (Kreuz et al. 2020). In short, the TRS of a sample *k* is the median over the weighted standardized expression values of all genes included in ProstaTrend. The weight for each significant gene was the estimated combined log hazard ratio (logHR) from a univariate meta-analysis approach of the training cohorts. The logHR values were estimated using Cox proportional hazards models for each training cohort.

#### Impact of confounding factors and subsequent gene filtering to define the set of genes for ProstaTrend-ffpe

To assess the impact of tumor cell content and the influence of RNA degradation that is associated with FFPE conservation over time, we applied linear regression models. For each gene, we fitted two linear regression models, one to estimate the impact of tumor cell content on gene expression and another to estimate the association between the age of the FFPE specimens, i.e., the time of conservation, and gene expression. For genes associated with specimen age, we assumed that the prognostic information of these genes might by distorted by underlying differences in specimen age. Therefore, we filtered out ProstaTrend genes where linear regression indicated an association (linear regression p-value < 0.1). We did not filter out genes associated with tumor cell content of the samples or sequencing depth, as we did not find improved prognostic accuracy (see Additional file 1 for details). In addition, we restricted ProstaTrend to genes having a consistent prognostic effect between the training cohorts and TCGA PRAD (univariate Cox-regression with p-value < 0.1 in TCGA PRAD and consistent logHR).

In the study by Kreuz et al. (2020), we identified 20 putative new (unknown intergenic) prognostic genes out of a total of 1396. We did not include these genes in the downstream analyses because we could not identify them in arrays and RNA-Seq validation cohorts (due to unavailability of raw data for some cohorts). The novel ProstaTrend-ffpe gene signature contains 204 genes, of which one gene is putative novel.

#### Survival analyses

We performed survival analyses using the R survival package v3.1-12 (Therneau 2015) and rms v6.2-0. Kaplan–Meier plots for patients with TRS > 0 (increased risk) compared to patients with TRS ≤ 0 (reduced risk) were generated using the ggsurvplot function from the survminer v0.4.9 R package. Primary survival endpoint of the cohort was time to BCR. Log-rank tests were performed to compare probabilities of BCR-free survival between these two groups using the survdiff function implemented in the survival package. Cox proportional hazards models were performed on continuous TRS in a univariate regression model using the coxph function from the survival package. Since Gleason score is a major prognostic factor in PCa, we applied multivariable Cox-regression to assess the performance of TRS with adjustment for Gleason Grade Groups (GGG). Because a large proportion of patients in the FFPE_Bx cohort had a Gleason score of 7, we used GGG (Epstein et al. 2014) for adjustment of the regression model to achieve a more detailed breakdown of this group.

To compare the performance of the TRS with “non-prognostic” random gene sets in the 9 validation cohorts (Fig. 4C), we formed for each validation cohort 1000 random gene sets for ProstaTrend and ProstaTrend-ffpe [hereinafter also referred to as ProstaTrend(-ffpe)], respectively. The random gene sets were drawn from all genes used in the meta-analysis of the ProstaTrend training cohort that were not part of the final ProstaTrend gene set [i.e., that had an false discovery rate (FDR) > 0.05 (Kreuz et al. 2020)]. Random gene sets had the size of the overlap for the corresponding gene set and respective cohort. In addition, the random gene sets had to have passed the gene expression filter (see “Pre-processing” section). For each random gene set and validation cohort, we applied the TRS to each sample and performed a Cox-regression analysis for the TRS on a continuous scale. The weights for the included genes in random gene sets were the estimated logHRs from the ProstaTrend meta-analysis of the training cohorts for the respective genes.

#### Comparison of ProstaTrend(-ffpe) to other prognostic PCa signatures

To compare performance of the TRS with other prognostic gene expression signatures, we relied on gene signatures as reported in the study by Li et al. (2022). We excluded signatures that were not designed for gene expression in human PCa tumor tissue or that were not specific for PCa. In addition, signatures that differentiated tumors into more than two groups were excluded because a direct association of individual genes with prognosis has not been described in the original publication for these cases. Lastly, signatures were excluded for which the direction of the prognostic effect of the included genes was unknown. For the signature of Erho et al. (2013) the direction of the prognostic effects was derived from Creed et al. (2020), and intronic or non-coding markers were excluded due to missing documentation of the genomic regions for these markers. An overview of all 17 included and excluded prognostic signatures is provided in Additional file 1: Table S6.

Prognostic signatures were applied to cohort-wise standardized expression values. Since individual weights of the genes involved were not known for all signatures or these were optimized for different technical platforms, we simplified the calculation of the risk scores by taking only the reported directions of the gene-wise effects into account. To ensure comparability, we restricted the gene weights for ProstaTrend(-ffpe) in the same manner. Thus, the risk score for each signature was calculated by the median expression of signature genes associated with high risk (logHR > 0) minus the median expression of signature genes associated with reduced risk (logHR < 0). The binary classification of genes according to risk was, in our opinion, the fairest approach.

#### Characterizing the performance of ProstaTrend(-ffpe) genes by a meta-analysis

The following data sets were used for a meta-analysis to illustrate the performance of individual ProstaTrend genes: subsets of the training cohorts used to develop the ProstaTrend signature (Kreuz et al. 2020), the FFPE_Bx cohort, the TCGA PRAD cohort, and the 9 validation cohorts. The training datasets included the cohorts FF_array_RP and FF_seq_RP with DoD as the endpoint. The FF_array_RP cohort included 164 patients with 25 events and the FF_seq_RP cohort included 40 patients with 12 events. For clinicopathological parameters, see Kreuz et al. (2020). We did not include the FFPE_Seq_RP and FFPE_Seq_Bx training cohorts, which we initially used in Kreuz et al. to develop the ProstaTrend signature in the meta-analysis. This is justified by the small number of patients and events (< 10 events per cohort) and the resulting large standard deviation for the estimated effect size of individual genes. This results in an unreliable estimation of prognostic relevance in these cohorts and, ultimately a low impact on the estimate of the combined effect size. This filtering step is consistent with the inclusion criteria for the validation cohorts described above.

We included all ProstaTrend genes annotated with an Ensembl ID and present in at least 2 cohorts in the meta-analysis. For each gene, a univariate Cox-regression model was applied to standardized expression values in log-space for each cohort individually. For the ProstaTrend training cohorts, it should be noted that due to a matched study design, patients from FF_seq_RP were included in FF_array_RP. Therefore, we used a weighted Cox-regression with weights 1/N, where N is the number of samples for the specific patient included across the two cohorts. Similarly, 40 patients are included in both the CancerMap_2017_Luca and CamCap_2016_Ross_Adams cohorts. However, since these patients are not identifiable, no adjustment could be made for these duplicates. Given the small number of duplicate patients and the large number of cases (n = 1821), the effects of higher weights for matched samples are negligible. Using the logHR values and the respective standard errors calculated from the coxph function, we estimated a combined effect size for each gene. We used a random effects model because we did not assume that there is one true effect size, which is shared by all the included cohorts, but rather a range of true effect sizes with additional sources of variation, such as different platforms (RNA-Seq and microarray), clinical or demographic variables, etc. The model was fitted with the restricted maximum-likelihood estimator using the R package meta v4.19.0 (Schwarzer 2007). We declared that a prognostic gene from the ProstaTrend(-ffpe) signature had a significant combined effect size when the FDR adjusted p-value (Benjamini–Hochberg method) was < 0.05. For Fig. 5C, we calculated the log odds ratios derived from logistic regression model predicting GS > 7 vs. ≤ 7. The combined effect size for the log odds ratios was calculated as described above.

### PCa cell atlas

Raw read counts from scRNA-Seq data of 5 studies were downloaded from NCBI GEO. This included the studies Chen et al. (2021) (GSE141445), Dong et al. (2020) (GSE137829), Ma et al. (2020) (GSE157703) and Song et al. (2022) (GSE176031). Read counts from the study Tuong et al. (2021) were obtained from https://www.prostatecellatlas.org. We excluded one sample from the study by Chen et al. because it was a biopsy of a lymph node metastasis that could not be directly compared with primary tumor. Pre-processing, integration, clustering, quality control, cell cluster annotation, estimation of copy number variations (CNVs) and differential gene expression analysis (DGEA) are described in Additional file 1.

Using the study-wise standardized, normalized expression values of the scRNA-Seq datasets, we applied for each cell a simplified ProstaTrend TRS, which was the mean of all genes at increased risk minus the mean of genes at reduced risk. The simplification of the score was necessary due to the low expression levels and high drop-out rate of individual genes in the scRNA-Seq data, which makes the weights inapplicable.

### Spatial transcriptomics data analysis

To apply the TRS (using the same strategy as for the PCa cell atlas) to spots in spatial transcriptomics data, we re-analyzed biopsies from human benign prostate tissue and from a human PCa stage III with GS7a. The biopsies were FFPE preserved and processed using the Visium spatial gene expression for FFPE workflow. The datasets are publicly available at 10× Genomics (https://www.10xgenomics.com/resources/datasets). Pathological annotations were done at an overview level and were performed by Agoko NV, Belgium. Pre-processing, integration and clustering are described in Additional file 1.

We calculated diagnostic odds ratios (DOR) for all significantly differentially expressed (DE) genes from the DGEA of the PCa cell atlas. DOR values are based on a metric that evaluates the specificity of cell type markers (Adams et al. 2020) (see Additional file 1 for details). To define gene sets of cell type specific markers, we filtered for significantly differentially expressed genes with a DOR > 2 and a log2 fold change > 1. For tumor-specific luminal (T-luminal) cell markers, we used a DOR > 1. Each spot was scored for enrichment of cell types and T-luminal markers using the function AddModuleScore implemented in Seurat (Hao et al. 2021). Since the spots are composed from a small number of cells but still reflect cell populations, we did not assign single spots to a specific cell type.

## Results

**Fig. 1:**
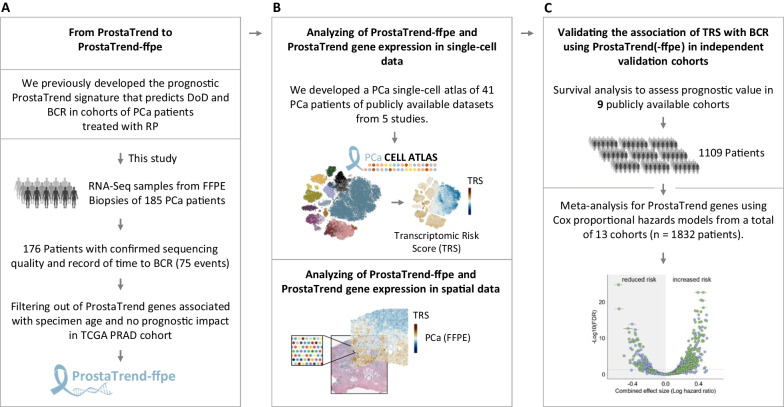
Analysis workflow. **A** Overview of the included tissue samples we used to develop the ProstaTrend-ffpe signature. Shown are the number of patients included in the study and reasons for exclusion. For 185 patients, we performed a strand-specific transcriptome-wide sequencing from FFPE biopsy tissue. A total of six samples did not meet the quality criteria for RNA-Seq data. In addition, we excluded three samples due to missing clinical follow-up data. The final cohort included 176 patients, for 75 of whom BCR was observed within the follow-up time. *FFPE* formalin-fixed paraffin-embedded, *PCa* prostate cancer, *DoD* death of disease, *BCR* biochemical recurrence. **B** For the development of the PCa single-cell atlas, we used scRNA-Seq data of PCa patients from 5 publicly available studies (Chen et al. 2021; Dong et al. 2020; Ma et al. 2020; Song et al. 2022; Tuong et al. 2021). Spatial transcriptomics data of a human PCa biopsy (GS = 3 + 4) were downloaded from the 10× Genomics database. **C** The prognostic value of the Transcriptomic Risk Scores (TRS) using ProstaTrend(-ffpe) was evaluated by survival analyses in 9 publicly available cohorts (Li et al. 2020; Fraser et al. 2017; Luca et al. 2018; Long et al. 2014; Gerhauser et al. 2018; Jain et al. 2018; Ross-Adams et al. 2015; Taylor et al. 2010) and a meta-analysis with a total of 13 cohorts

For validation of the prognostic ProstaTrend signature, we followed a three-step approach, as shown in Fig. 1. First, we validated the genes included in the ProstaTrend signature (Kreuz et al. 2020) in the cohort of FFPE prostate biopsies (n = 185 biopsy specimens), and formed a new subset of genes suitable for application in FFPE biopsies. Second, we developed a single-cell transcriptome atlas from cells of 41 PCa patients using publicly available scRNA-Seq data and analyzed gene expression of ProstaTrend genes in different cell compartments. Third, we confirmed the prognostic value of the ProstaTrend and ProstaTrend(-ffpe) signature in 9 publicly available cohorts with a total of 1109 patients.

### Development of the new ProstaTrend-ffpe signature utilizing the ProstaTrend gene set and an RNA-Seq dataset from FFPE biopsies

We performed RNA-Seq of FFPE biopsy tissue from 185 PCa patients (referred to as FFPE_Bx cohort). 179 samples passed the quality filter, three samples were excluded because time to BCR was not recorded. In total, we analyzed transcriptome data from 176 patients, 75 of whom had a BCR during follow-up. All patients were treated by RPx. The median follow-up for patients without event was 9 years. For clinicopathological characteristics, see Additional file 1: Table S2.

**Fig. 2:**
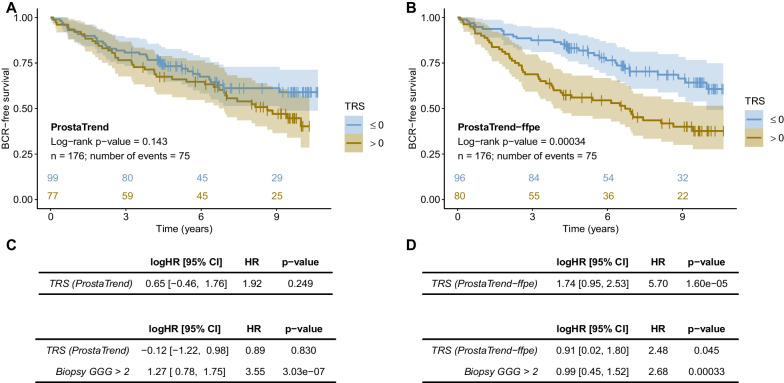
Prognostic value of ProstaTrend(-ffpe) in the FFPE biopsy cohort FFPE_Bx. We assessed the prognostic value of the ProstaTrend (**A**, **C**) and ProstaTrend-ffpe TRS (**B**, **D**) signature by Kaplan–Meier analysis and Cox proportional hazard regression (BCR as the primary endpoint). **A**, **B** Kaplan–Meier curves for patients with TRS > 0 (increased risk) compared to patients with TRS ≤ 0 (reduced risk). Color shades depict the 95% confidence intervals for Kaplan–Meier curves. The curves were truncated if the number of patients at risk dropped below 10 in both groups. The colored numbers above the x-axis indicate the number of patients at risk. Log-rank tests were performed to evaluate probabilities of BCR-free survival between these two groups. The numbers under the log-rank p-values indicate the number of patients and cases with BCR. **C**, **D** Univariate Cox-regression analysis for TRS on a continuous scale (top) and multivariable Cox-regression for TRS adjusted for Gleason grading group (GGG > 2) of the biopsies (bottom). *logHR* log hazard ratio, *HR* hazard ratio, *CI* confidence interval

After filtering out low expressed genes (see “Methods”), we applied the ProstaTrend TRS to each sample of the FFPE_Bx cohort as previously reported (Kreuz et al. 2020) (see “Methods”). Overall, there was no significant difference in time to BCR between patients with TRS > 0 and patients with TRS ≤ 0 in the FFPE specimens (Fig. 2A; p-value = 0.143; 5-year BCR-free survival: 66.1% [CI 56.2–77.6%] vs. 73.2% [CI 64.9–82.6%]). Cox-regression analysis of ProstaTrend on a continuous scale did not result in a significant association with prognosis (p-value = 0.249). When adjusted for biopsy GGG > 2 the model remained non-significant for ProstaTrend (p-value = 0.830) but resulted in a significant association of prognosis with GGG (p-value = 3.03 × 10^−7^; Fig. 2C).

Various systematic differences between the cohorts studied in Kreuz et al. (2020) may explain the poor reproducibility of the TRS in the FFPE_Bx cohort. In contrast to the training cohorts, FFPE_Bx included a substantial fraction of samples with low tumor cell content (median: 40%; range: 5–100%). Further, for FFPE_Bx, we multiplexed 32 samples for the first and 51 samples for the other 3 flow cells, resulting in a lower sequencing depth per sample compared to the training cohort (Kreuz et al. 2020). Thus sequencing depth is a potential confounding factor, especially for the measurement of ProstaTrend genes with low expression levels. However, filtering out of genes or samples associated with tumor cell content or low sequencing depth did not result in a relevant improvement in the prognostic accuracy of the TRS in FFPE_Bx (see Additional file 1).

Moreover, FFPE_Bx comprised FFPE preserved biopsy specimens in contrast to the training cohorts (Kreuz et al. 2020), which included mainly fresh-frozen specimens derived from radical surgeries. Thus, systematic differences in the degradation between FFPE and fresh-frozen conservation may affect the expression measurement of genes included in ProstaTrend, especially since the average age of the analyzed specimens was high for the FFPE_Bx cohort (median: 9.4 years). Filtering out of genes associated with specimen age (541 genes retained, see “Methods”) resulted in strong prognostic relevance of the TRS, also in FFPE_Bx (Cox-regression: p-value = 0.0025, see Additional file 1: Fig. S3F).

Given that the majority of samples in the ProstaTrend training cohorts were measured by expression microarrays and the primary endpoint was DoD, we expected improved prognostic accuracy if we filtered out genes that showed no consistent prognostic effects in RNA-Seq cohorts with BCR as primary endpoint. As we did not include the TCGA PRAD cohort (Abeshouse et al. 2015) in the training we wondered whether the prognostic association of the TRS would be strengthened when we restricted ProstaTrend to genes that also showed a consistent effect in TCGA PRAD. Removing genes showing inconsistent effects in TCGA PRAD (Cox-regression with p-value ≥ 0.1 or inconsistent logHRs) without considering the FFPE degradation effect also improved TRS, but to a lesser extent compared to filtering for FFPE degradation (Cox-regression: p-value = 0.013, see Additional file 1: Fig. S4).

When we applied both filters and restricted ProstaTrend to genes not associated with specimen age and with a consistent prognostic effect between the training cohorts and TCGA PRAD, the difference in time to BCR between patients with high and low TRS became even more pronounced (Fig. 2B). This new ProstaTrend-ffpe TRS encompassed 204 genes. Patients with high TRS had a significantly adverse clinical outcome compared to patients with low TRS (log-rank test: p-value = 0.00034; 5-year BCR-free survival: 56% [CI 46.2–68.1%] vs 81.9% [CI 74.4–90.1%]). Cox-regression analysis of the ProstaTrend-ffpe TRS revealed statistical significance (p-value = 1.60 × 10^–05^). In addition, the score remained an independent prognostic factor when adjusted for biopsy GGG > 2 (Fig. 2D, p-value = 0.045).

In conclusion, we removed genes from the ProstaTrend signature associated with FFPE specimen age and furthermore limited to genes that showed consistent prognostic relevance in another RNA-Seq cohort (TCGA PRAD) with time to BCR as prognostic endpoint. These filtering steps resulted in a newly formed, ProstaTrend-ffpe signature. TRS using the ProstaTrend-ffpe signature showed a prognostic significance in FFPE_Bx and retained its prognostic relevance when adjusted for Gleason Grade Groups. Additional file 2: Table S11 provides an overview of all ProstaTrend genes.

### Utilizing a single-cell atlas of human PCa tissue to relate TRS of ProtaTrend(-ffpe) to cell compartments

To characterize and analyze the expression of ProstaTrend genes with respect to cell types, we compiled a PCa single-cell transcriptome atlas from previously published datasets of 5 studies (Chen et al. 2021; Dong et al. 2020; Ma et al. 2020; Song et al. 2022; Tuong et al. 2021) (see Additional file 1: Fig. S14 and Table S7 for an overview of the datasets). After quality-control filtering of cells, integration, clustering and cell cluster annotation, the atlas contained data for about 90,000 cells from 41 donors derived from their PCa and, when available, from adjacent non-malignant tissue (n = 14 donors with matched tumor and adjacent tissue).

**Fig. 3:**
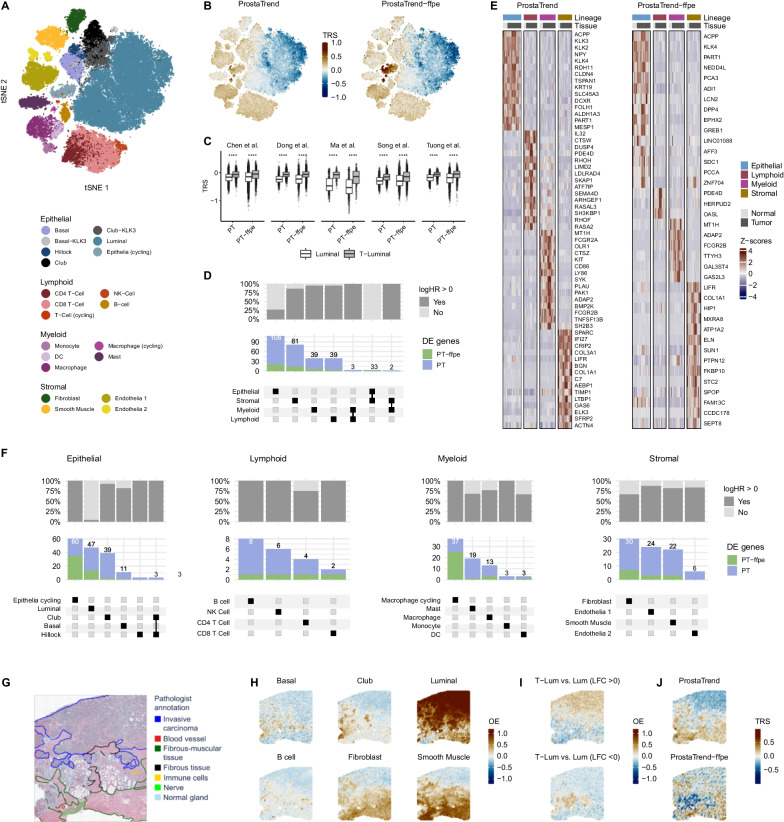
Analysis of ProstaTrend genes in the developed PCa single-cell atlas. **A** We embedded about 90,000 cells of 41 PCa patients from 5 publicly available datasets (Chen et al. 2021; Dong et al. 2020; Ma et al. 2020; Song et al. 2022; Tuong et al. 2021) into a two-dimensional space by the t-distributed stochastic neighbor embedding (tSNE) method. Each dot represents a single cell. Cells are colored according to cell identity. **B** We applied a simplified TRS to each cell from the tumor samples using the ProstaTrend and ProstaTrend-ffpe signatures. Each area containing cells on the tSNE was divided into hexagonal bins, and cells within each bin were averaged. The bins are colored according to TRS. **C** For tumor samples, TRS were grouped based on the ProstaTrend(-ffpe) signatures and colored according to luminal and tumor-specific luminal (T-luminal) cells (****p-value < 0.0001, Wilcoxon rank-sum test). *PT* ProstaTrend, *PT-ffpe* ProstaTrend-ffpe. The y-axis depicts the TRS. **D** UpSet plot of ProstaTrend(-ffpe) DE genes (FDR < 0.05) of four cell lineages. The squares in the matrix represent unique or overlapping DE genes for the cell lineages. The stacked bar graph above the matrix summarizes the number of ProstaTrend(-ffpe) DE genes for each unique lineage. The top stacked bar plot shows the fraction of DE genes with log hazard ratio > 0 or < 0. **E** Heat map of the highest ranked DE genes for the ProstaTrend(-ffpe) genes. Genes are ranked by log2 fold change. 15 genes (if present) are depicted for each cell lineage. Each column depicts the average expression value for one patient, grouped by cell lineage and tissue source. Average gene expression values are standardized. **F** UpSet plot of ProstaTrend(-ffpe) DE genes for cell types. **G** Pathological annotations of a human prostate stage III adenocarcinoma biopsy, which was FFPE preserved and processed using the Visium spatial gene expression for FFPE workflow. **H** Standardized overall enrichment (OE) scores of cell type markers for each spot were estimated using the AddModuleScore function implemented in Seurat. **I** OE of DE genes (T-luminal vs. luminal cells from the PCa Cell Atlas) for each spot. **J** TRS was applied to gene expression spots using the ProstaTrend(-ffpe) signatures

We annotated cell types following a three-step approach (see Additional file 1): first, we used the R package clustifyr (Fu et al. 2020), a correlation-based method for annotating cell clusters using single-cell/bulk data from healthy human prostate tissue (Henry et al. 2018) and hematopoietic cell types (Racle and Gfeller 2020; Villani et al. 2017) as reference. Second, we verified the annotated cell clusters based on the expression of canonical cell type markers. Third, if available, we matched our annotation results with the already annotated cells of the analyzed datasets. Overall, we identified 15 cell types derived from lymphoid, myeloid stromal and epithelial cell lineages in tumor and adjacent tissue (Fig. 3A). Basal cell and club cell populations were distinguished by sub-clusters that had high KLK3 (kallikrein related peptidase 3—also known as PSA) expression and were annotated with the suffix “KLK3” accordingly (Additional file 1: Fig. S23).

We applied a simplified TRS (see “Methods”) to each cell from the tumor samples using theProstaTrend(-ffpe) signatures. Figure 3B depicts the expression of TRS in cells from tumor samples. On average, risk scores calculated from the ProstaTrend(-ffpe) signatures were lower in luminal cells compared to other cell types (Additional file 1: Fig. S30). In addition, for the ProstaTrend-ffpe signature, we observed higher risk score in cycling cells (S, G2M phase) compared with the other cell types. Grouping TRS by GS and cell lineages revealed a positive correlation between TRS and GS for epithelial cells in 3 out of 4 studies (with heterogeneous GS), especially for luminal cells (Spearman correlation ProstaTrend-ffpe vs. GS in luminal cells: Chen et al. cor = 0.42; Song et al. cor = 0.12; Tuong et al. cor = − 0.02; Dong et al. cor = 0.56; Additional file 1: Fig. S31).

Furthermore, we used inferCNV (see Additional file 1 for methods) to estimate tumor-specific cells in epithelial cell types from tumor samples. We observed that tumor-specific luminal cells (T-luminal) had a significantly higher risk score than luminal cells in single-cell datasets of all studies (p-value < 0.05, Fig. 3C). We performed this analysis for all epithelial cell types but found the most significant effect across all studies in the luminal cell type (Additional file 1: Fig. S32).

We also compared ProstaTrend(-ffpe) with gene sets for 14 functional states of cancer (Yuan et al. 2019) (Additional file 1: Table S10). For 7 gene sets, we detected a significant enrichment of ProstaTrend genes, including metastasis, invasion, proliferation, EMT (Epithelial–mesenchymal Transition), cell cycle, DNA damage and DNA repair.

### Association of ProstaTrend genes with cell lineages and cell types

We associated ProstaTrend genes with cell lineages and cell types by performing DGEA utilizing the single-cell atlas for human PCa tissue. A Wilcoxon rank-sum test was applied between the average gene expressions per patient for each cell lineage against the average patient expressions of the other lineages (see Additional file 1 for details). Figure 3D depicts the number of significantly overexpressed ProstaTrend(-ffpe) genes (FDR < 0.05 and log fold change > 0.25). Most ProstaTrend genes were uniquely characteristic for epithelial (n = 108), followed by stromal (n = 81), myeloid (n = 39) and lymphoid cells (n = 39).

Approximately 73% of all ProstaTrend genes significantly differentially expressed (DE) in epithelial cells had a logHR < 0, i.e., high expression was associated with a good prognosis. In contrast, approximately 91% of DE genes associated with the other cell lineages were associated with adverse prognosis (logHR > 0). The 15 highest ranked significantly DE genes for each cell lineage and signature are depicted in Fig. 3E.

DGEA for cell types was performed in their respective cell lineage group (Fig. 3F). Additional file 1: Figures S27, S29 depict the highest ranked genes and the number of all DE genes, respectively. Of the 74 unique DE genes in the ProstaTrend-ffpe signature, 53% were associated with cycling cell types. Approximately 96% of all DE genes from the ProstaTrend(-ffpe) signatures associated with luminal cell types had a logHR < 0, consistent with the observation of low ProstaTrend(-ffpe) scores in luminal cells (Fig. 3B). DE genes associated with the luminal, club, cycling epithelial and cycling macrophage cell types were significantly (p-value < 0.05) enriched with genes from the ProstaTrend signature (Additional file 1: Table S9). All DE genes from the ProstaTrend signature and their respective assignment to cell lineages and cell types are included in Additional file 2: Tables S12, S13.

We also performed a DGEA between paired tumor and normal samples for each cell lineage and cell type. However, we did not observe significant expression differences which is consistent with the result of the study by Song et al. (2022).

### Relating the ProstaTrend(-ffpe) TRS to spatial transcriptomics of a PCa biopsy

We re-analyzed publicly available spatial transcriptome data from an FFPE-preserved PCa biopsy (GS = 3 + 4) to characterize ProstaTrend in the spatial context of prostate tissue. Pathological annotations for the tumor tissue were done at an overview level as shown in Fig. 3G. Each spot was scored for enrichment of cell type markers from the PCa cell atlas (see Additional file 1: Fig. S34 for enrichment of all cell types). Overall, we observed conformity between the pathological annotation and the cell type enrichment results (Fig. 3H). Gene sets for these cell types are included in Additional file 2: Table S14. Since primary prostate cancer has a luminal phenotype, we performed DGEA between T-luminal and luminal cells from the PCa single-cell atlas (see Additional file 1 for details). These DE genes were then used for enrichment analysis in the spots of the spatial sequencing sample. DE genes with log fold changes (LFC) > 0 were enriched in the invasive carcinoma regions of the tissue (p < 2.2e−16; see Additional file 1: Fig. S35), whereas DE genes with LFC < 0 were enriched in the normal glandular regions (p < 2.2e−16). See Fig. 3I and Additional file 2: Table S15 for gene sets. These results confirm the pathological annotation and indicate the transferability of the T-luminal signature to spatial transcriptomics.

As with the PCa cell atlas, we applied TRS to each spot using the ProstaTrend(-ffpe) signatures (Fig. 3J). We observed largely negative ProstaTrend risk scores in spots annotated as invasive carcinoma regions (Fig. 3G) and with high enrichment scores for luminal cells (Fig. 3H). On the other hand, TRS calculated from the ProstaTrend-ffpe signature showed a strongly negative score in normal glandular regions. Positive risk scores are mainly observed in stromal regions but also in spots enriched for leukocytes and partially in spots enriched for epithelial cells. Interestingly, a comparison of the TRS in Fig. 3J with the enrichment results from Fig. 3I shows that in both cases, spots enriched with T-luminal DE genes (LFC > 0 or < 0) had a negative TRS using ProstaTrend signatures.

### The significant association of the TRS with BCR is confirmed in 9 independent validation cohorts

To further validate the association of the TRS with BCR, we applied the ProstaTrend(-ffpe) TRS to publicly available PCa transcriptome datasets. We included studies with gene expression data available by microarray or RNA-Seq from primary PCa samples with available data on time to BCR or time to last follow-up if BCR had not occurred. In total 9 cohorts matched our criteria for inclusion (Li et al. 2020; Fraser et al. 2017; Luca et al. 2018; Long et al. 2014; Gerhauser et al. 2018; Jain et al. 2018; Ross-Adams et al. 2015; Taylor et al. 2010) (see “Methods”). Six of these were analyzed by gene expression microarrays and three by bulk RNA-Seq. The total number of patients included was n = 1109 and ranged from n = 73 to n = 248 per individual study. Additional file 1: Tables S1 and S3 provide a description of the included studies and the clinicopathological parameters, respectively. We performed survival analysis to assess the prognostic performance of TRS using the ProstaTrend(-ffpe) signatures for predicting time to BCR. For each cohort, the score was applied and dichotomized (TRS > 0 vs. TRS ≤ 0).

**Fig. 4:**
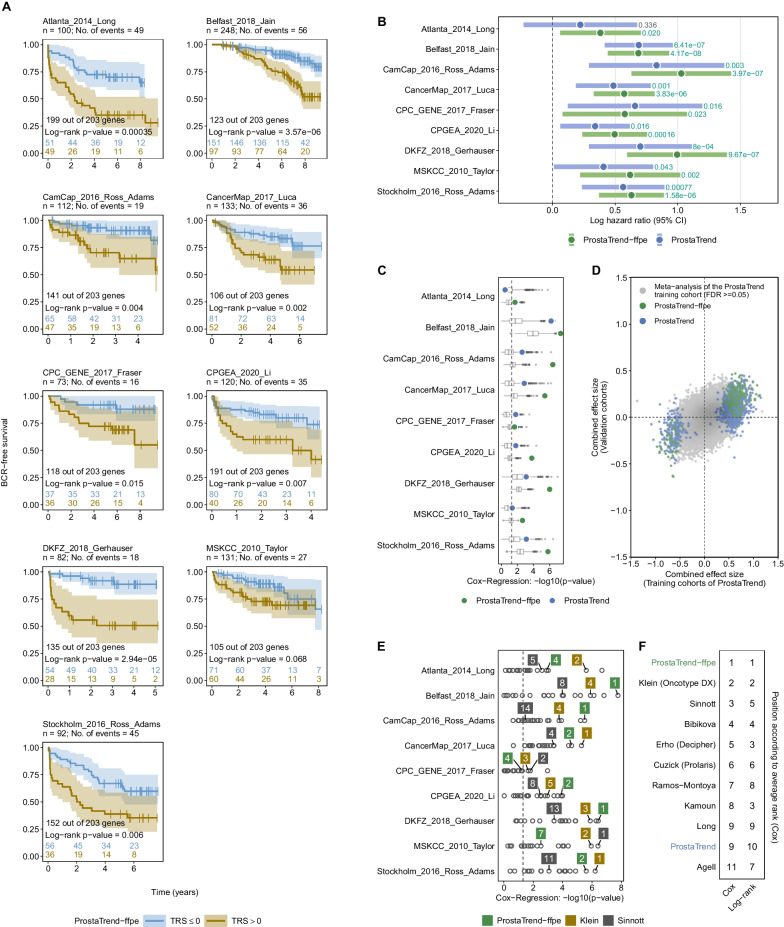
Validation of the ProstaTrend and ProstaTrend-ffpe signature in 9 publicly available PCa cohorts. **A** For the ProstaTrend-ffpe signature, Kaplan–Meier curves for patients with TRS > 0 (increased risk) compared to patients with TRS ≤ 0 (reduced risk) are shown. The numbers under the cohort IDs indicate the number of patients and cases with BCR. Color shades depict the 95% CI for Kaplan–Meier curves. The curves were truncated if the number of patients at risk dropped below 10 in both groups. The colored numbers above the x-axis indicate the number of patients at risk. Log-rank tests were performed to evaluate probabilities of BCR-free survival between the two groups. The numbers in the plot (above the log-rank p-values) indicate how many ProstaTrend-ffpe genes are available in the datasets. **B** Forest plot of the overall logHRs and corresponding 95% confidence intervals (95% CI) estimated by Cox-regression on a standardized continuous scale. Significant logHRs with a p-value < 0.05 are highlighted in green. **C** We generated 1000 random gene sets each for ProstaTrend and the ProstaTrend-ffpe signature, followed by Cox-regression analysis for each cohort (see “Methods”). The estimated −log10(p-value) for the random gene sets are shown as boxplots. The p-values for ProstaTrend(-ffpe) gene sets are shown as colored dots (same p-values as in **B**). **D** The x-axis depicts the combined effect size of logHRs for genes from a univariate random-effects meta-analysis approach of the ProstaTrend training cohorts. The same meta-analysis approach was performed for the 9 validation cohorts (y-axis). Genes whose logHRs from the meta-analysis of the training cohort showed no significance (FDR ≥ 0.05) are colored in gray. The logHRs of the ProstaTrend(-ffpe) genes are colored accordingly. **E** The dots depict the -log10(p-values) estimated from a Cox-regression model with a two-sided Wald test. Prognostic signatures that were among the top 5 (sorted by p-value) in more than 3 cohorts were highlighted by a label. The white numbers represent the rank by p-value. The dashed vertical line indicates a p-value of 0.05. **F** The average rank of each prognostic gene set across all cohorts from the log-rank and Cox-regression analysis

As shown in Fig. 4A, the Kaplan–Meier curves indicate a quantitatively strong difference in time to BCR for the ProstaTrend-ffpe TRS for the cohorts (for the original ProstaTrend see Additional file 1: Fig. S5). Statistical differences to assess the probability of BCR-free survival of patients with a TRS > 0 (increased risk) compared with patients with a TRS ≤ 0 (reduced risk) were evaluated using a log-rank test. For all cohorts except MSKCC_2010_Taylor, we observed significant differences (p-value < 0.05) between the two groups. Additional file 1: Figures S5 and S6 illustrate that the ProstaTrend-ffpe TRS showed a stronger association with time to BCR compared with ProstaTrend with respect to the log-rank test in all 9 cohorts.

Matched benign biopsies from PCa patients with at least 10 events were available for three cohorts, one from the ProstaTrend training cohorts (FF_array_RP) and two from the validation cohorts (CamCap_2016_Ross_Adams and CPGEA_2020_Li). We performed Kaplan–Meier analysis and log-rank test with dichotomized TRS using the ProstaTrend(-ffpe) signatures and observed only for CPGEA_2020_Li a consistent trend for a prognostic effect of ProstaTrend(-ffpe) in matched normal tissue (see Additional file 1: Fig. S7).

An overview of the estimated logHRs for TRS on a continuous scale is provided in Fig. 4B. ProstaTrend-ffpe was significantly associated with time to BCR in all 9 cohorts with p-values ranging from 4.17e^−8^ (Belfast_2018_Jain) to 0.02 (Atlanta_2014_Long). Consistently, increased TRS using the original ProstaTrend signature was associated with shorter time to BCR in all cohorts, although statistical significance was not reached for Atlanta_2014_Long (p-value = 0.336). For all cohorts except CPC_GENE_2017_Fraser, the association between time to BCR and TRS was more significant for ProstaTrend-ffpe compared to the original version.

In a multivariable Cox-regression analysis with adjustment for GS on a continuous scale, the ProstaTrend-ffpe TRS showed a consistent association with time to BCR for all validation cohorts (Additional file 1: Table S5), and statistical significance was achieved for all cohorts except MSKCC_2010_Taylor (p-value = 0.113) and Atlanta_2014_Long (p-value = 0.081). In 6 of the 9 analyzed cohorts, the association of prognosis with the ProstaTrend-ffpe TRS was more significant compared to the GS. Furthermore, using a Kruskal–Wallis test, we found a significant association between TRS and GS in all 9 cohorts (Additional file 1: Fig. S9). Significant differences in TRS between patients with low (pT1/2) and high (pT3/4) pathological tumor stages were observed in 5 of 8 cohorts (Additional file 1: Figs. S10, S11).

Next, we compared the prognostic prediction capabilities of the ProstaTrend(-ffpe) signatures with random gene sets. We randomly sampled 1000 gene sets of the same size as the number of ProstaTrend(-ffpe) genes detectable/passing the quality filtering criteria in that respective cohort, followed by Cox-regression analysis for each cohort (see “Methods”). The significance of the logHRs from the random gene sets is shown as boxplots for each cohort in Fig. 4C. The p-values for ProstaTrend(-ffpe) gene sets are depicted as colored dots. In all cohorts, the p-values of the ProstaTrend-ffpe signature were above the 75% percentiles of the boxplots of the random gene set. In 7 cohorts, the p-values of the ProstaTrend-ffpe signature were below all 1000 random gene sets (Fig. 4C, green dots).

Finally, we analyzed whether the directions of the estimated combined effect sizes of the logHRs from the ProstaTrend training cohorts (Kreuz et al. 2020) matched the combined effect size from the 9 validation cohorts (Fig. 4D). Concordance of the directions of the combined effect sizes was observed in 87% of all ProstaTrend genes and in 99% of the ProstaTrend-ffpe genes by univariate random-effect meta-analysis.

### Comparison of the prognostic performance of the ProstaTrend-ffpe with other prognostically relevant PCa panels revealed that ProstaTrend-ffpe was among the best-ranked panels

We compared the prognostic performance of ProstaTrend(-ffpe) with 17 other prognostically relevant PCa gene sets (see “Methods” and Additional file 1: Table S6). All genes in the panels were assigned binary information on whether their expression was associated with an increased or decreased risk of adverse outcome in the respective original publication. The corresponding table with all annotated genes of the panels is included in Additional file 2: Table S16. Using the cohort-wise standardized expression values of the validation cohorts, we applied for each gene set (including both ProstaTrend gene sets) and patient a simplified TRS, which was the median of all genes at increased risk minus the median of genes at reduced risk (see “Methods”).

We then performed a log-rank and Cox-regression analysis for each cohort to assess the prognostic performance of all gene sets. When we ranked the significance of the estimated logHRs by descending magnitude, we observed that the gene sets of the ProstaTrend-ffpe were among the 5 highest ranked panels in 8 cohorts (Fig. 4E and Additional file 1: Fig. S12A for all gene sets). On the log-rank test, ProstaTrend-ffpe were among the top 5 ranked panels in all 9 cohorts (Additional file 1: Fig. S12B). In Fig. 4F, we calculated the average rank of each prognostic gene set across all cohorts from the log-rank and Cox-regression analysis. We observed that the ProstaTrend-ffpe signature ranked first in the log-rank and Cox-regression analysis (see Additional file 1: Fig. S12C for the ranking of all gene sets).

The results demonstrate the prognostic relevance of our new ProstaTrend-ffpe signature and thus illustrate the added value of our signature. In addition, 155 of 204 genes in the ProstaTrend-ffpe signature do not occur in any other of the 17 prognostic signatures, underlining the novelty value of the prognostic genes (see Additional file 1: Fig. S12D).

### The majority of all ProstaTrend-ffpe genes showed significant combined effect sizes in a meta-analysis with all 13 cohorts

To assess the consistency and predictive performance of individual genes in the ProstaTrend(-ffpe) gene sets across all cohorts, we conducted a meta-analysis approach. The resulting model integrated all available samples for a best effort assessment of gene-wise combined effect size of logHRs, and significance of prognostic association. For the meta-analysis, we used the FFPE_Bx cohort, the TCGA PRAD cohort, the 9 validation cohorts and all training cohorts (Kreuz et al. 2020) with sufficient number of events (see “Methods”).

**Fig. 5:**
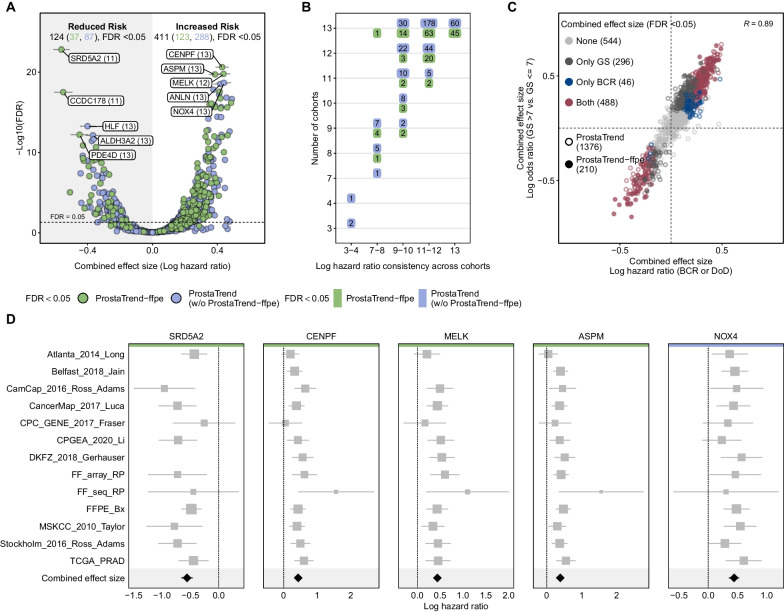
Results of the univariate random-effect meta-analysis using Cox proportional hazard models. **A** We estimated a combined effect size of logHRs for each gene across 13 cohorts. Shown are the combined effect sizes of all prognostic ProstaTrend genes (n = 1376). The x-axis represents the combined effect sizes and the y-axis the adjusted p-values (Benjamini–Hochberg correction for multiple testing) in log-space. The dashed horizontal line indicates an FDR of 0.05. For the highest ranked genes (by adjusted p-value) from the meta-analysis, 5 gene labels each with a combined effect size > 0 and < 0 are shown. The number in parentheses indicates the number of cohorts in which the respective gene was available. The numbers at the top of the plot indicate the number of significant ProstaTrend(-ffpe) genes with a combined effect size > 0 and < 0. **B** The y-axis shows the number of cohorts in which the ProstaTrend(-ffpe) genes had a significant combined effect size and were available. The x-axis indicates in how many cohorts the significant genes have a consistent direction of the logHRs. The labels indicate the number of genes in each case. **C** Scatter plot of all prognostic ProstaTrend genes (n = 1376). As in **A**, the x-axis represents the combined effect size of the logHRs. The y-axis represents the combined effect size of the log odds ratios derived from the logistic regression model predicting GS > 7 vs. ≤ 7. The meta-analysis for the log odds ratio was performed in the same way as for the logHRs. The colored dots indicate whether the combined effect sizes were significant (FDR < 0.05) in both meta-analyses approaches, in one meta-analysis or in none. The correlation coefficient is displayed in the upper right corner of the plot. **D** Forest plots for the 5 highest ranked genes by adjusted p-value. The logHR for each cohort is represented by a square. A horizontal line depicts the confidence interval (CI). The size of the square corresponds to the weight of the cohort in the meta-analysis. The combined effect and the CI are represented by a diamond. ProstaTrend-ffpe genes are highlighted with green colored horizontal bars; the blue ones are part of the original ProstaTrend signature

Of the total 1376 ProstaTrend genes, 535 genes (≈ 39%) had a significant combined effect size (FDR < 0.05), of which 124 genes had a combined effect size (logHR) < 0 and 411 > 0 (Fig. 5A). Overall, 160 of 203 (≈ 78%) ProstaTrend-ffpe genes had a significant combined effect size, of which 37 genes had a combined effect size < 0 and 123 > 0.

Considering the publicly available data sets were from different sources (see Additional file 1: Table S1), not all significant ProstaTrend genes were always present in all cohorts. Of 535 significant ProstaTrend genes, approximately 94% are annotated as protein-coding (data not shown). Of the significant genes available in fewer than 10 cohorts, the majority are annotated as non-coding and only 8% are protein-coding. This suggests a lower representation of non-coding genes across the cohorts studied and thus a lower statistical power to detect potential prognostic associations. Figure 5B indicates in how many cohorts the logHRs for the significant combined effect sizes had the same direction, i.e., > or < 0. Among 391 of the ProstaTrend genes whose significant combined effect sizes were composed of logHRs across all 13 cohorts, 346 genes showed a consistent logHR in more than 10 cohorts.

The 5 highest ranked ProstaTrend genes (by adjusted p-value) from the meta-analysis are shown as forest plots in Fig. 5D (see Additional file 1: Fig. S13 for a more detailed representation of the forest plots). SRD5A2, the gene with the highest significance level in our meta-analysis, encodes one of three isozymes of steroid 5 α-reductase, which catalyzes the conversion of testosterone to the more potent androgen dihydrotestosterone (DHT), the most active androgen in the prostate. In the case of PCa, increased androgen production stimulates androgen-dependent cancer cells. In advanced stages of metastasis, androgen dependence declines, and SRD5A2 expression has been observed to be downregulated in these cases (Titus et al. 2005, 2014; Kosaka et al. 2013; Söderström et al. 2001).

Lastly, we performed a second meta-analysis in which we estimated the combined effect size of log odds ratios derived from the logistic regression model to predict GS > 7 versus ≤ 7. We observed that 488 ProstaTrend genes (of which 154 are part of ProstaTrend-ffpe) had a significant combined effect size (FDR < 0.05) in both meta-analysis approaches (Fig. 5C). Moreover, we observed a spearman correlation coefficient of 0.89 between the effect sizes of both meta-analyses.

In summary, a large proportion of genes from the ProstaTrend-ffpe signature not only had significant combined effect sizes in the meta-analysis but also showed consistency in the direction of effect sizes across all cohorts analyzed. Results of the meta-analysis are included in Additional file 2: Tables S17, S18. Forest plots and other statistics on ProstaTrend genes from the meta-analysis can be analyzed at https://bioinf.izi.fraunhofer.de/prostatrend/.

## Discussion

We utilized a cohort of n = 176 PCa biopsy specimens to develop a transcriptome-based prognostic signature for FFPE-conserved specimens used in routine PCa diagnostic by selecting a gene subset from a previously described signature for fresh-frozen RPx-derived samples. For a substantial proportion of the genes selected for the prognostic score in fresh-frozen samples, we observed a strong correlation between gene expression and the time of FFPE conservation, indicating a relevant impact of FFPE-associated degradation on the measurement and consequently on the prediction of the prognosis in FFPE tissue. Filtering of these genes and removal of genes that are low expressed or inconsistent with the PCa dataset of the TCGA resulted in a strongly prognostic and FFPE biopsy-compatible novel ProstaTrend-ffpe signature that encompassed 204 genes. We observed excellent reproducibility of the prognostic relevance in nine publicly available studies demonstrating a high degree of robustness with respect to the measurement platforms used, tissue preservations, and differences in laboratory-specific processes. It should be noted that not all genes were available for all datasets, but this did not impair the reproducibility of the score. Based on the good reproducibility of the original ProstaTrend signature in other independent cohorts, we chose the approach of filtering genes whose expression signal was affected by FFPE conservation. This filtering process did not consider the prognostic endpoint (BCR) of the FFPE_Bx cohort to avoid potential overfitting. Alternatively, training a completely new signature considering all measured genes and cohorts would be possible but we refrained from this strategy as this would have led to an overestimation of the prognostic accuracy of the resulting score in the FFPE_Bx cohort.

PCa is a multifocal disease and considerable intra- and interfocal heterogeneity has been described (Carm et al. 2019). Evaluation of multiple foci of different gradings by molecular risk assessments showed significant differences in scoring (Carm et al. 2019). This also applies to transcriptome-based risk assessment for PCa (Salami et al. 2018). The results of our analyses already show a strong correlation of ProstaTrend-ffpe with prognosis in all analyzed cohorts. Despite the high number of genes included in the signature, molecular heterogeneity can be assumed to have a major influence on the assessment. We therefore expect an even better risk classification of ProstaTrend-ffpe if multiple foci of a patient will be analyzed in parallel. This could be achieved by utilizing imaging methods that support the detection of different foci.

Interestingly, the differences between ProstaTrend and ProstaTrend-ffpe TRS were less pronounced in the publicly available FFPE cohorts of Atlanta_2014_Long and Belfast_2018_Jain (see Additional file 1: Fig. S5) compared to the FFPE_Bx cohort used for filtering. Potential explanations could be the use of RPx specimens instead of biopsies in these cohorts or differences in preservation type or duration. Applying the ProstaTrend-ffpe TRS to matched benign prostate tissue from bulk transcriptomics indicated no or only a weak prognostic association. This indicates that the prognostic information is derived from the expression patterns of the tumor cells and/or tumor microenvironment. We demonstrate good concordance of the prognostic impact of individual genes between the ProstaTrend training (Kreuz et al. 2020) and validation cohorts (Fig. 4D). The higher absolute value of the estimated logHRs in the training cohorts is conceivably caused by the consideration of a different endpoint, i.e., DoD instead of BCR, as well as the enrichment of patients with events in the training cohorts. In addition, the smaller sample size as well as lower heterogeneity in the training cohort could lead to a higher absolute estimate of logHRs. Nevertheless, a large proportion of genes showed a reproducible association with prognosis suggesting that molecular processes with a multitude of associated genes are involved in an aggressive phenotype. This explains that a variety of gene expression signatures with discordant gene sets reproducibly reveal prognostic differences in patient cohorts (Fig. 4E and Additional file 1: Fig. S12). The analysis of ProstaTrend(-ffpe) and 17 published gene signatures for PCa prognosis indicated a comparatively very clear and reproducible prognostic relevance for ProstaTrend-ffpe. The public signatures are partly based on different clinical endpoints and have been established for different platforms with more specific weights and statistical models. Nevertheless, the simplified aggregation of single genes applied for the comparison of the signatures here represents a suitable and robust method to estimate the prognostic relevance of the signatures and is similarly applied, e.g., for the estimation of the Decipher score (Erho et al. 2013) in Salami et al. (2018). Although the standardization and simplification of the signatures obviously affect the results, our findings still indicate that the ProstaTrend-ffpe is among the signatures with the strongest prognostic prediction potential. The original ProstaTrend TRS described by Kreuz et al. (2020) was significantly associated with time to BCR in 8 of 9 of the validation cohorts, however the correlation was considerably weaker compared to the revised version, and it is not ranked among the best scores. The small sample size in the training cohort, the use of DoD as a clinical endpoint with enrichment for patients who experienced an event, and the focus on gene-expression microarrays might be factors that result in poorer generalizability of the original ProstaTrend TRS and worse performance in the validation cohorts. In conclusion, ProstaTrend-ffpe generally shows better prognostic performance than the original ProstaTrend signature and should also be applied for fresh frozen biopsy or RPx samples in the future.

The meta-analysis for the detection and validation of potential prognostic genes is limited by the availability of raw expression data and the lack of total-RNA sequencing data. Thus, data on novel and non-coding transcripts were not available for the majority of cohorts, and many relevant biomarkers belonging to these biotypes are likely to be missed in our analysis. The extent to which the prediction accuracy can be further improved by including these markers remains to be elucidated. However, in a total of 13 cohorts, a significant combined effect size was detected for 163 of 204 genes of the ProstaTrend-ffpe signature, underlining the prognostic relevance of the signature.

Even with the reduced number of included genes, ProstaTrend-ffpe still includes 204 genes which leads to high complexity and costs for the measurement. Other available signatures for PCa range from 3 to 222 genes (Additional file 1: Table S6). We expect with decreasing costs, transcriptome-wide analyses are also becoming applicable in clinical routine. Initial projects such as the WINTHER trial (Rodon et al. 2019) and the risk prediction for breast cancer using a 50-gene classifier (Parker et al. 2009) pave the way for a broader application. Furthermore, more comprehensive panels such as the FoundationOne®CDx assay (Frampton et al. 2013) including 324 genes are already being used successfully for the determination of genomic changes in solid tumors within molecular tumor boards. The application in clinical routine of the PCa panel described here is therefore not out of the question in the foreseeable future. To facilitate the application in the clinic, a further reduction of the gene signature might also be feasible. Analysis of the ProstaTrend-ffpe signature showed prognostic relevance in all analyzed validation cohorts even if not all signature genes were available in the cohorts (overlap ranging from 105 to 199 genes per cohort; see Fig. 4A). We investigated to what extent the prognostic accuracy decreases with a reduction of the signature size (Additional file 1: Fig. S8) and found only a small decrease in the concordance index for a reduction to a size of ≥ 50 genes. This indicates that a certain reduction in the size of the signature would be possible for the application in clinical routine with little loss in prognostic accuracy.

Our analyses confirm the added value for prognosis prediction with ProstaTrend-ffpe TRS compared to clinical and histological parameters alone. However, we demonstrated a strong association between the score and GS (Additional file 1: Fig. S9). Comparing the gene-wise meta-analysis with time to event endpoint (BCR and DoD) and a meta-analysis for GS > 7 vs GS ≤ 7 revealed strong correlation for the estimated overall effect sizes (Fig. 5C). This association is also reflected in the scRNA-Seq analyses, where the correlation of the GS and the transcriptomic risk score was observed in epithelial cells and, in particular, luminal cells. In addition, the ProstaTrend(-ffpe) TRS was increased in luminal tumor cells compared to normal luminal cells. Interestingly, scores were generally low in epithelial and luminal cells in single-cell and spatial RNA sequencing data compared to other cell lineages. However, due to the small number of patients, further studies are needed to validate this association in single-cell data. The association of expression patterns and GS suggests that the degree of differentiation of the tissue is reflected in both the gene expression of the tissue as well as the histological appearance of the glands. It would be interesting to investigate to what extent information on localization and the spatial extension of the tumor, for example, by means of imaging data, can provide complementary prognostic information and thus further improve the prediction. A comparison of imaging data (mpMRI) with a molecular assessment of the tumor by Leapman et al. resulted only in a weak agreement between mpMRI and biomarker data suggesting indeed complementary information for these methods (Leapman et al. 2017).

The prognostic relevance of individual genes provided in the analyses presented here can be investigated via a web interface (https://bioinf.izi.fraunhofer.de/prostatrend/). In addition, the role of individual genes in spatial or scRNA-Seq data of PCa can be explored, and thereby, we provide a valuable resource to the research community.

## Conclusions

In conclusion, we provide a detailed overview of the relationship of gene expression landscape and prognosis in early PCa. We present a new version of the ProstaTrend signature consisting of 204 genes predicting clinical outcome of PCa based on FFPE preserved biopsies, suitable to support clinical decision-making. The signature complements clinical and pathologic prognostic information and is competitive with already existing signatures. Furthermore, we have developed a PCa single cell atlas to analyze the expression patterns of prognostic genes in different cellular compartments, which will be an important resource for future studies of PCa.

### Supplementary Information

**Additional file 1. **This file contains Additional Methods, Figures S1–S35 and Tables S1–S10.

**Additional file 2: Table S11.** Overview of the ProstaTrend(-ffpe) genes. **Table S12.** PCa cell atlas: association of ProstaTrend(-ffpe) genes with cell lineages. **Table S13.** PCa cell atlas: association of ProstaTrend(-ffpe) genes with cell types. **Table S14.** PCa cell atlas: cell type specific marker. **Table S15.** PCa cell atlas: tumor-specific luminal cells (T-luminal) vs. luminal cells. **Table S16.** Prognostic PCa gene signatures. **Table S17.** Meta-analysis. Endpoint: biochemical recurrence (BCR). **Table S18.** Meta-analysis. Endpoint: Gleason score. **Table S19.** Clinicopathological characteristics of patients from the FFPE_Bx RNA-Seq cohort.

## Data Availability

RNA-Seq and microarray datasets analyzed in this study are available in the NCBI GEO database (https://www.ncbi.nlm.nih.gov/geo/) under the accession numbers GSE84042 (CPC_GENE_2017_Fraser), GSE21034 (MSKCC_2010_Taylor), GSE94767 (CancerMap_2017_Luca), GSE70768 (CamCap_2016_Ross_Adams), GSE70769 (Stockholm_2016_Ross_Adams), GSE54460 (Atlanta_2014_Long) and GSE116918 (Belfast_2018_Jain). The cohort DKFZ_2018_Gerhauser is available at https://www.cbioportal.org/ and CPGEA_2020_Li at http://www.cpgea.com. Normalized gene expression counts for the FFPE_Bx cohort are available under accession number GSE220095 (https://www.ncbi.nlm.nih.gov/geo/query/acc.cgi?acc=GSE220095). scRNA-seq datasets are available under the accession numbers GSE141445 (Chen et al.), GSE137829 (Dong et al.), GSE157703 (Ma et al.) and GSE176031 (Song et al.). Read counts from the study Tuong et al. were obtained from https://www.prostatecellatlas.org. An interactive version of the results of this study can be accessed at https://bioinf.izi.fraunhofer.de/prostatrend/.

## Abbreviations

BCR: Biochemical recurrence
cDNA: Complementary DNA
CNV: Copy number variation
DE genes: Significantly differentially expressed
DGEA: Differential gene expression analysis
DOR: Diagnostic odds ratios
DoD: Death of disease
FF: Fresh-frozen
FFPE: Formalin-fixed paraffin-embedded
FFPE_Bx: Formalin-fixed paraffin-embedded biopsy
FPKM: Fragments per kilobase million
GGG: Gleason Grade Group
GS: Gleason score
LFC: Log fold change
logHR: Log hazard ratio
PCa: Prostate cancer
PSA: Prostate-specific antigen
RNA-Seq: RNA-sequencing
RPx: Radical prostatectomy
RPKM: Reads per kilobase million
scRNA-Seq: Single-cell RNA-sequencing
TCGA: The cancer genome atlas
TRS: Transcriptomic risk score
Vst: Variance stabilizing transformation
