# Supplementary material for: A reliable transcriptomic risk-score applicable to formalin-fixed paraffin-embedded biopsies improves outcome prediction in localized prostate cancer
Source: Mol Med. 2024 Feb 1;30:19. doi: 10.1186/s10020-024-00789-9 (PMC10835874; doi:10.1186/s10020-024-00789-9)
Supplement: Supplementary file 1 — Additional file 1. This file contains Additional Methods, Figures S1–S35 and Tables S1–S10. [file 10020_2024_789_MOESM1_ESM.pdf]

Supplementary material

A reliable transcriptomic risk-score applicable to Formalin-Fixed Paraffin-Embedded biopsies improves outcome prediction in localized prostate cancer

I ProstaTrend-ffpe and validation 1

1 Data sources 1
1.1 Overview of technical and clinicopathological parameters . . . 1
1.2 Internal cohort (FFPE_Bx) . . . 5
1.2.1 RNA isolation from FFPE specimens . . . 5
1.2.2 RNA sequencing of FFPE specimens . . . 5

2 Pre-processing of transcriptome data 5
2.1 Internal cohort (FFPE_Bx) . . . 5
2.2 External cohorts . . . 8

3 Confounding factors and gene filtering (FFPE_Bx) 9
3.1 Impact of reduced sequencing depth . . . 9
3.2 Impact of tumor cell content . . . 10
3.3 Impact of FFPE specimen age . . . 11

4 Prognostic value of ProstaTrend in validation cohorts 14
4.1 Survival analysis of ProstaTrend-ffpe using TRS. . . . 14
4.2 TRS for primary tumor and matched healthy tissue . . . 16
4.3 Multivariate Cox proportional hazard model . . . 18
4.4 Association between the TRS and clinicopathological parameters . . . 19
4.5 Published gene expression-based signatures for PCa prognosis . . . 21

5 Meta-analysis 23

II Prostate cancer single-cell atlas 24

6 Pre-processing 24

7 Analysis strategy 24
7.1 Integration and clustering . . . 25
7.2 Cluster annotation . . . 26

8 Differential expression analysis (DGEA) 27
8.1 Cell types/lineages 27
8.2 Tumor-specific luminal (T-luminal) vs. luminal cells 28

9 Evaluation of cell type/lineage specific markers 28

10 Tumor cell identification using inferCNV 29

11 Analysis workflow 29
11.1 First iteration: Post-clustering filtering 29
11.2 Second iteration: QC and cell cluster annotation 30
11.2.1 Coarse grain cell cluster annotation 34
11.2.2 Fine grain cell cluster annotation 34
11.3 Final PCa atlas 40

12 TRS applied to cells from the PCa atlas 44

13 Enrichment scores of gene sets for functional states of cancer 47

III Spatial transcriptomics data analysis 48
13.1 Enrichment scores of gene sets for cell types 48
13.2 Evaluation of spots associated with invasive cancer 49
13.2.1 Integration and clustering 49
13.2.2 Carcinoma associated cluster 49
13.2.3 Spots associated with luminal cells 49

References 52

List of Figures

S1 Quality Control of samples from the FFPE_Bx cohort with respect to sequencing library composition, read alignment . . . 8
S2 Principal component analysis of all tissue specimens, either assessed by transcriptome-wide next-generation sequencing or by microarrays after standardization of normalized expression values . . . 10
S3 Filtering of ProstaTrend for genes that may impair reproducibility of the Transcriptomic Risk Score (TRS) in the UKPD cohort . . . 12
S4 Filtering of ProstaTrend for genes using TCGA_PRAD. . . . 13
S5 Kaplan-Meier analysis of the ProstaTrend-ffpe Transcriptomic Risk Score (BCR as the primary endpoint) . . . 14
S6 Prognostic value of Transcriptomic Risk Score using the ProstaTrend-ffpe signatures by log-rank test . . . 15
S7 Kaplan-Meier analysis of the ProstaTrend-ffpe Transcriptomic Risk Score in 3 cohorts with primary tumor and matched benign/healthy tissue . . . 16
S8 Comparison of concordance index (c-index) in relationship to signature size. . . . 17
S9 Association between the ProstaTrend-ffpe TRS with Gleason score . . . 19
S10 Association between the ProstaTrend-ffpe TRS with pathological stage . . . 20
S11 Association between the ProstaTrend-ffpe TRS with lymph node metastasis . . . 20
S12 Comparison of the prognostic performance of ProstaTrend with other prognostically relevant PCa panels . . . 22
S13 Forest plots of the 5 highest ranked genes from the meta-analysis using a univariate random-effect model . . . 23
S14 Overview of scRNA-Seq datasets used to develop the PCa cell atlas . . . 24
S15 Metrics for quality control of analyzed samples . . . 25
S16 Principal component analysis (PCA). . . . 26
S17 Post-cluster cell filtering . . . 30
S18 Dimensionality reduction after integration . . . 31
S19 Entropy Metric to evaluate integration . . . 32
S20 Expression of leukocytic markers . . . 32
S21 Expression of stromal and epithelial markers . . . 33
S22 Cluster cell annotations of epithelial and stromal cell types and leukocyte lineage . . . 36
S23 DGEA for basal and club cell types characterized by sub-clusters with high KLK3 expression . . . 37
S24 Cluster cell annotations of leukocyte cell types . . . 37
S25 Identification of sub-clusters and cell type annotation for myeloid cells . . . 38
S26 Identification of sub-clusters and cell type annotation for T-cells and NK-cells . . . 39
S27 PCa single cell atlas . . . 41
S28 Inter-patient and study heterogeneity per cell type . . . 42

S29 DGEA for cell lineages/types 43
S30 TRS for each cell types 44
S31 TRS grouped by Gleason scores and colored by cell lineages 45
S32 TRS in tumor specific and non-tumor epithelial cells 46
S33 TRS for each cell using gene sets for functional states of cancer 47
S34 Enrichment of cell types in spatial transcriptomic data from a human prostate biopsy. 48
S35 Integration analysis of spatial transcriptome data from human normal and cancerous prostate biopsies 51

List of Tables

S1 Overview of the technical parameters of the datasets analyzed in this study 2
S2 FFPE_Bx and TCGA_PRAD cohorts: Overview of the clinico-pathological parameters . 3
S3 Validation cohorts: Overview of the clinicopathological parameters 4
S4 References used for FastQ Screen 8
S5 Multivariate Cox proportional hazard regression for all validation cohorts 18
S6 Prognostic PCa gene signatures 21
S7 PCa cell atlas: Overview of the clinicopathological parameters 25
S8 Test and reference cell lineages for inferCNV 29
S9 Enrichment of DE gene with ProstaTrend genes 43
S10 Gene set enrichment analysis 47

Part I. ProstaTrend-ffpe and validation

1 Data sources

1.1 Overview of technical and clinicopathological parameters

Internal cohort (validation of ProstaTrend)

Study ID	PMID	Data Source	Platform	Conservation method	Source of biospecimen	Datatype	Notes
FFPE_Bx		GSE220095	Illumina HiSeq 2500 (126bp paired-end); GPL16791	FFPE	Biopsy	VST<br>DESeq2 R package	

External cohort (gene filtering of ProstaTrend)

Study ID	PMID	Data Source	Platform	Tissue	Surgical treatment	Datatype	Notes
TCGA_PRAD	26544944	GDC Data Portal	Illumina HiSeq	Fresh-Frozen	RPx	VST<br>DESeq2 R package	Samples with less or equal 50% tumor cells were filtered out

External cohorts (validation cohorts)

Study ID	PMID	Data Source	Platform	Tissue	Surgical treatment	Datatype	Notes
Atlanta_2014_Long	24713434	GSE54460	Illumina HiSeq 2000 (50bp paired-end); GPL11154	FFPE	RPx	VST<br>DESeq2 R package	Duplicates from six patient samples were removed
Belfast_2018_Jain	29045551	GSE116918	Almac Diagnostics Prostate Disease Specific Array (DSA); GPL25318	FFPE	Biopsy	RMA<br>R package unknown	
CamCap_2016_Ross_Adams	26501111	GSE70768 (GSE70770)	Illumina HumanHT-12 V4.0; GPL10558	Fresh-Frozen	RPx	Log2 transformed and quantile normalized (Beadarray R package)	CampCap: 8 patients have no BCR event but a record of time to BCR. In these cases, the last follow-up was used instead.
Stockholm_2016_Ross_Adams	26501111	GSE70769 (GSE70770)	Illumina HumanHT-12 V4.0; GPL10558	Fresh-Frozen	RPx	Log2 transformed and quantile normalized Beadarray R package	
CancerMap_2017_Luca	28753852	GSE94767	Affymetrix Human Exon 1 0 ST; GPL5175	Fresh-Frozen	RPx	RMA<br>Oligo R package	1) The CamCap and CancerMap datasets have in common 40 patients and thus are not independent.<br>2) As proposed in the publication, when several samples per patient were available, only the sample with the highest proportion of tumour tissue was used.<br>3) As suggested by the authors, the R package sva was used to correct for batch effects between different centres (see NCBI GEO).
CPC_GENE_2017_Fraser	28068672	GSE84042	Affymetrix Human Transcriptome Array 2.0 and Gene 2.0 ST Array; GPL16686, GPL17586	Fresh-Frozen	RPx	RMA<br>Oligo R package	As suggested by the authors, the R package sva was used to correct for batch effects between different arrays.
CPGEA_2020_Li	32238934	cpgea.com	HiSeq X TEN (150bp paired-end)	Snap-Frozen	RPx	FPKM	
DKFZ_2018_Gerhauser	30537516	cBioPortal	Illumina HiSeq 2000 (50bp paired-end)	Fresh-Frozen	RPx	RPKM	When several samples per patient were available, only the first replicate was used (Replicates with suffix .+ T01)
MSKCC_2010_Taylor	20579941	GSE21034	Affymetrix HuEx 1.0 ST; GPL10264	Fresh-Frozen	RPx	RMA<br>Oligo R package	

Table S1: Overview of the technical parameters of the datasets analyzed in this study. RPx = Radical Prostatectomy; FFPE = Formalin-Fixed, Paraffin-Embedded; VST = Variance Stabilizing Transformation (using the R package DESeq2); RMA = Normalization and probe set summarization was performed using the Robust Multi-Array average algorithm (Log2 transformed and quantile normalized); FPKM = Fragments Per Kilobase Million. FPKM were transformed to log space and quantile normalized; RPKM = Reads Per Kilobase Million. RPKM were transformed to log space and quantile normalized

	**FFPE Bx** (n=176)	**TCGA PRAD** (n=332)
Age (years)	67 (61.9-70.5)	62 (56-66)
total PSA (ng/ml)	8.2 (5.6-13.9)<br>pre-biopsy	7.6 (5.2-12.1)<br>missing:26<br>pre-surgery
Pathological tumor stage	pT2: 117<br>pT3: 46<br>pT4: 13	pT2: 111<br>pT3: 207<br>pT4: 7<br>missing: 7
Organ confinement	Yes: 117<br>No: 59	Yes: 111<br>No: 214<br>missing: 7
RPx Gleason Score	GS 6: 35<br>GS 7a: 83<br>GS 7b: 37<br>GS 8: 5<br>GS 9: 15<br>GS 10: 1	GS 6: 26<br>GS 7a: 96<br>GS 7b: 64<br>GS 8: 43<br>GS 9: 101<br>GS 10: 2
Lymph node metastases	pN0: 146<br>pN1: 22<br>missing: 8	pN0: 256<br>pN1: 24<br>missing: 52
Resection status	R+: 25<br>R0: 151	R+:106<br>R0:208<br>RX:10<br>missing:8
Follow-up time	9.0 (5.7-10.0)	1.5 (0.7-2.8)
Biochemical Recurrence	No: 101<br>Yes: 75	No: 290<br>Yes: 42
Death from disease	Alive: 160<br>Death of disease: 5<br>Other: 9<br>missing: 2	Alive:328<br>Death of disease:4
Tumor cell content	40 (20-60)<br>missing:2	70 (60-80)

Table S2: FFPE_Bx and TCGA_PRAD cohorts: Overview of the clinico-pathological parameters. All numbers refer to individual patient samples and are reported as the median and interquartile range: [median (first quartile - third quartile)] or as categories: [category: number of observations]. **Age** of patients at the time of RPx or at initial pathological diagnosis in the TCGA PRAD cohort. **Pathological tumor stage:** the pathological tumor stage classification (pT) according to the TNM staging. **Organ confinement:** the confinement of the tumor within the prostate, i.e. pathological stage=pT2. **Lymph node metastases:** status of tumor lymph node infiltration are divided into the category of regional lymph node metastases (pN1), or if the patient was free of lymph node metastases (pN0). **Follow-up time in years:** time of follow-up in years for patients without event. **Biochemical Recurrence:** status of the clinical endpoint BCR defined for FFPE_Bx (namely, as a PSA value $\geq$ 0.2 ng/mL after RPx) and for TCGA_PRAD (as defined by the TCGA network group). Patients without BCR were censored at last follow-up. **Death of disease:** status of the clinical endpoint. It is reported as the number of patients who died because of PCa (Death of Disease), another reason (Other) or if the patient survived within the follow-up time (Alive). **Tumor cell content** was evaluated for each analyzed biopsy (FFPE_Bx). Assessment of tumor cell content for samples of the TCGA_PRAD cohort was described previously by the TCGA research network [1].

Characteristic	Atlanta 2014_Long No.=100	Belfast 2018_Jain No.=248	CamCap 2016_Ross_Adams No.=112	CancerMap 2017_Luca No.=133	CPC 2017_Fraser No.=73	CPGEA 2020_Li No.=120	DKFZ 2018_Gerhauser No.=82	MSKCC 2010_Taylor No.=131	Stockholm 2016_Ross_Adams No.=92
**Age (years)**									
Median (IQR)	62 (57, 66)	68 (63, 72)	62 (56, 65)	NA (NA, NA)	61 (57, 64)	69 (65, 74)	48 (45, 49)	58 (54, 62)	NA (NA, NA)
Not available	0	0	0	133	0	0	0	0	92
**Follow-up (years)**									
Median (IQR)	6.2 (5.3, 8.1)	7.2 (6.3, 8.3)	2.6 (1.5, 4.1)	4.9 (4.2, 5.7)	6.3 (5.7, 8.4)	2.4 (1.9, 3.5)	3.1 (2.1, 4.1)	4.2 (3.1, 5.3)	6.6 (5.3, 7.2)
**Biochemical Recurrence**	49	56	19	36	16	35	18	27	45
**Pathological stage**									
pT1/pT2	81	NA	35	73	40	61	56	85	47
pT3/pT4	18	NA	77	60	33	59	26	46	42
Not available	1	248	0	0	0	0	0	0	3
**Clinical stage**									
cT1/cT2	NA	127	96	NA	NA	94	NA	126	81
cT3/cT4	NA	96	16	NA	NA	23	NA	5	9
Not available	100	25	0	133	73	3	82	0	2
**Pathological lymph node status**									
pN0	NA	NA	83	NA	NA	87	NA	102	18
pN1	NA	NA	8	NA	NA	14	NA	6	NA
pNX	NA	NA	20	NA	NA	19	NA	23	72
Not available	100	248	1	133	73	0	82	0	2
**Clinical lymph node status**									
cN0	NA	NA	39	NA	NA	107	NA	NA	NA
cN1	NA	NA	2	NA	NA	11	NA	NA	NA
cNX	NA	NA	69	NA	NA	2	NA	NA	NA
Not available	100	248	2	133	73	0	82	131	92
**Gleason score**									
5	1	0	0	0	0	0	0	0	2
6	10	42	17	37	16	10	11	41	18
7	75	99	86	85	57	56	61	74	55
8	10	52	8	4	0	18	1	8	5
9	4	54	1	7	0	36	8	7	9
10	0	1	0	0	0	0	1	0	1
Not available	0	0	0	0	0	0	0	1	2
**PSA level (ng/mL)**									
<=10	67	50	84	95	57	26	48	100	62
>10 & <= 20	19	95	26	32	15	34	14	20	21
> 20	11	103	1	4	1	60	20	10	7
Not available	3	0	1	2	0	0	0	1	2

Table S3: Validation cohorts: Overview of the clinicopathological parameters. Age: age at diagnosis; Follow-up: time of follow-up in years for patients without event. [p|c]N0: without lymph node metastasis; [p|c]N1: withlymph node metastasis; [p|c]NX: Regional lymph nodes cannot be assessed; PSA: prostate-specific antigen; NA = not available. Datasets that met the following inclusion criteria for PCa datasets were incorporated in this study. 1) The patients in the cohorts must have a complete record of time to BCR or time to last follow-up if BCR has not occurred. 2) Tumors must be derived from the primary site. 3) Gene expression data must be available (Microarray, RNA-Seq). Other samples that we excluded from the cohorts are justified in Table S1 (see column "Notes"). This table contains the patient characteristics that meet the above conditions.

1.2 Internal cohort (FFPE_Bx)

1.2.1 RNA isolation from FFPE specimens

From up to five FFPE tissue slices of 10 μm we isolated RNA using Deparaffinization Solution (Qiagen) according to the miRNEASY protocol. A pathologist evaluated HE-stained flanking tissue slices and tumor content was assessed, ranging between 5% and 100% (mean= 41%). After subsequent proteinase K digestion and formalin-de-crosslinking, we transferred the samples onto RNeasy MinElute spin columns. We used the miRNeasy FFPE kit on the QIAcube (Qiagen) following the manufacturer's instruction for the final RNA purification. To eliminate all traces of genomic DNA, we performed DNAse-digestion (TURBO DNA free Kit, Thermo Fisher) twice in each sample. The RNA was concentrated using a standard precipitation protocol if necessary. Finally, we quantified extracted RNA using a Qubit RNA-Kit and the Qubit 2.0 instrument (Thermo Fisher). We analyzed the quality of RNA on a Bioanalyzer 2100 instrument (Agilent Technologies) and calculated the DV200 values (the percentage of fragments >200 nucleotides), ranging between 5% and 63% (mean=37%). Because of the well-known quality issues of old FFPE specimens, we decided to include all samples in further analysis.

1.2.2 RNA sequencing of FFPE specimens

For RNA-sequencing of FFPE specimens, we used 200 ng RNA per sample (when available). We prepared RNA libraries using TruSeq-Stranded Total RNA Sample Prep kit (Illumina) including a rRNA depletion step according to manufacturers' protocol, i.e. Ribo-Zero Gold rRNA Removal Kit. Following Illumina's recommendation, we skipped the fragmentation step for all samples. We analyzed the quality and quantity of each prepared library with the Denovix instrument (Qubit DNA-Kit) and with the Bioanalyzer 2100, calculated the molarity of each library and pooled equal amounts which were used for subsequent sequencing (12 pM). We sequenced 2 x 126-bp paired-end reads using SBS V4 chemistry with a HiSeq 2500 (Illumina) on four eight-lane flow cells: the first one with 32 and the others with 51 libraries each (185 in total).

2 Pre-processing of transcriptome data

2.1 Internal cohort (FFPE_Bx)

To facilitate the multi-step analysis of the RNA sequencing datasets, we applied the workflow-manager uap v0.0.1 [2].

Primary and secondary data analysis: Demultiplexing of Illumina raw files was performed with the Illumina `bcl2fastq` software v2.19 [3]. The paired-end FASTQ reads were trimmed and filtered using `AdaptorRemoval` v.2.2.1a [4] with additional parameters to trim ambiguous bases (N) at 5'/3' termini (–trimns), remove low-quality bases (–trimqualities, –minquality 20) and keep reads with a minimum read length of 30bp (–minlength 30). Trimmed reads were mapped to the human

reference genome version (GRCh38/hg38) using HISAT2 v2.1.0 [5]. Gene level quantification was performed using HTSeq v0.11.2 [6] with additional parameter (–mode intersection-strict).

To ensure that the putative novel intergenic genes from the ProstaTrend signature [7] are also represented in the FFPE_Bx cohort, we extended the human reference gene annotation GENCODE (release 36 GRCh38.p12) as follows: we used the annotation of expression quality filtered novel transcripts classified as “u”(unknown intergenic) of the cohort FF_seq_RP as described previously [7]. Since these annotations are based on GRCh37/hg19, we updating the annotation using UCSC liftOver tool [8] to GRCh38/hg38 (Chain file: http://hgdownload.soe.ucsc.edu/goldenPath/hg19/liftOver/hg19ToHg38.over.chain.gz). After converting the coordinates all prognostic genes from the ProstaTrend signature (classified as unknown intergenic and FDR <0.05) could be retained.

Quality control: Sample QC was reported using FastQC v0.11.8 [9] to assess base call accuracy, Preseq v2.0.3 [10] to evaluate the library complexity. Duplication metrics were collected with the MarkDuplicates function of Picard tools v2.18.29 (http://broadinstitute.github.io/picard/) using the BAM files generated by HISAT2. Picard’s CollectRnaSeqMetrics was used to collect mapping percentages on intergenic, intronic, coding and UTR regions as well as gene body coverage. RSeQC v3.0.0 [11], was used to determine, read GC content, junction saturation, read pair inner distance, and strandness of reads. FastQScreen v0.14.0 (–subset 1000000) [12] in conjunction with bowtie2 v2.3.4 [13] was conducted to assess RNA library composition (see Table S4 for references). For each sample a subsample of 1 million trimmed paired-end reads was randomly chosen by fastq-sample v0.8 [14]. The fraction of reads mapped to human rRNA (Table S4) in sense or antisense was determined using bowtie2. The median insert size was estimated by Qualimap v2.2.1 [15] using the BAM files. Aggregated data visualization for the secondary analysis and quality control were generated using the MultiQC [16] framework. A quality control summary report is shown in Figure S1.

Sample filtering: 6 samples excluded after quality filtering of the RNA seq data. Of these sample 3 have low sequencing depth of less than 5 million read pairs after trimming. (Figure S1D), 2 samples have a high rRNA content of more than 25% mapped reads against the human rRNA database (Figure S1C). More than 99% of the rRNA reads of these 2 samples correspond to rRNA and therefore affect the assessment of transcriptome variation (Figure S1F). The last excluded sample has a high insert size of more than 1000 bp (Figure S1G).

In contrast, samples with a small percentage (15-20%) of reads mapping mainly antisense to rRNA genes resemble antisense probes from the rRNA depletion step and therefore do not affect the assessment of transcriptome variation. Therefore, these samples were not excluded.

Normalization: Gene counts were adjusted for library size and normalized with the variance-stabilizing transformation (vst) as implemented in DESeq2 v1.30.1 [17]. The vst method was run with the option “blind = TRUE” to compare samples in an unbiased manner.

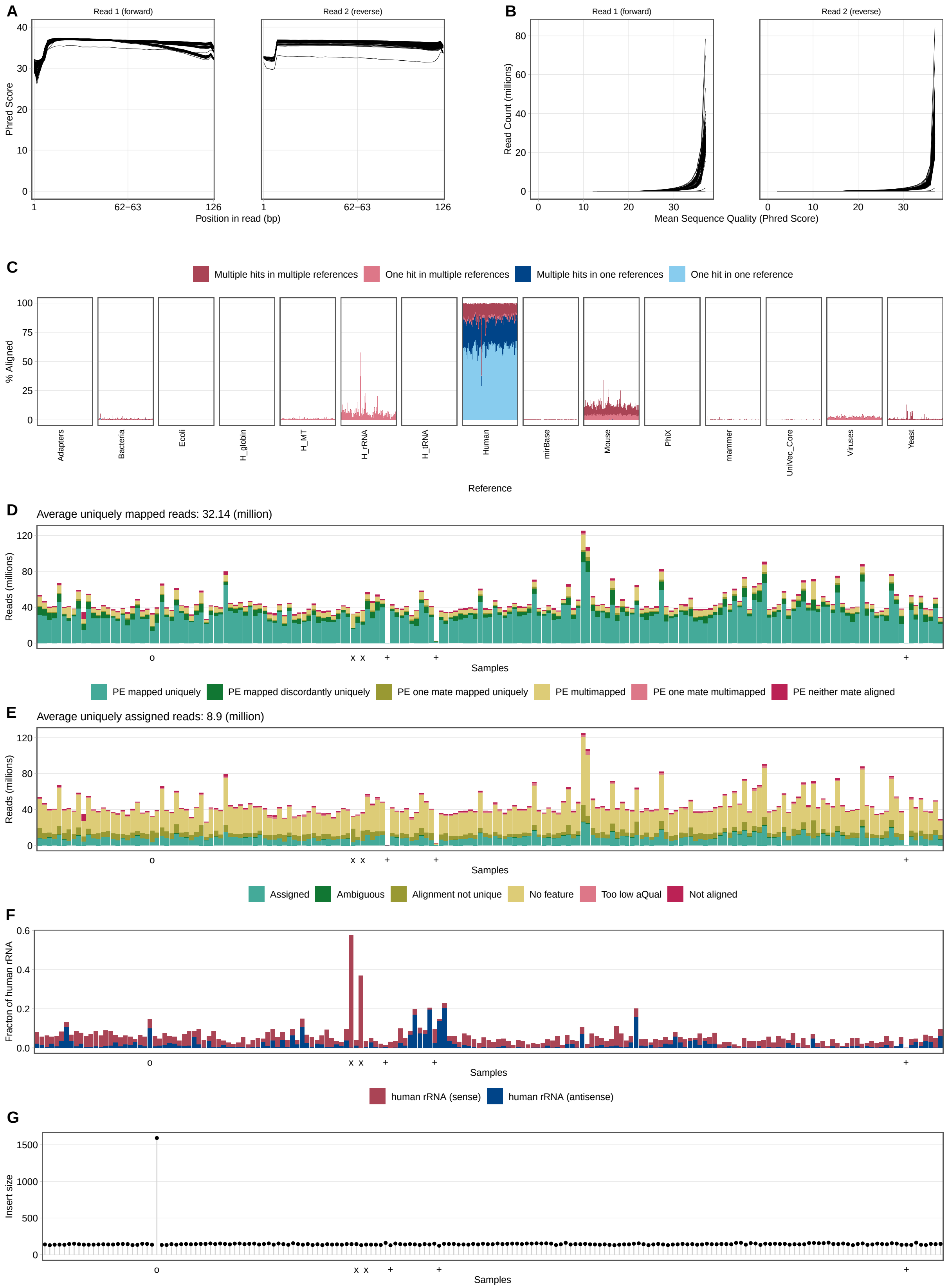
A
Read 1 (forward)
Read 2 (reverse)
Phred Score
Position in read (bp)
B
Read 1 (forward)
Read 2 (reverse)
Read Count (millions)
Mean Sequence Quality (Phred Score)
C
Multiple hits in multiple references
One hit in multiple references
Multiple hits in one references
One hit in one reference
% Aligned
Adapters
Bacteria
Ecoli
H_globin
H_MT
H_rRNA
H_tRNA
Human
mirBase
Mouse
PhiX
rnammer
UniVec_Core
Viruses
Yeast
Reference
D
Average uniquely mapped reads: 32.14 (million)
Reads (millions)
Samples
PE mapped uniquely
PE mapped discordantly uniquely
PE one mate mapped uniquely
PE multimapped
PE one mate multimapped
PE neither mate aligned
E
Average uniquely assigned reads: 8.9 (million)
Reads (millions)
Samples
Assigned
Ambiguous
Alignment not unique
No feature
Too low aQual
Not aligned
F
Fracton of human rRNA
Samples
human rRNA (sense)
human rRNA (antisense)
G
Insert size
Samples

Figure S1: Quality Control of samples from the FFPE_Bx cohort with respect to sequencing library composition, read alignment. Following adapter trimming, each FASTQ file were assessed for average per base **(A)** and per sequence **(B)** quality as measured by Phred score. **(C)** To assess the sequencing library composition, each sample was subsampled to randomly 1 million trimmed paired-end reads. `FastQ Screen` in conjunction with `bowtie2` was conducted to detect possible contamination like for example bacteria and overrepresented fractions of RNA species like human rRNA. The y-axis depicts the percentage of first reads for each sample that aligned against the references from Table S4. Reads are classified into four distinct types indicating reads uniquely mapping in one sequence reference (one hit in one reference), reads with multiple mappings in one sequence reference (multiple hits in one reference), reads uniquely mapping in distinct sequence references (one hit in multiple references) and reads with multiple mappings in distinct sequence databases (multiple hits in multiple references). **(D)** Quality assessment of read alignment results for each sample. Mapped uniquely: read pairs aligned concordant 1 time. Multi-mapped: read pairs aligned concordant >1 times. Mapped discordantly uniquely: read pairs aligned discordantly 1 time. One mate mapped uniquely: one read of the pair maps to the genome 1 time. One mate multi-mapped: one read of the pair maps to the genome >1 times. **(E)** Results of gene quantification for each sample. Quantification was performed with `HTSeq`. **(F)** Shown is the fraction of subsampled reads mapped against human rRNA transcripts. Reads are divided into sense, which resembles endogenous rRNA and antisense rRNA which resembles rRNA antisense probes from the rRNA depletion step. **(G)** Median insert size was estimated by Qualimap- For **(D-G)**: Samples mapped with at least 25% against the human rRNA reference depicted with a "x" symbol. Samples with a low sequencing depth of less than 5 million are represented by a "+" symbol, samples with an insert size of more than 1000 bp are represented by a "o" symbol.

Table S4: References used for FastQ Screen

Reference	Source
Adapter sequences	https://github.com/csf-ngs/fastqc/blob/master/Contaminants/contaminant_list.txt
Bacteria	ftp://ftp.ncbi.nih.gov/genomes/refseq/bacteria/, Oct 2014
H_MT	human mitochondrial reference sequence from GRCh37/hg38
H_rRNA (human ribosomal RNA sequences)	NR_003286.1 (18S), NT_003287.1 (28S), NR_003285.2 (5.8S), V00589.1 (5S), NC_012920.1: gi|251831106:1671-3229 (MT 16S) and NC_012920.1: gi|251831106:648-1601 (MT 12S)
H_tRNA (human transfer RNA sequences)	http://gtrnadb.ucsc.edu/genomes/eukaryota/Hsapi38/hg38-tRNAs.fa
Human genome	GRCh38/hg38, reference chromosomes only
mirBase	miRNA sequences from mirBase v21
Mouse genome	UCSC/mm10
PhiX	gi|9626q372|ref|NC_001422.1| Enterobacteria phage phiX174 sensu lato, complete genome
RNAmmer (predicted rRNA sequences)	http://www.cbs.dtu.dk/services/RNAmmer/, v1.2
UniVec Core	ftp://ftp.ncbi.nlm.nih.gov/pub/UniVec/UniVec_Core build 8.0, May 2015
Viruses	ftp://ftp.ncbi.nlm.nih.gov/genomes/refseq/viral/, March 2014
Yeast	Genome assembly SacCer3

2.2 External cohorts

Microarray datasets: Raw .CEL files from Affymetrix microarray datasets including CPC_GENE_2017_Fraser (GSE84042), MSKCC_2010_Taylor (GSE21034) and CancerMap_2017_Luca (GSE94767) were downloaded from NBCI's Gene Expression Omnibus (GEO) [18] using the `R` package `GEOquery` v2.58.0 [19]. Normalization and probeset summarization was performed applying the robust multi-array average algorithm (RMA) implemented in the `rma()` function of the `R`package `oligo` [20]. Updated probeset annotation chip definition files (CDF) based on GENCODE v36 and provided

by BrainArray [21] (version 25.0.0) were used to annotate corresponding array platforms (CDF files available at `http://brainarray.mbni.med.umich.edu/Brainarray/Database/CustomCDF/25.0.0/gencodeg.asp`). As suggested by Fraser et al [22], we correct batch effects between different arrays for the cohort CPC_GENE_2017_Fraser. For this, we applied the `ComBat()` function implemented in the the `sva` package v3.38 [23] to correct for batch effects between different arrays. The same strategy was performed for the cohort CancerMap_2017_Luca. For the array platforms of the cohorts CamCap_2016_Ross_Adams (GSE70768), Stockholm_2016_Ross_Adams (GSE70769) and Belfast_2018_Jain (GSE116918) the CDF files are not available. The processed array data were obtained using `GEOquery`. The gene IDs of the processed data that were annotated with gene symbols or Entrez IDs were converted to Ensembl IDs using the GENCODE v36 annotation. Expression values were averaged amongst duplicated Ensembl Gene IDs.

RNA-Seq datasets: Processed RNA-Seq data (FPKM normalized) from the cohort "DKFZ_2018_Gerhauser" were downloaded from cBioPortal [24]. The RPKM normalized counts from the Chinese Postate Cancer Genome and Epigenome Atlas (CPGEA_2020_Li) were downloaded from `http://www.cpgea.com` [25]. FPKM and RPKM values were transformed to log-space and quantile normalized. Raw sequencing data in FASTQ format from the RNA-Seq study "Atlanta_2014_Long" (GSE54460) were obtained using the `prefetch` and `fastq-dump` commands implemented in the package `SRA Toolkit` v2.9.2 (`https://github.com/ncbi/sratoolkit`). The processing steps for the FASTQ reads using the workflow-manager `uap` include the following: Reads were trimmed and filtered using `AdaptorRemoval` v.2.2.1a. Trimmed reads were mapped to the human reference genome (GRCh38/hg38) using `HISAT2` v2.1.0. Gene level quantification for the human reference gene annotation GENCODE (release 36 GRCh38.p12) was obtained using `HTSeq` v0.11.2. Gene counts were adjusted for library size and normalized with the variance-stabilizing transformation (vst) as implemented in DESeq2 v1.30.1. The pre-processing steps of the "TCGA_PRAD" cohort are described in Kreuz et al [7].

Principal component analysis of samples from all cohorts analyzed by microarrays and RNA-Seq revealed no critical outliers (Figure S2).

3 Confounding factors and gene filtering (FFPE_Bx)

3.1 Impact of reduced sequencing depth

To enable higher throughput, multiplexing was increased for the sequencing of FFPE_Bx compared to previous training cohorts [7]. Therefore, we expect a reduction in the accuracy of the measurement especially for genes with low expression levels. This might have a negative impact for the prognostic accuracy of ProstaTrend. Investigation of the expression levels of ProstaTrend genes showed average/high expression for the majority of genes (median expression level $>$25 raw reads for 80% of the ProstaTrend genes; Figure S3A). Exclusion of genes with lower expression levels (median(reads) $<$100; 836 genes remaining) did not improve the performance of Transcriptomic

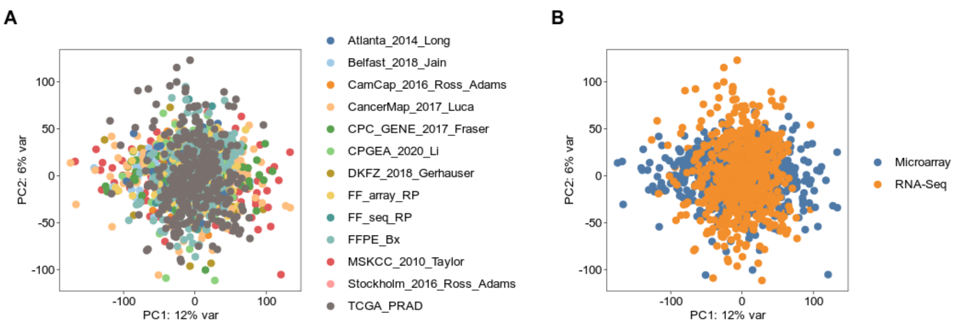

Figure S2: Principal component analysis of all tissue specimens, either assessed by transcriptome-wide next-generation sequencing or by microarrays after standardization of normalized expression values. We conducted principal component analysis for genes covered by all cohorts. The plot depicts the first and second principal components. Sample are colored by study **(A)** and platform **(B)**.

Risk Score (TRS) (log-rank test: p-value = 0.49; Figure S3B) indicating that higher multiplexing alone does not explain the significantly lower prognostic significance of the TRS) in the FFPE_Bx cohort.

3.2 Impact of tumor cell content

Given the larger variety of tumor cell content for the specimens of the FFPE_Bx cohort, we hypothesized that samples with low tumor cell content cannot be evaluated with ProstaTrend as relevant expression signals are masked by RNA patterns from non-tumor cells. We tested this hypothesis in two different ways. First, we excluded specimens with low tumor cell content (<40%) from the analysis and investigated the prognostic role of ProstaTrend in this high tumor cell content subgroup. Application of the ProstaTrend TRS to this high tumor cell content subgroup did not result in a significant prognostic association of the TRS (log-rank test: p-value = 0.3; Figure S3C). Even taking into account the smaller sample size due to the filtering, there was quantitatively only a small prognostic difference associated with TRS.

In a second approach, we analyzed the relationship of the expression of individual genes with the tumor cell content. We applied linear regression analysis to investigate the impact of tumor cell content on the expression level for all ProstaTrend genes that passed the expression filter. Genes that showed a trend toward association of tumor cell content and expression levels (p<0.1) were filtered and we calculated TRS only without these genes (n=663 genes). Filtering of genes associated with tumor cell content did not improve the prognostic association of the TRS in the FFPE_Bx cohort (log-rank test: p-value = 0.18; Figure S3D). So overall, filtering for genes associated with tumor cell content or filtering of samples with low tumor cell content did not lead to a relevant improvement of the prognostic accuracy of the ProstaTrend TRS.

3.3 Impact of FFPE specimen age

We hypothesized that a systematic impact of degradation over time on the expression levels for some ProstaTrend genes might deter the prognostic association of ProstaTrend in FFPE cohorts. Thus, we analyzed the relationship of the expression of individual genes with the specimen age to identify genes that might be especially prone to FFPE associated degradation effects. Using linear regression analysis, we investigated the impact of specimen age on the expression level for all ProstaTrend genes that passed the expression filter. Overall, a strong enrichment of genes with significant association of specimen age and expression levels is observed (Figure S3E). Filtering of genes that showed a trend toward association of specimen age and expression levels (p-value <0.1) resulted a set of n=541 genes without indication for specific FFPE associated degradation effects. Calculation of TRS with these remaining genes resulted in a strong prognostic association (Figure S3F; log-rank test: p-value $=0.00021$). This provides strong evidence that a large proportion of genes affected by strong FFPE associated degradation mask the prognostic significance of ProstaTrend.

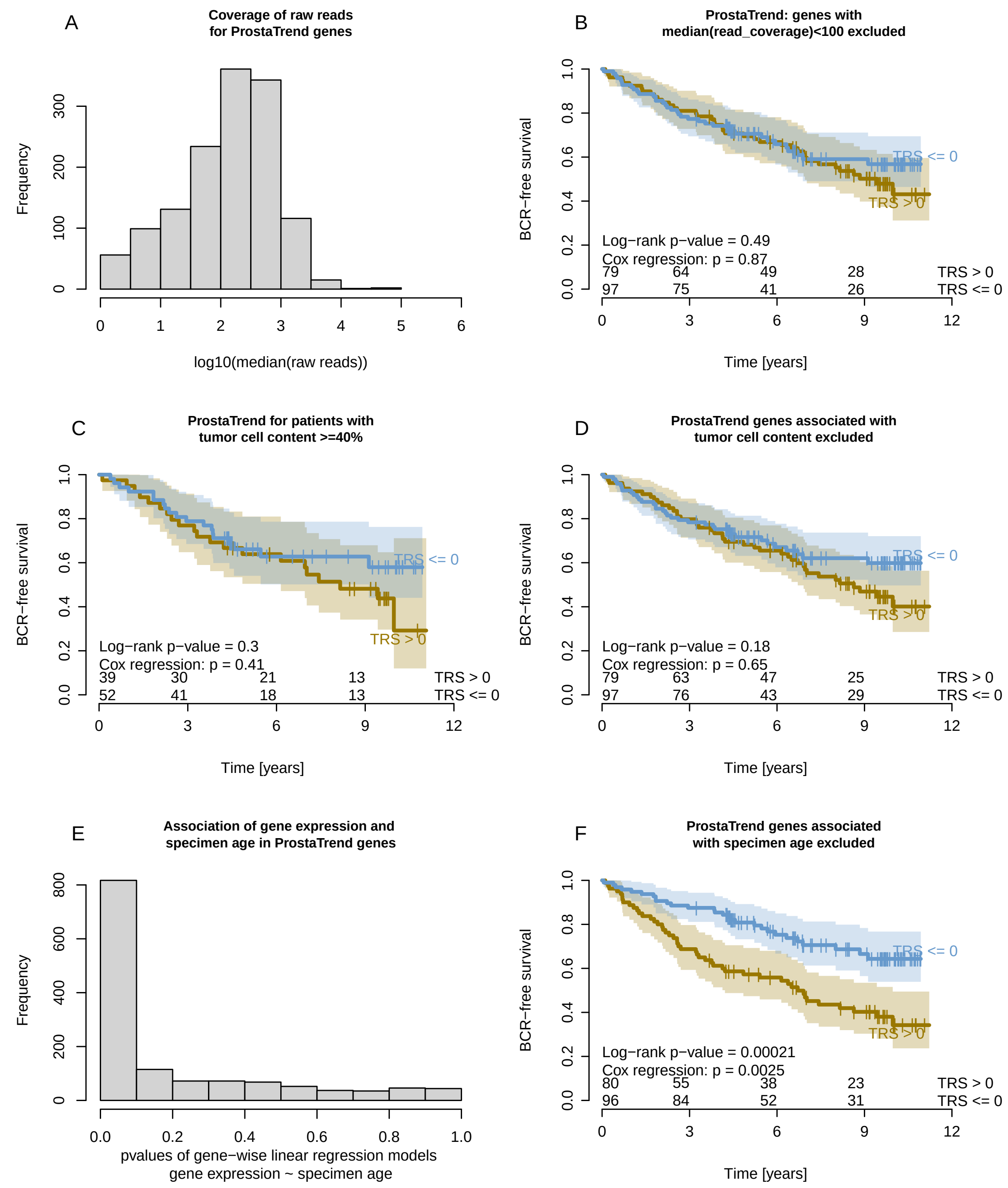

Figure S3: Filtering of ProstaTrend for genes that may impair reproducibility of the Transcriptomic Risk Score (TRS) in the UKPD cohort. (A) Histogram of median read counts for genes included in the TRS. **(B)** Kaplan-Meier analysis for ProstaTrend TRS after filtering for genes with lower expression levels (median(raw reads) $<$100). **(C)** Kaplan-Meier curve for TRS applied to samples of the FFPE_Bx cohort with tumor cell content $\geq$ 40%. **(D)** Kaplan-Meier curve of the TRS after filtering genes that show an association of tumor cell content and gene-expression (linear regression p $<$0.1). **(E)** Histogram of the p-values for linear regression analysis of $gene - expresson \sim specimenage$ for all n=1396 ProstaTrend genes. **(F)** Kaplan-Meier analysis of the TRS after filtering genes that show an association of specimen age and gene-expression (linear regression p $<$0.1). For **(B, C, D, F)** Grey shades depict the 95%-CI for Kaplan-Meier curves. Time to biochemical relapse is compared between patients with TRS $>$0 and TRS $\leq$ 0 by log rank test. In addition, a univariate Cox-regression analysis for TRS on a continuous scale was performed

A

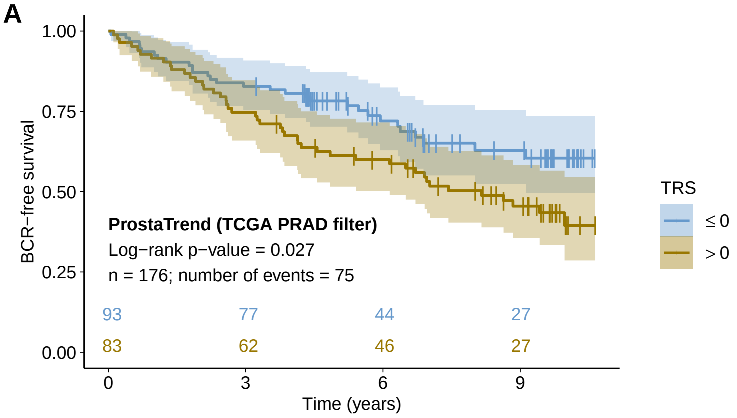

B

	logHR [95% CI]	HR	p−value
ProstaTrend score	1.20 [0.25, 2.15]	3.32	0.013

	logHR [95% CI]	HR	p−value
ProstaTrend score	0.28 [−0.69, 1.26]	1.33	0.566
Biopsy GGG > 2	1.20 [0.69, 1.70]	3.31	3.31e−06

Figure S4: Filtering of ProstaTrend for genes using TCGA_PRAD. Prognostic value of ProstaTrend restricted to genes having a consistent prognostic effect between the training cohorts and TCGA PRAD (univariate Cox-regression with p-value <0.1 in TCGA PRAD and consistent log hazard ratios). Overall 517 genes retained. **(A)** Kaplan-Meier curves for patients with TRS >0 (increased risk) compared to patients with TRS ≤0 (reduced risk). Color shades depict the 95% confidence intervals for Kaplan-Meier curves. The curves were truncated if the number of patients at risk dropped below 10 in both groups. The colored numbers above the x-axis indicate the number of patients at risk. Log-rank tests were performed to evaluate probabilities of BCR-free survival between these two groups. The numbers under the log-rank p-values indicate the number of patients and cases with BCR. **(B)** Univariate Cox-regression analysis for TRS on a continuous scale (top) and multivariable Cox-regression for TRS adjusted for Gleason grading group >2 of the biopsies (bottom). logHR = log hazard ratio, HR = hazard ratio, CI = confidence interval.

4 Prognostic value of ProstaTrend in validation cohorts

4.1 Survival analysis of ProstaTrend-ffpe using TRS.

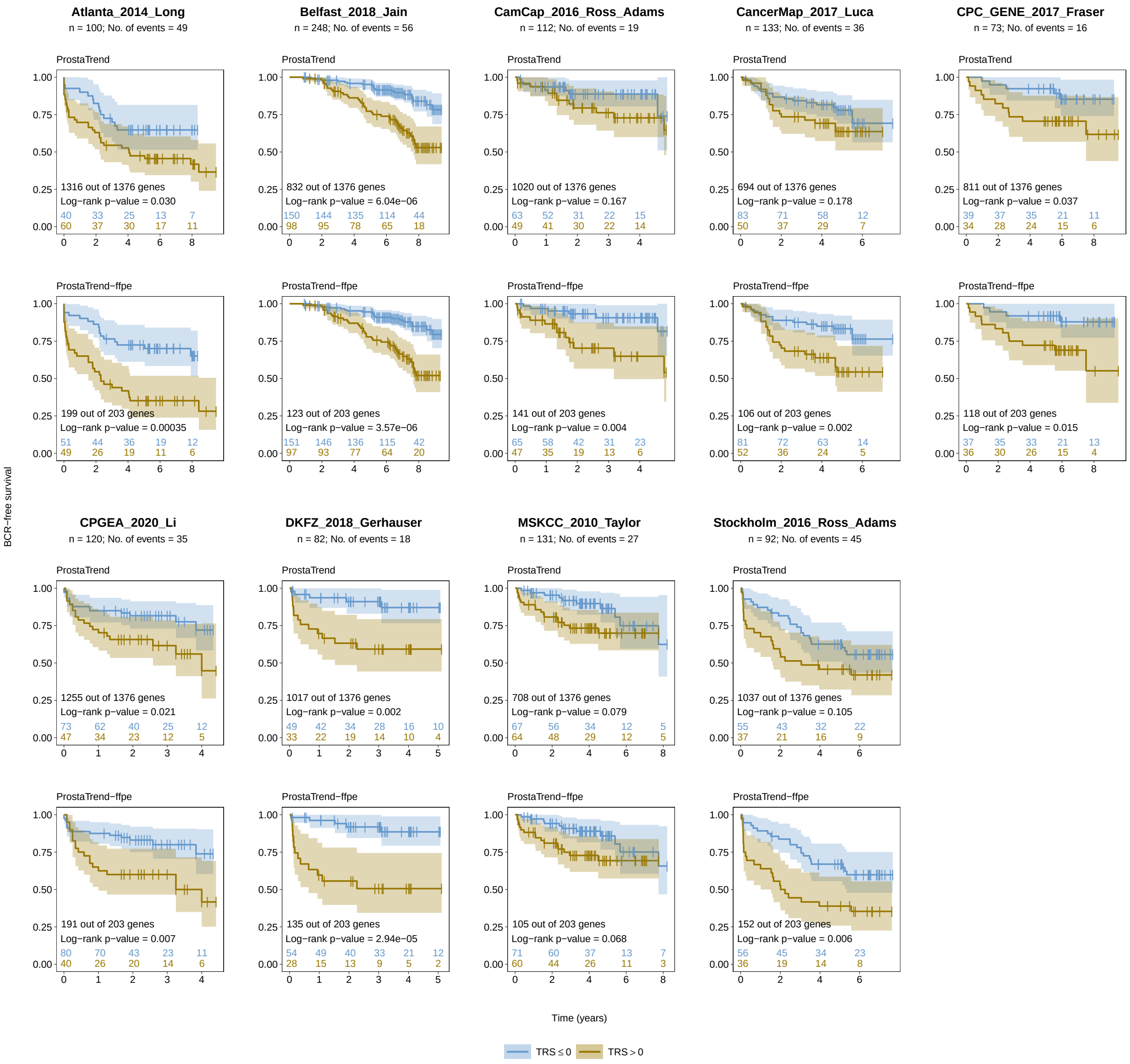

Figure S5: Kaplan-Meier analysis of the ProstaTrend-ffpe Transcriptomic Risk Score (BCR as the primary endpoint). To validate TRS using the ProstaTrend-ffpe signatures in cohorts, we performed Kaplan-Meier analysis in 9 publicly available cohorts. The numbers under the cohort IDs indicate the number of patients and cases with BCR. Kaplan-Meier curves for patients with TRS score >0 (increased risk) compared to patients with TRS ≤ 0 (reduced risk). Color shades depict the 95%-CI for Kaplan-Meier curves. The curves were truncated if the number of patients at risk dropped below 10 in both groups. The colored numbers above the x-axis indicate the number of patients at risk. Log-rank tests were performed to evaluate probabilities of BCR-free survival between these two groups. The numbers in the diagram (above the p-values) indicate how many ProstaTrend and ProstaTrend-ffpe genes are available in the corresponding datasets.

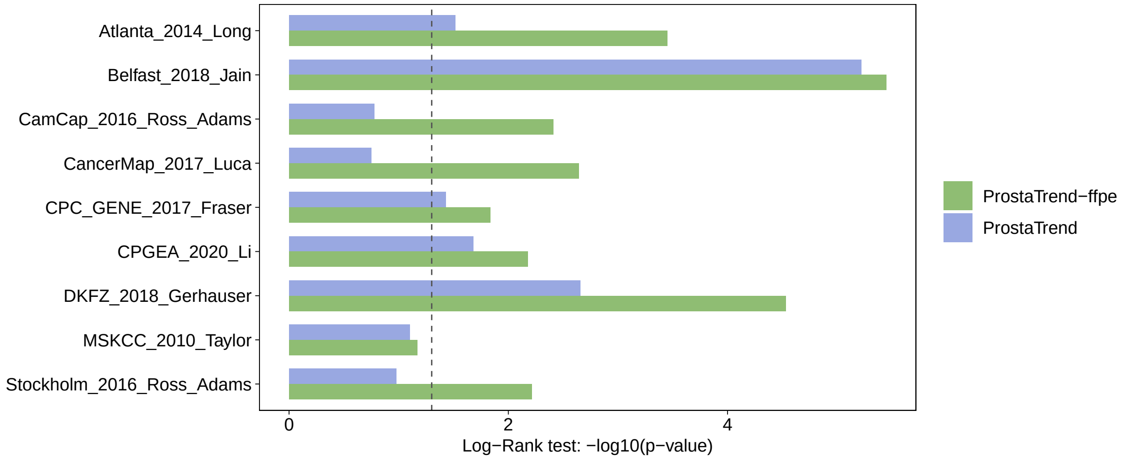

Figure S6: Prognostic value of Transcriptomic Risk Score using the ProstaTrend-ffpe signatures by log-rank test. A log-rank test was performed to assess the probability of BCR-free survival of patients with TRS > 0 (increased risk) compared with patients with TRS ≤ 0 (reduced risk). The p-values are the same as in Figure S5, except that they have been converted to logarithmic space (-log10(p-value)). The vertical line corresponds to a p-value of 0.05.

4.2 TRS for primary tumor and matched healthy tissue

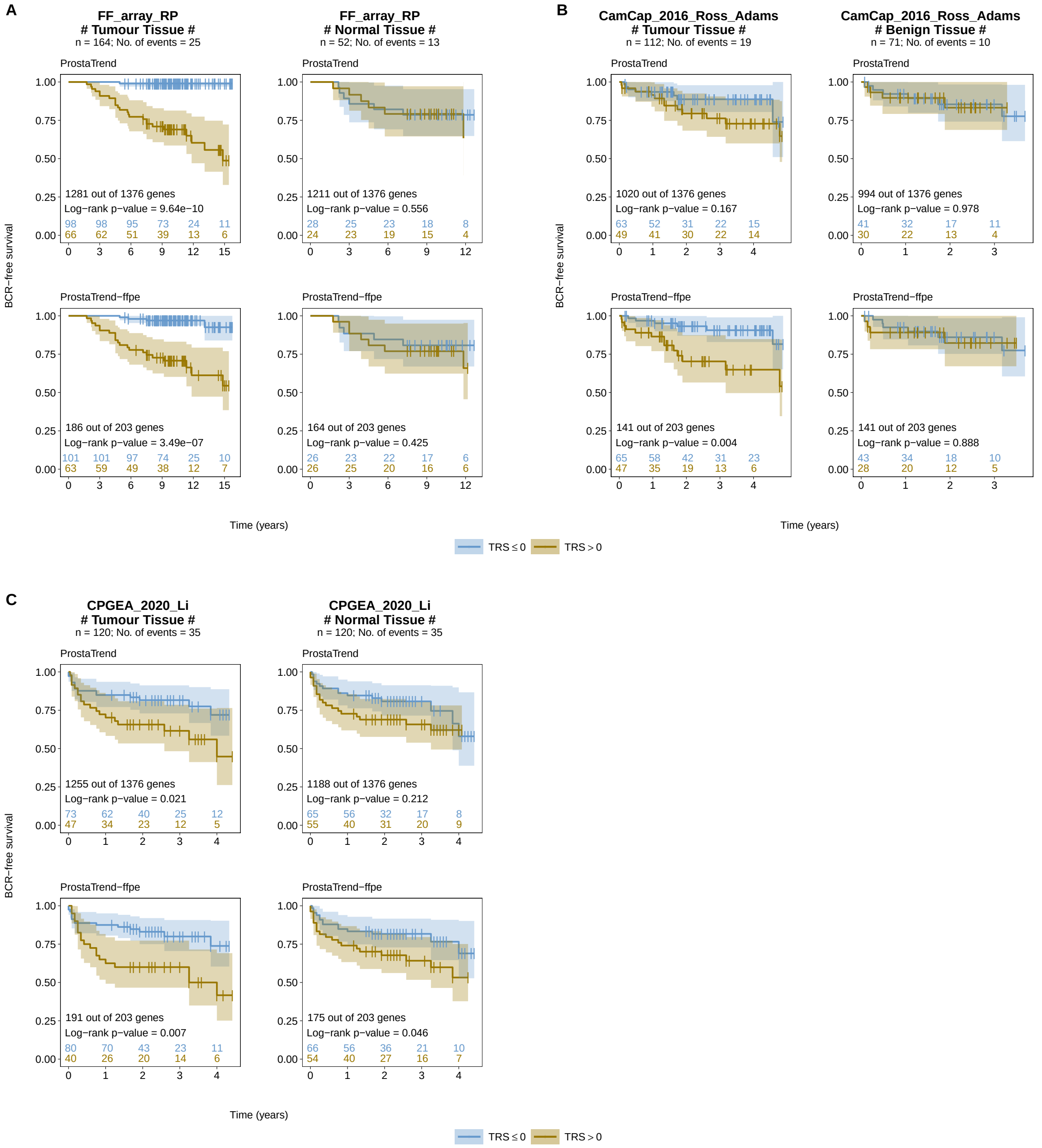

Figure S7: Kaplan-Meier analysis of the ProstaTrend-ffpe Transcriptomic Risk Score in 3 cohorts with primary tumor and matched benign/healthy tissue. Matched benign/healthy biopsies with at least 10 events are available for three cohorts, one from the ProstaTrend training cohorts **(A, right panel)** and two from the validation cohorts **(B and C, right panel)**. Kaplan-Meier curves for patients with TRS > 0 (increased risk) compared to patients with TRS $\leq$ (reduced risk). Color shades depict the 95%-CI for Kaplan-Meier curves. The curves were truncated if the number of patients at risk dropped below 10 in both groups. The colored numbers above the x-axis indicate the number of patients at risk. Log-rank tests were performed to evaluate probabilities of BCR-free survival between these two groups. The numbers under the log-rank p-values indicate the number of patients and cases with BCR.

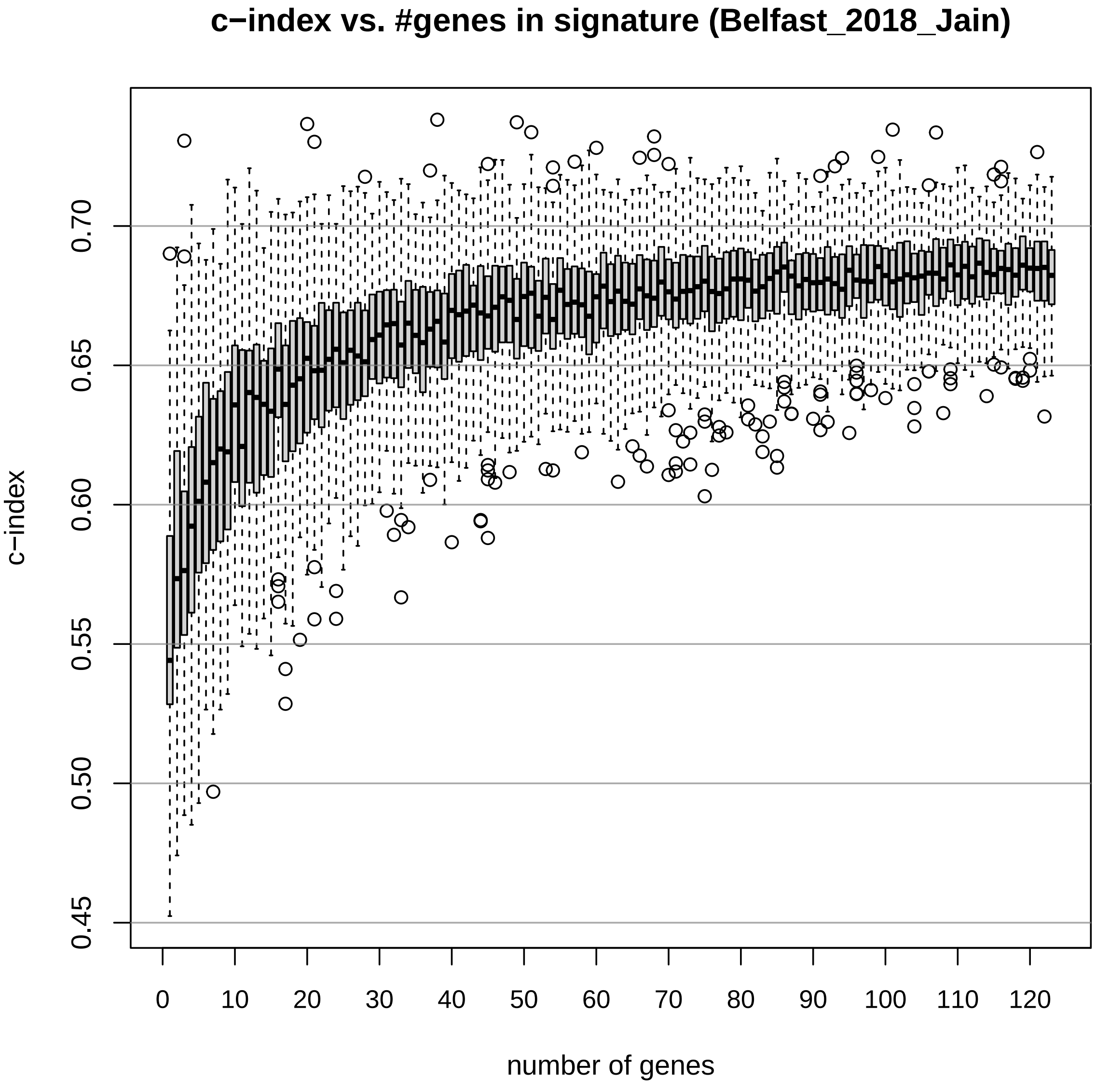

Figure S8: Comparison of concordance index (c-index) in relationship to signature size. For the Belfast_2018_Jain cohort 100 bootstrap samples for the signature genes were selected with replacement for each analyzed signature size. The size of the bootstrap samples were varied between 1 and 123 genes (overlap of ProstaTrend-ffpe with the genes measured in the cohort). For each bootstrap sample the TRS was calculated with the selected gene subset and the c-index was determined. Boxplots highlight the c-index distribution of a given signature size.

4.3 Multivariate Cox proportional hazard model

Study	Covariates	LogHR [95% CI]	P-value
Atlanta_2014_Long	ProstaTrend-ffpe TRS	0.297[-0.037, 0.631]	0.081
	Gleason score	0.511[0.083, 0.939]	0.019
Belfast_2018_Jain	ProstaTrend-ffpe TRS	0.742[0.475, 1.009]	5.24e-08
	Gleason score	-0.133[-0.413, 0.148]	0.354
CamCap_2016_Ross_Adams	ProstaTrend-ffpe TRS	0.902[0.468, 1.336]	4.67e-05
	Gleason score	1.036[0.072, 2.001]	0.035
CancerMap_2017_Luca	ProstaTrend-ffpe TRS	0.391[0.071, 0.711]	0.017
	Gleason score	0.452[-0.031, 0.934]	0.067
CPC_GENE_2017_Fraser	ProstaTrend-ffpe TRS	0.546[0.016, 1.075]	0.043
	Gleason score	0.277[-1.29, 1.845]	0.729
CPGEA_2020_Li	ProstaTrend-ffpe TRS	0.392[0.086, 0.697]	0.012
	Gleason score	0.329[-0.044, 0.702]	0.083
DKFZ_2018_Gerhauser	ProstaTrend-ffpe TRS	0.88[0.42, 1.34]	0.00018
	Gleason score	0.317[-0.212, 0.845]	0.240
MSKCC_2010_Taylor	ProstaTrend-ffpe TRS	0.3[-0.071, 0.672]	0.113
	Gleason score	1.158[0.713, 1.603]	3.42e-07
Stockholm_2016_Ross_Adams	ProstaTrend-ffpe TRS	0.427[0.122, 0.733]	0.006
	Gleason score	0.704[0.374, 1.035]	2.97e-05

Table S5: Multivariate Cox proportional hazard regression for all validation cohorts. The standardized TRS using the ProstaTrend-ffpe signature was adjusted for the Gleason score on a continuous scale and fitted for time to BCR

4.4 Association between the TRS and clinicopathological parameters

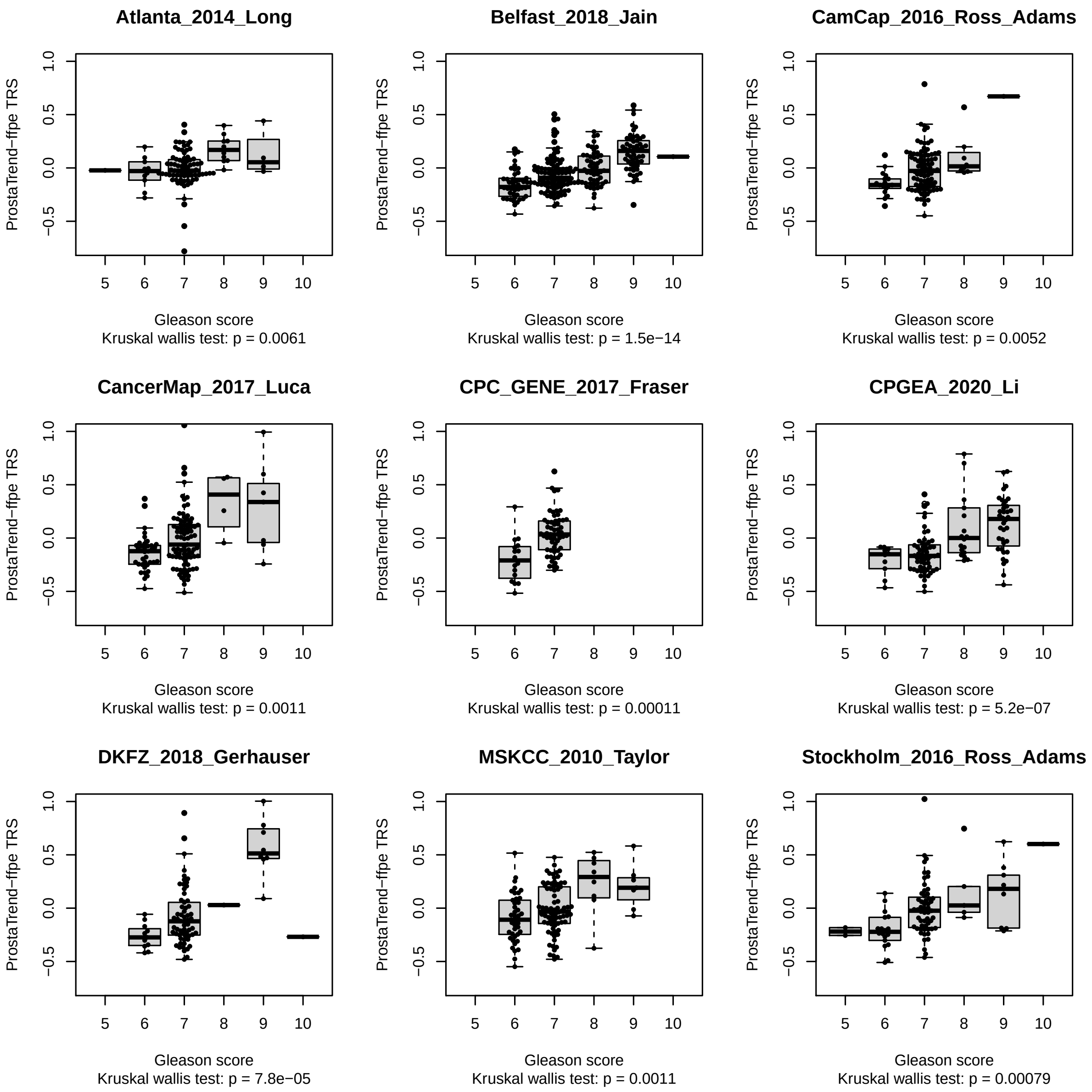

Figure S9: Association between the ProstaTrend-ffpe TRS with Gleason score. Differences in TRS between Gleason scores were assessed using the Kruskal-Wallis test.

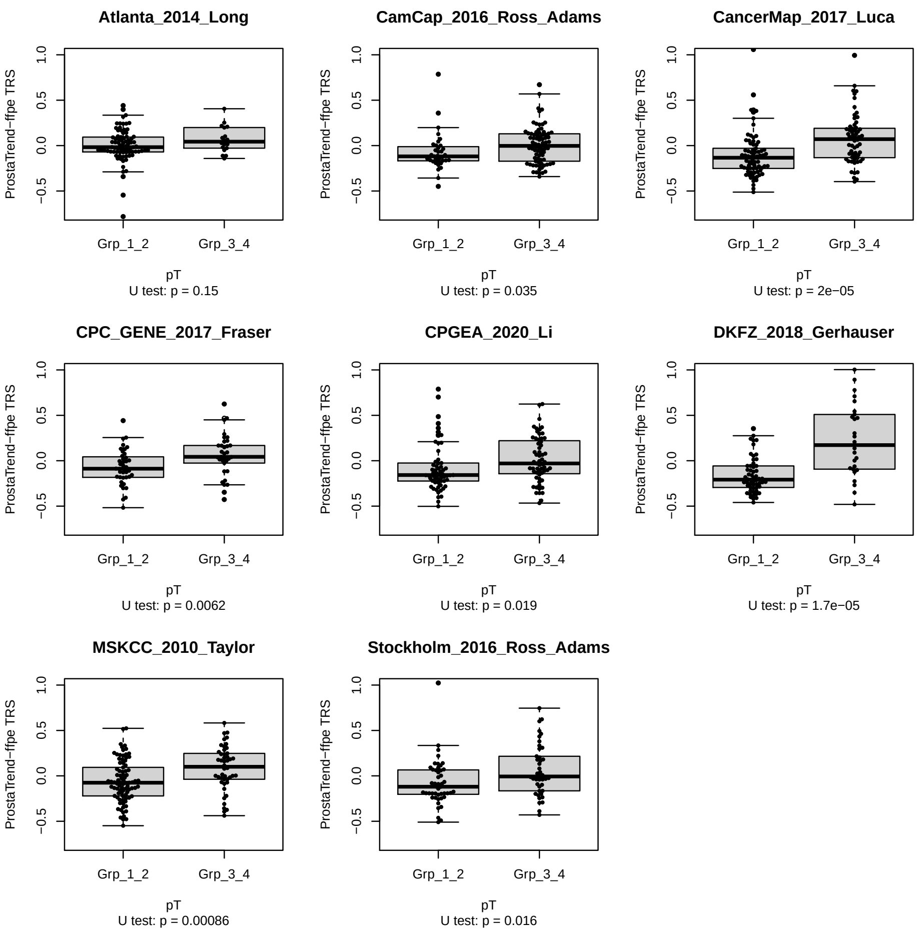

Figure S10: Association between the ProstaTrend-ffpe TRS with pathological T stage. Differences in TRS between low (T1 and T2) and high (T3 and T4) T stage groups were assessed using the Mann-Whitney U test.

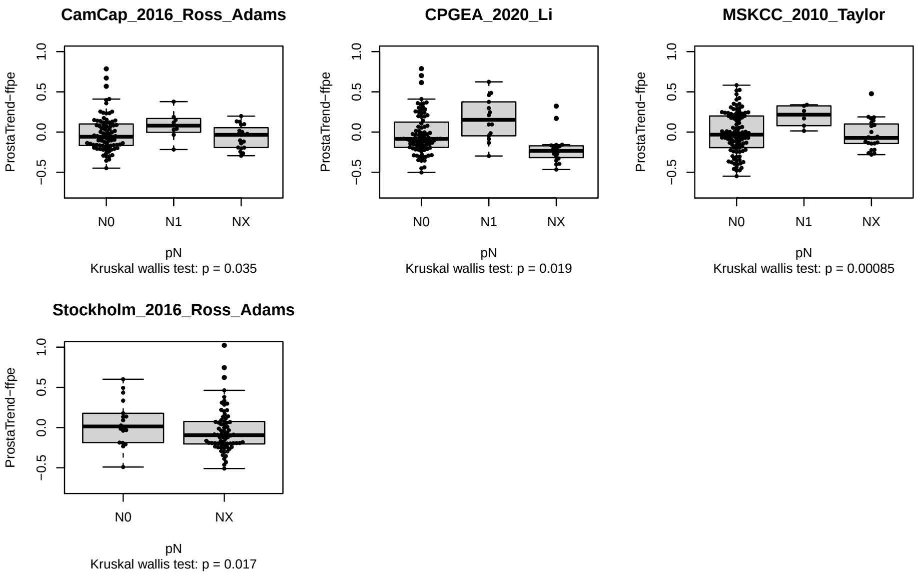

Figure S11: Association between the ProstaTrend-ffpe TRS with lymph node staging. Differences in TRS between node staging groups were assessed using the Kruskal-Wallis test. pNX = Cannot be assessed.

4.5 Published gene expression-based signatures for PCa prognosis

Signature	No..of.genes	PMID	Journal	Year	Included	Exclusion.criteria
Agell	12	23083832	The American journal of pathology	2013	yes	-
Bibikova	16	17459658	Genomics	2007	yes	-
Cuzick (Prolaris)	31	21310658	The Lancet. Oncology	2011	yes	-
Ding	4	21289624	Nature	2011	yes	-
Erho (Decipher)	19	23826159	PloS one	2014	yes	-
Irshad	19	24027026	Science translational medicine	2014	yes	-
Kamoun	36	29945238	Annals of oncology	2019	yes	-
Klein (Oncotype DX)	12	24836057	European urology	2015	yes	-
Long	24	24713434	Cancer research	2014	yes	-
Luca	45	28753852	European urology focus	2019	yes	-
Nakagawa	17	18846227	PloS one	2008	yes	-
Ramos-Montoya	222	24737870	EMBO molecular medicine	2014	yes	-
Sinnott	30	27663590	Clinical cancer research	2018	yes	-
Talantov	3	20723930	The Journal of urology	2010	yes	-
Varambally	44	16286247	Cancer cell	2006	yes	-
Yang	28	29729848	EBioMedicine	2018	yes	-
Yu	14	18006806	Cancer research	2007	yes	-
Bismar	12	16533427	Neoplasia (New York, N.Y.)	2006	no	comparison of multiple groups
Glinsky	11	15931389	The Journal of clinical investigation	2005	no	not designed for gene expression in human PCa tumor tissue
Jia	15	22870216	PloS one	2013	no	not designed for gene expression in human PCa tumor tissue
Li	160	32898860	Briefings in bioinformatics	2021	no	direction of the prognostic effect not available
Mo	93	28330676	European urology	2019	no	not designed for gene expression in human PCa tumor tissue
Olmos	9	23059046	The Lancet. Oncology	2013	no	not designed for gene expression in human PCa tumor tissue
Penney	157	21537050	Journal of clinical oncology	2011	no	direction of the prognostic effect not available
Planche	36	21611158	PloS one	2011	no	not designed for gene expression in human PCa tumor tissue
Ramaswamy	17	12469122	Nature genetics	2003	no	not specific for PCa
Ross	6	23059047	The Lancet. Oncology	2013	no	not designed for gene expression in human PCa tumor tissue
Ross-Adams	100	26501111	EBioMedicine	2016	no	comparison of multiple groups
Sharma	16	23260764	Cancer cell	2013	no	not designed for gene expression in human PCa tumor tissue
Wu	29	23533275	PNAS	2013	no	direction of the prognostic effect not available

Table S6: Prognostic PCa gene signatures. Gene expression-based published signatures for PCa prognosis. Gene sets are based on the study by Li et al. [26].

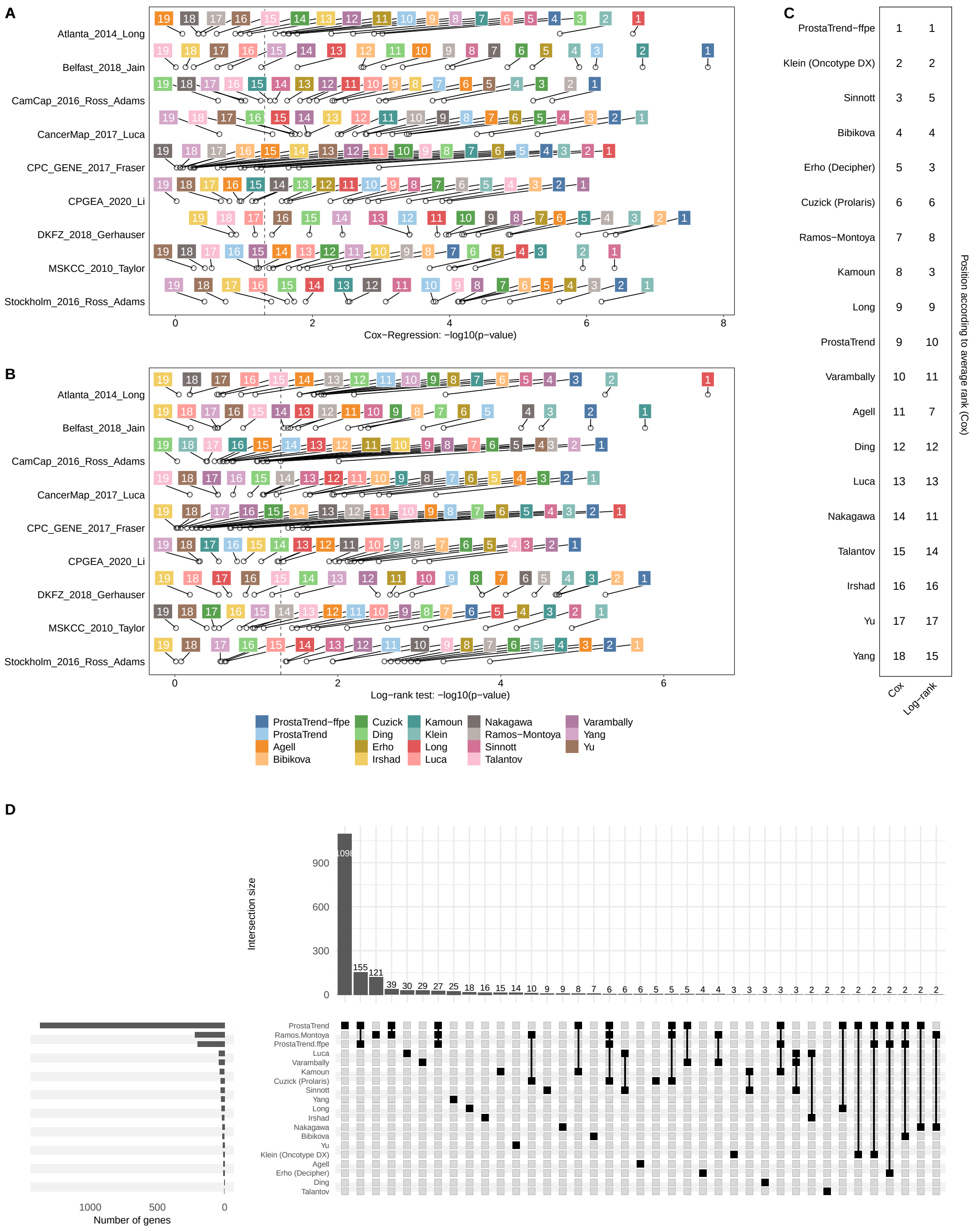

Figure S12: Comparison of the prognostic performance of ProstaTrend with other prognostically relevant PCa panels. (A) Using the cohort-wise standardized expression values of the validation cohorts, we applied for each prognostic prostate gene set (including both ProstaTrend gene sets) and patient a simplified Transcriptomic Risk Score which was the median of all genes at increased risk minus the median of genes at reduced risk. See Table S6 for an overview of the prognostic signatures. We then performed a **(A)** Log-rank and **(B)** Cox-regression analysis for each cohort to assess the prognostic performance of all gene sets. The dots depict the -log10(p-value) estimated from the log-rank test and Cox-regression model. The white numbers represent the rank by p-value. The dashed vertical line indicate a p-value of 0.05. **(C)** Average rank of each prognostic gene set across all cohorts from the log-rank and Cox-regression analysis. **(D)** UpSet plot of all analyzed prognostic prostate gene sets. The number of genes for the gene sets are indicated as horizontal bar plots. The squares in the matrix represent unique and overlapping genes for the gene sets. Connected squares indicate a certain intersection of genes between the gene sets. The bar graph above the matrix summarizes the number of DE genes for each unique or overlapping combination.

5 Meta-analysis

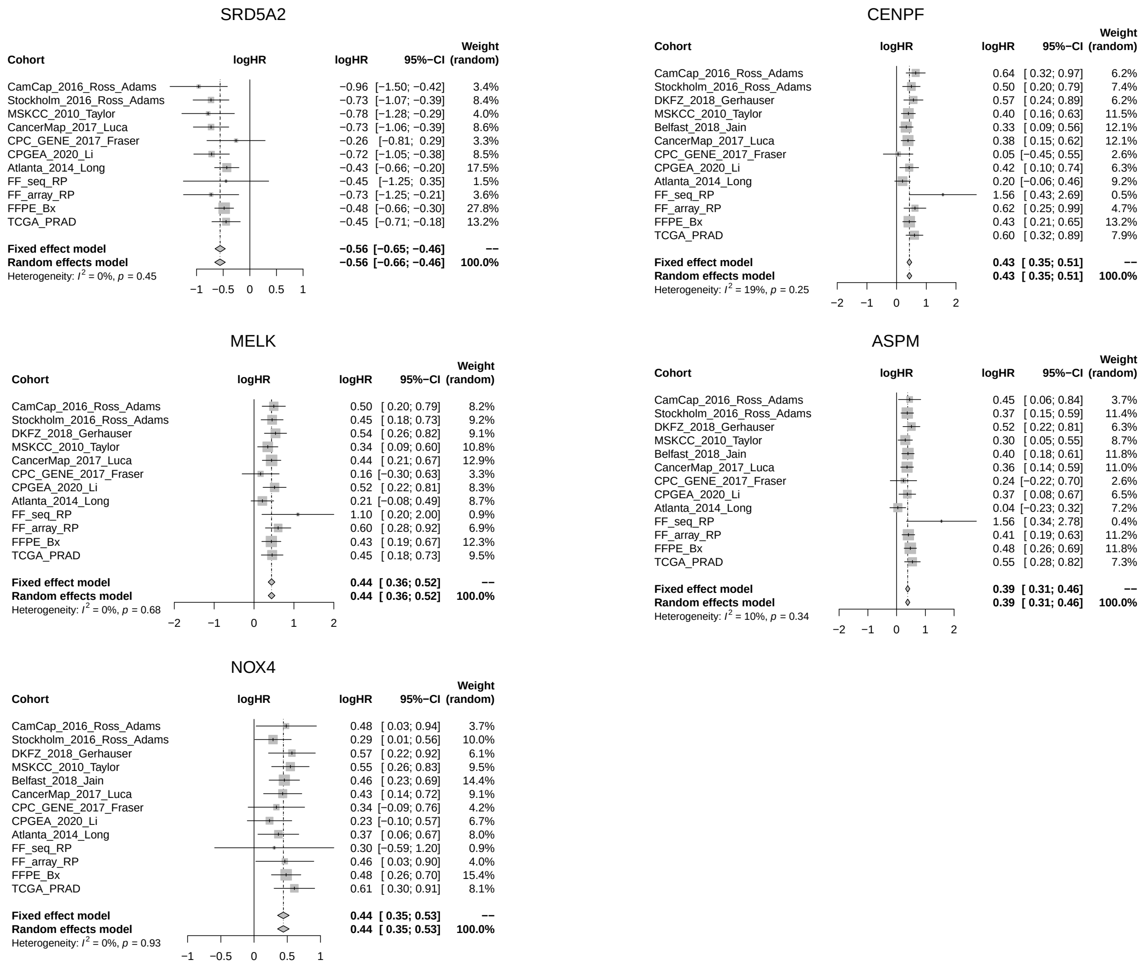

Figure S13: Forest plots of the 5 highest ranked genes from the meta-analysis using a univariate random-effect model. The plots represent a more detailed representation of Figure 5 from the main part. Forest plots for 5 highest ranked genes by adjusted p-value. The log hazard ratio for each cohort is represented by a square. The confidence interval (CI) is depicted by a horizontal line. The size of the square corresponds to the weight of the cohort in the meta-analysis. The combined effect and the CI is represented by a diamond. The between-study heterogeneity is depicted by the I^2 statistic. The p-value refers to the heterogeneity statistic Q.

Part II. Prostate cancer single-cell atlas

6 Pre-processing

Overall, we analyzed 79 prostate biopsy tissue samples from 41 patients. Figure S14 provide an overview of the datasets analyzed. For clinicopathological characteristics see Table S7. Low-quality cells were filtered out for the datasets from Dong et al. [27] and Song et al. [28] as suggested in the publications. The single-cell data sets from Chen et al. [29] and Ma et al. [30] were already prefiltered for low-quality cells. This was also partially true for the dataset of Tuong et al. [31]. In addition, we filtered out potential sperm cells from seminal fluid contamination, as suggested by the authors. We excluded one sample from the study by Chen et al. because it was a biopsy of a lymph node metastasis that cannot be directly compared with primary tumors. Figure S15 depicts quality control metrics of the analyzed samples, illustrating the heterogeneity of samples between and within studies in terms of the number of sequenced cells, median UMI counts and median genes present (with >0 UMI count) per sample. However, PCA analysis (Figure S16) based on the normalized values and the 2000 genes with the highest variance in all data sets suggests that the first two components explain the variance of the cell lineages and not of the different studies.

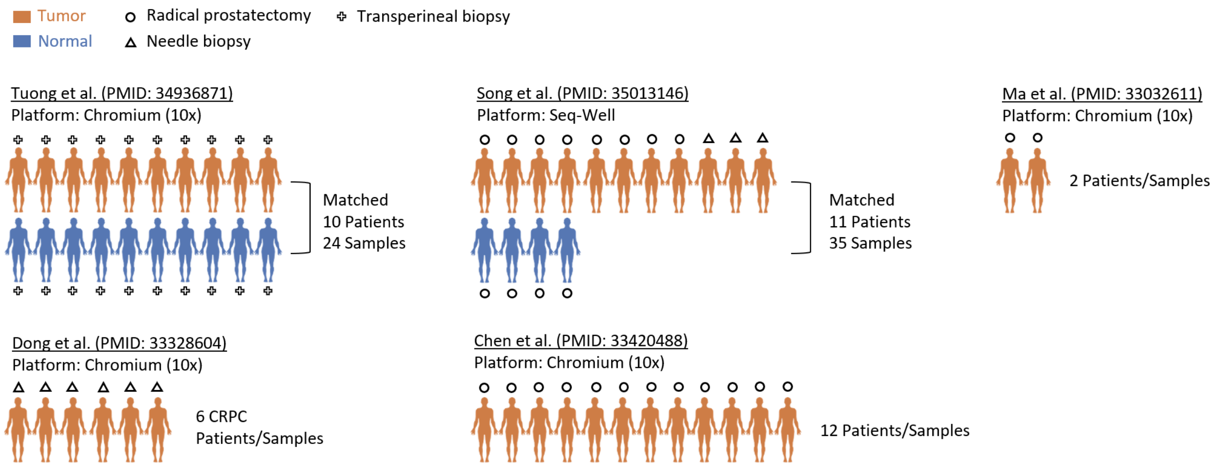

Figure S14: Overview of scRNA-Seq datasets used to develop the PCa cell atlas.

7 Analysis strategy

To account for differences in sequencing depth across cells, UMI counts were normalized by the total number of UMI's per cell, converted to transcripts per 10,000 and then natural log transformed using a pseudocount of 1. The datasets of all studies were then concatenated. Only genes that were available in all datasets were analyzed.

Characteristic	Chen et al. No. = 12	Dong et al. No. = 6	Ma et al. No. = 2	Song et al. No. = 11	Tuong et al. No. = 10
**Tissue source**					
Needle biopsy	0 (0%)	6 (100%)	0 (0%)	3 (27%)	0 (0%)
Radical prostatectomy	12 (100%)	0 (0%)	2 (100%)	8 (73%)	0 (0%)
Transperineal biopsy	0 (0%)	0 (0%)	0 (0%)	0 (0%)	10 (100%)
**Age (years)**					
Median (IQR)	74 (70, 78)	80 (72, 82)	NA (NA, NA)	NA (NA, NA)	68 (65, 71)
Not available	0	0	2	11	0
**Clinical stage**					
cT1/cT2	5	NA	NA	5	NA
cT3/cT4	6	NA	NA	2	NA
Not available	1	6	2	4	10
**Lymph node status**					
N0	9	NA	NA	NA	NA
N1	3	NA	NA	NA	NA
Not available	0	6	2	11	10
**Gleason score**					
6	0	0	1	1	3
7	4	2	1	9	6
8	0	2	0	0	0
9	7	1	0	1	1
10	1	0	0	0	0
small-cell NEPC	0	1	0	0	0
**PSA level (ng/mL)**					
<=10	1	NA	NA	9	7
>10 & <= 20	6	NA	NA	2	1
> 20	5	NA	NA	NA	2
Not available	0	6	2	0	0

Table S7: PCa cell atlas: Overview of the clinicopathological parameters. Shown are the characteristics from tumor specimens.

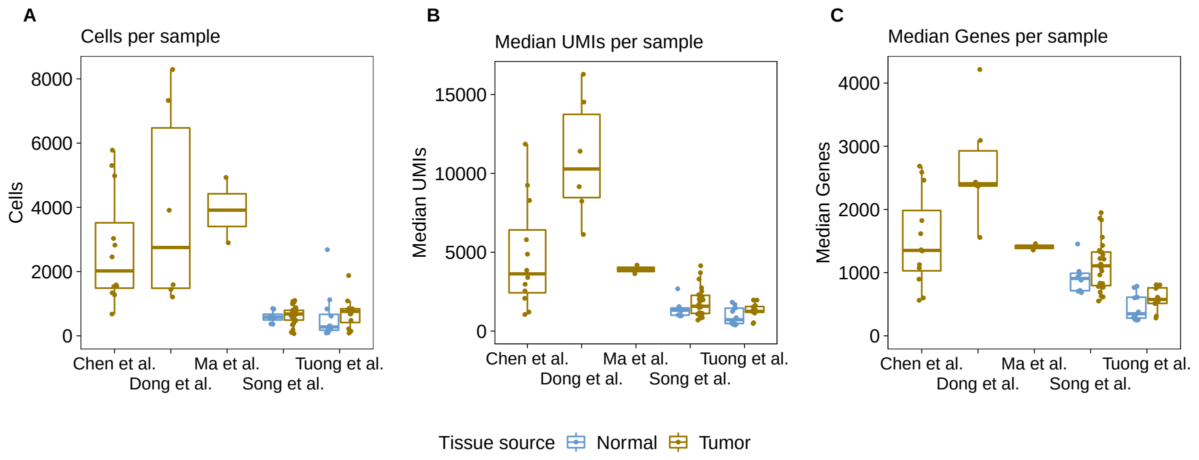

Figure S15: Metrics for quality control of analyzed samples. (A) The number of cells in each sample, grouped by study and colored by sample type. **(B)** The median number of UMI counts. **(C)** The median number of genes with at least one UMI count.

7.1 Integration and clustering

Integration and clustering analysis were performed using the `Seurat` v4.1.0 `R` package [32]. For each patient, the 2000 most variable genes were estimated by the "vst" method of the `FindVari-`

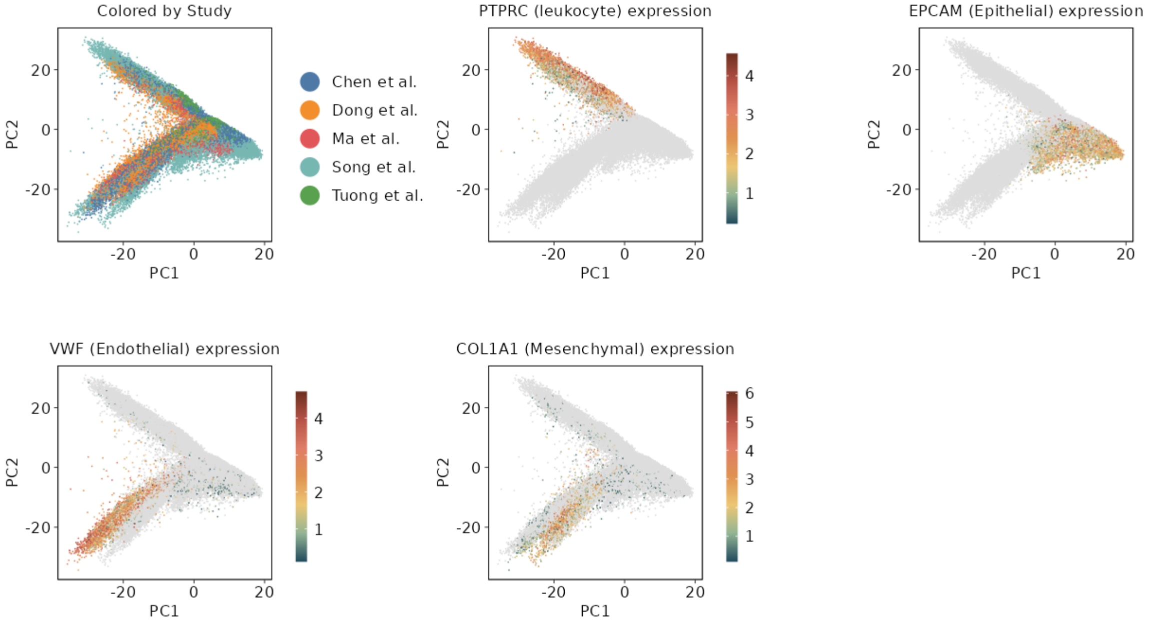

Figure S16: Principal component analysis (PCA). The diagram shows the first and second principal components. Cells are colored by study or by gene markers associated with leukocyte, epithelial, endothelial, and mesenchymal lineages. An increasing color gradient from blue to red corresponds to an increasing normalized expression level. Gray colored cells indicate no expression. The 2000 most variable genes were used for PCA.

`ableFeatures` function. We then used the `SelectIntegrationFeatures` function to select 2000 genes that were repeatedly shown to be highly variable in multiple patients. Thereafter, the data were standardized with `ScaleData`, and principal component analysis was performed on the standardized expression data of the variable genes using the function `RunPCA`. We used 30 principal components, which explained 90% of the variance, to integrate all samples using the Harmony [33] method with the `Seurat` wrapper function `RunHarmony` with additional parameters (`dim.use = 1:30, group.by.vars = c("Study", "Patient")`). Using the Harmony-corrected cell embeddings, we performed clustering by computing a shared nearest neighbors (SNN) graph, as implemented in `FindNeighbors(reduction = "harmony", dims = 1:30)`. Cluster identification using the SNN graph was computed by the `FindCluster` function (Louvain algorithm). The resolution parameter was set to 1. The Harmony-corrected cell embeddings were projected into a two-dimensional space using the t-distributed stochastic neighbor embedding (tSNE) method.

The number of variable genes and principal components as well as the resolution of the clusters refer to the first iteration of the analysis (see 11.1). Variations of the parameters are mentioned in the section 11.

7.2 Cluster annotation

For the annotation of the cell type populations we followed a three-step approach.

Correlation-based approach: Clusters were annotated with the R package `clustifyr` v1.7.1 [34]. Query clusters were assigned cell types to the highest correlated reference cell type, with a cut-off threshold of 0.5. Query clusters dissimilar to all available reference cell types are labeled as "unassigned." Normalized query and reference expression data, the corresponding metadata tables (assignment of cells to clusters/cell types), and a vector of variable genes were passed to the `clustifyr` function. Here, we used the variable genes estimated with Seurat using `SelectIntegrationFeatures` (see section 7.1). For the annotation of epithelial, stromal, and leukocyte cells, we used the annotated scRNA-Seq data of healthy human prostate samples from Henry et al. [35] as a reference. More precisely, cells in this dataset are annotated as basal epithelial, luminal epithelial, club epithelial, hillock epithelial, neuroendocrine epithelial, fibroblast, smooth muscle, endothelial, or leukocyte. We used then the `seurat_ref` function from `clustifyr` to create a reference object. To subdivide the leukocyte clusters annotated with the reference of Henry et al. into cell types such as B-cells, CD4/CD8 T-cells, NK-cells, monocytes, and neutrophils, we used the TPM (transcript per million)-normalized RNA-Seq data of sorted immune cells (blood) from Racle et al. [36] as a reference. To distinguish between monocytes and dendritic cells, we used the single-cell data from Villani et al. [37] as a reference. The gene sets provided by Jiang et al. [38] were used to annotate Mast cells. This gene set includes the following genes: TPSB2, TPSAB1, TPSD1, TESPA1, RGS13, SLC18A2, CPA3, MS4A2, HPGDS, ADCYAP1, and HDC.

Cell type markers: Cells were verified based on gene expression of the following cell type markers: B-cells, CD19, MS4A1; T-cells, CD2, CD3D, CD3E CD3G, CD4, CD8A, CD8B; NK-cells, KLRF1, FCGR3A; Myeloid lineage, CD68, CD163, CD14, ITAX; Dendritic cells, CD1D, CD1E, CD1C, FCGR2B, FCER1A, HLA-DRA; Macrophage, C1QC, C1QA, CD68, APOE, CD163, FCGR1A; Monocyte/Macrophage, CD14, FCGR3A, CD36; Monocyte, RETN; Endothelium, PECAM1, CD200, CDH5, VWF; Lymphatic endothelium, PROX1; Glia, SOX10, L1CAM; Fibroblasts, PDPN, DCN; Smooth muscle, MYH11, ACTA2, COL5A2; Mesenchymal, COL5A2, FGF10, RSPO3, CXCL5; Basal cell, NGFR, KRT5, KRT14, TP63; Hillock cell, KRT13, KRT4; Club cells, PIGR, MMP7, CP, LTF ; Luminal cells, DPP4, KLK2, KLK3, KLK4, ACPP, PLA2G2A

Annotated cells from the analyzed studies: Clusters' cells annotations for 3 out of 5 datasets [27, 29, 31] were available. We used these cell annotations to validate our results. If the annotated cells from the datasets did not match our annotations, these ambiguous cells were removed. Although this is a conservative filtering step, false positive annotations can be avoided in this way.

8 Differential expression analysis (DGEA)

8.1 Cell types/lineages

For each gene, we calculated the average normalized expression per patient and cell lineage/type. We performed a Wilcoxon rank-sum test between the average gene expression per patient specimen for each cell lineage and the average patient specimens expression of the other lineages. The same

method was applied for cell types in their respective lineage group. We performed this type of DGEA because we wanted to test differences in gene expression at the patient level rather than treating each individual cell as an independent sample. In addition, this approach corrects for the imbalanced representation of different cell lineages/types in patients and reduces zero inflation in scRNA-Seq data with the objective of obtaining more reliable p-values from the DGEA. Testing for differential gene expression was performed using the function `FindMarkers(logfc.threshold = 0.25, only.pos = T)` implemented in `Seurat`. DGEA was performed for a cell type/lineage only if there were at least 3 patient samples with at least 10 cells. This was always the case with the exception of cyclic T-cells. Adjusted p-values for multiple testing were calculated using the Bonferroni method. A gene was considered as significantly differentially expressed (DE) if the FDR-adjusted p-value was <0.05.

8.2 Tumor-specific luminal (T-luminal) vs. luminal cells

Analysis was limited to samples from patients in whom tumor tissue and adjacent normal tissue were available. We performed here a Wilcoxon rank-sum test between T-luminal and luminal cells without average gene expression values from patient as we found no DE genes otherwise. A gene was considered as significantly differentially expressed (DE) if the FDR-adjusted p-value was <0.05.

9 Evaluation of cell type/lineage specific markers

The following metric was applied for cell types and cell lineages. The metric is described below for cell types. As suggested by Adams et al. [39], the cell type specificity of DE genes from the DGEA was assessed using a binary classification system. The aim was to evaluate genes independently of their gene expression for the classification of a cell type cluster. For all DE genes from the DGEA, we calculated the diagnostic odds ratio (DOR), binarizing the expression values. Any detection of a gene (normalized expression value >0) is considered positive, otherwise negative. We sampled 3000 cells from each cell type (10,000 cells for the cell lineages) The DOR is defined as follows:

$$DOR = log(\frac{(TruePositives + 0.5)/(FalsePositives + 0.5)}{(FalseNegatives + 0.5)/(TrueNegatives + 0.5)})$$

where TruePositives represents the number of cells within a cell type detected expressing the gene, FalsePositives represents the number of cells outside of the cell type detected expressing the gene, FalseNegatives represents the number of cells within the cell type with no detected expression, and TrueNegatives represents the number of cells outside of the cell type with no detected expression of the gene. To avoid undefined values, pseudocounts of 0.5 was used. To define gene sets of cell type specific markers, we filtered for uniqe cell type DE genes with a DOR >2 and a log2 fold change >1. For tumor-specific luminal (T-luminal) cell markers, we used a DOR >1. These gene sets were used in spatial transcriptome analysis for spot enrichment.

10 Tumor cell identification using inferCNV

We applied `inferCNV` (R package `infercnv v1.11.1`; https://github.com/broadinstitute/inferCNV) to the epitelial cells of the single cell RNA-Seq data of the PCa atlas to distinguish tumor cells from normal epitelial cells. Epitelial cells of the PCa atlas were splitted in sub-lineages (see Table S8) and analyzed seperately. Normal reference cells were used from the publication by Henry et al. [35] and matched as stated in Table S8. `InferCNV` was run with parameters: `cutoff = 0.1`, `cluster_by_groups = TRUE`, `denoise = TRUE`, `HMM = TRUE`. Homo sapiens (human) genome assembly GRCh38 (hg38) was applied for gene annotation. To reduce the run time of the analysis, the number of reference cells was subsampled to 2500 cells if the total number of associated reference cells exceeded 2500. For the same reason, Luminal cells of the PCa altas were analyzed for each cohort seperately and for Chen et al. [29] the cells were additionally split into three subsets.

Table S8: Test and reference cell lineages for inferCNV

**PCa atlas lineages**	**Reference lineages (Henry et al.)**
Basal Epithelia	Basal Epithelia
Basal/Luminal Epithelia	Basal Epithelia
Club Epithelia	Club Epithelia
Club/Luminal Epithelia	Club Epithelia
Hillock Epithelia	Hillock Epithelia
Luminal Epithelia	Luminal Epithelia
Epithelial (cycling)	Luminal Epithelia

After applying `inferCNV`, a `CNV_score` was determined for each cell i of lineage j considering all genes k as follows:

$$\texttt{CNV\_score}_{i,j} = mean\left((exprs_k - median(exprs))^2\right)$$

Here $exprs$ are the expression values processed by `infercnv` for each gene of the cell. These are close to one in the case of normal copy number and increased or decreased in case of copy-number gains and losses respectively. The `CNV_score` thus combines frequency and strength of potential copy-number aberrations per cell. Cells were classified as tumor cells if the `CNV_score` of the cell was above the 99% percentile of the `CNV_scores` of the normal reference cells from the dataset of Henry et al. [35].

11 Analysis workflow

11.1 First iteration: Post-clustering filtering

In the first iteration, we performed integration, unsupervised clustering, and cell cluster annotation as described in Section 7.1. For the following filtering step, we divided the annotated clusters into

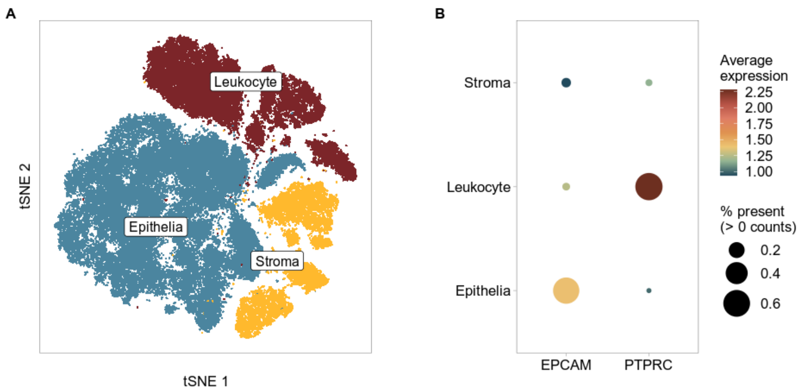

Figure S17: Post-cluster cell filtering. (A) tSNE projection of all cells analyzed. Cells from 79 prostate biopsy tissue samples were partitioned into epithelial, stromal, and leukocytic compartments. **(B)** Expression of EPCAM and PTPRC in the 3 cell compartments. The color intensity indicates the average expression level in a compartment and the circle size reflects the percentage of expressing cells within each compartment of the corresponding genes.

epithelial, stromal, and leukocyte lineages (Figure S17A). The objective of the following cell filtering step was to remove potential contaminating immune and epithelial cells from the single-cell datasets. Droplets encapsulate single cells with small portions of the extracellular environment, resulting in contamination by ambient RNA [40] released from damaged, stressed, and dying cells, which is often exacerbated during the dissociation of solid tissue. Thus, based on two canonical markers, we removed stromal or leukocytic annotated cells in which the epithelial marker EPCAM was present ($\geq$ 1 count). In addition, we removed epithelial cells in which the leukocyte marker PTPRC is present (Figure S17B). We reasoned that each compartment should uniquely express genes not found in other cells. PTPRC present in epithelial cells is most likely contaminated RNA from lysed immune cells. We did not use stromal markers for filtering because, to our knowledge, no markers published in the literature are expressed in all cell types of the stromal cell lineage. After this filtering step, 3717 cells were removed.

11.2 Second iteration: QC and cell cluster annotation

All 93,150 remaining cells were re-integrated, clustered and annotated. We performed a tSNE projection and colored all cells based on several characteristics (Figure S18). Figure S18H shows that some cell communities have lower heterogeneity and are more associated with the datasets of the respective studies. Therefore, we evaluated the effect of integration (batch correction) by the harmony method (see section 7.1) using an entropy-based metric. Following the methodology described previously [41], we constructed a k-nearest neighbor graph (k=200) with 30 principal components before integration and with harmony-corrected components. The k-nearest neighbor graph was generated using the `FindNeighbors` function implemented in `Seurat` for tumor and normal specimens, respectively. Then, we computed the fraction of cells q_P derived from each patient P in the neighborhood of each cell j. We then calculated the Shannon entropy $H_j = \sum_P q_P \log q_P$ of sample frequencies within each cell's neighborhood. High entropy indicates that the most similar

cells come from a well-mixed set of patients, whereas low entropy indicates that most similar cells derive from the same patient. We compared the distribution of entropies across all cells from tumors and normal tissues before and after integration (Figure S19A and B). The entropy shifts significantly toward higher mixing of patients after processing with `harmony` (Mann-Whitney U-test: p-value $< 2.2e{-}16$ for tumor and normal tissues). We thus conclude that the `harmony` method substantially corrected batch effects in this data. The entropy values were embedded into a two-dimensional space by the tSNE method (Figure S19C and D). However, we found that some cell communities have lower entropy, indicating sample-specific cell communities. It should be noted that even after batch correction, data integration remains imperfect, particularly due to tissue complexity, diversity in cell types and high technical variability (Figure S18G-I). As a result, batch effects could impact biological data interpretation and downstream applications, requiring validation of observations through further experimentation.

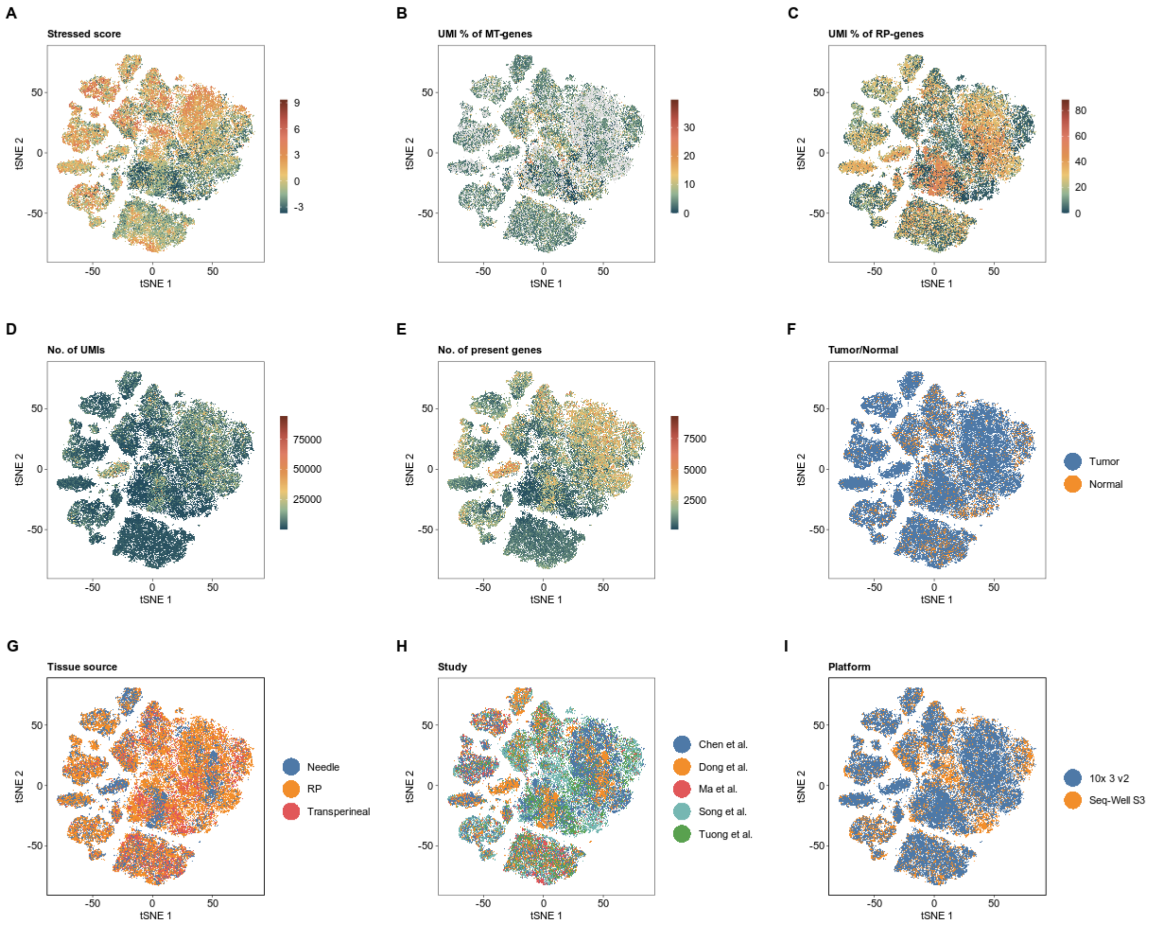

Figure S18: Dimensionality reduction after integration. tSNE embedding of single cells from 79 prostate biopsy tissue samples colored by different characteristics. **(A)** Stressed Score: Possible dissociation-related artefacts caused by cellular stress due to solid tissue dissociation into single-cell suspensions [42, 43]. For this purpose, the methodology and stress signature proposed by Henry et al. [35] was applied. **(B-C)** The fraction of UMIs from mitochondrial (MT) and Ribosomal (RPx) genes per cell. No MT genes are available for the dataset from the study by Chen et al. (gray colored dots).

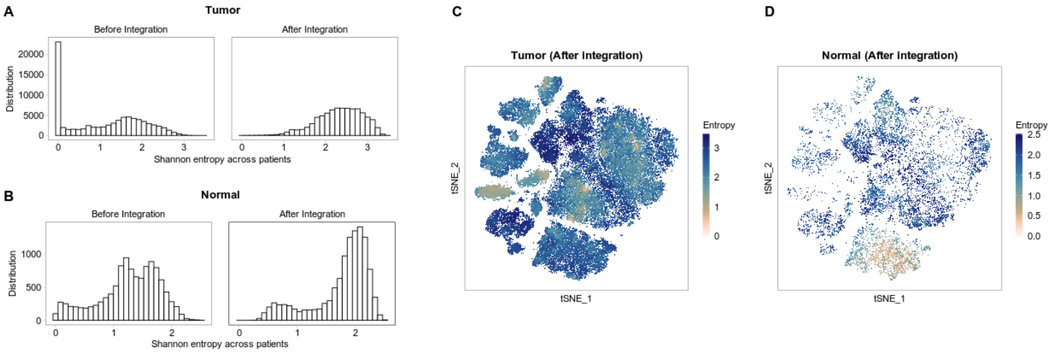

Figure S19: Entropy Metric to evaluate integration. Histogram of Shannon entropy of the distribution of **(A)** tumor tissue (patient) and **(B)** normal tissue (patient) as a measure of sample mixing. Entropy distribution is shown before and after integration. tSNE projection of the **(C)** tumor tissue and **(D)** normal tissue colored by Shannon entropy of patients in a k-nearest neighborhood of each cell.

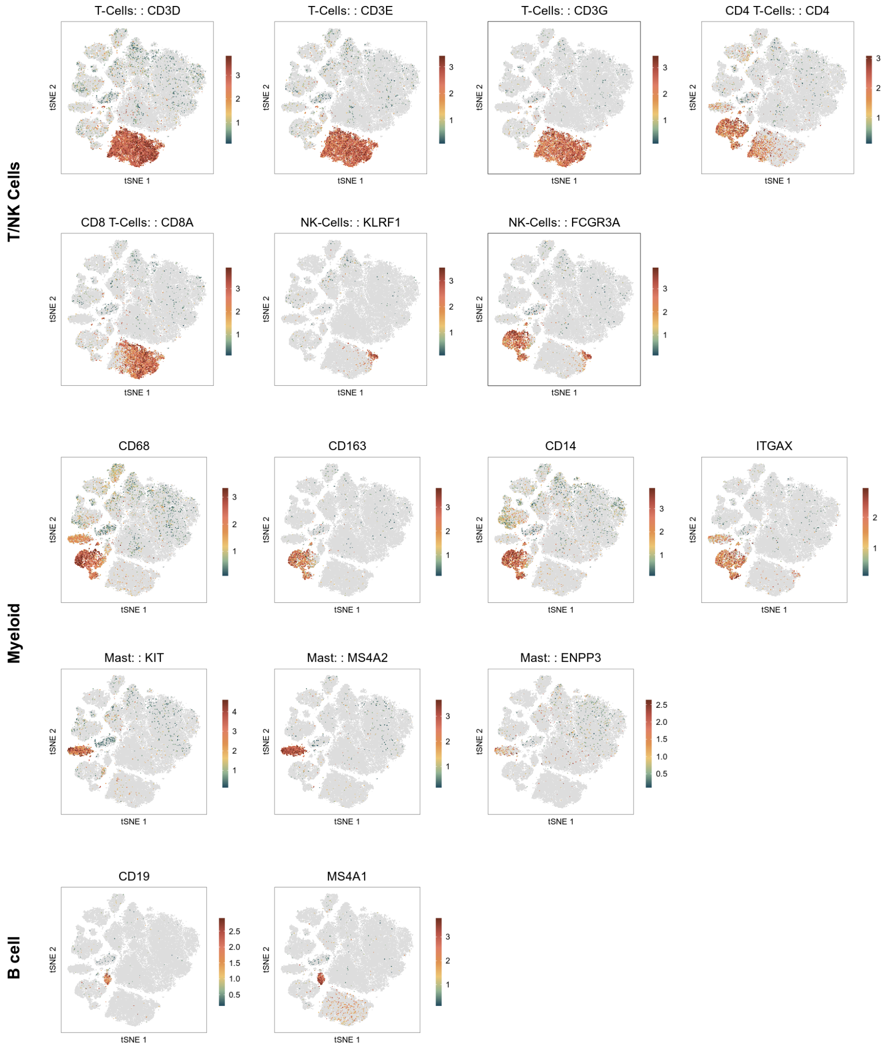

Figure S20: Expression of leukocytic markers. tSNE expression plot of marker genes for leukocytic cells. An increasing color gradient from blue to red corresponds to an increasing normalized expression level. Gray colored cells indicate no expression.

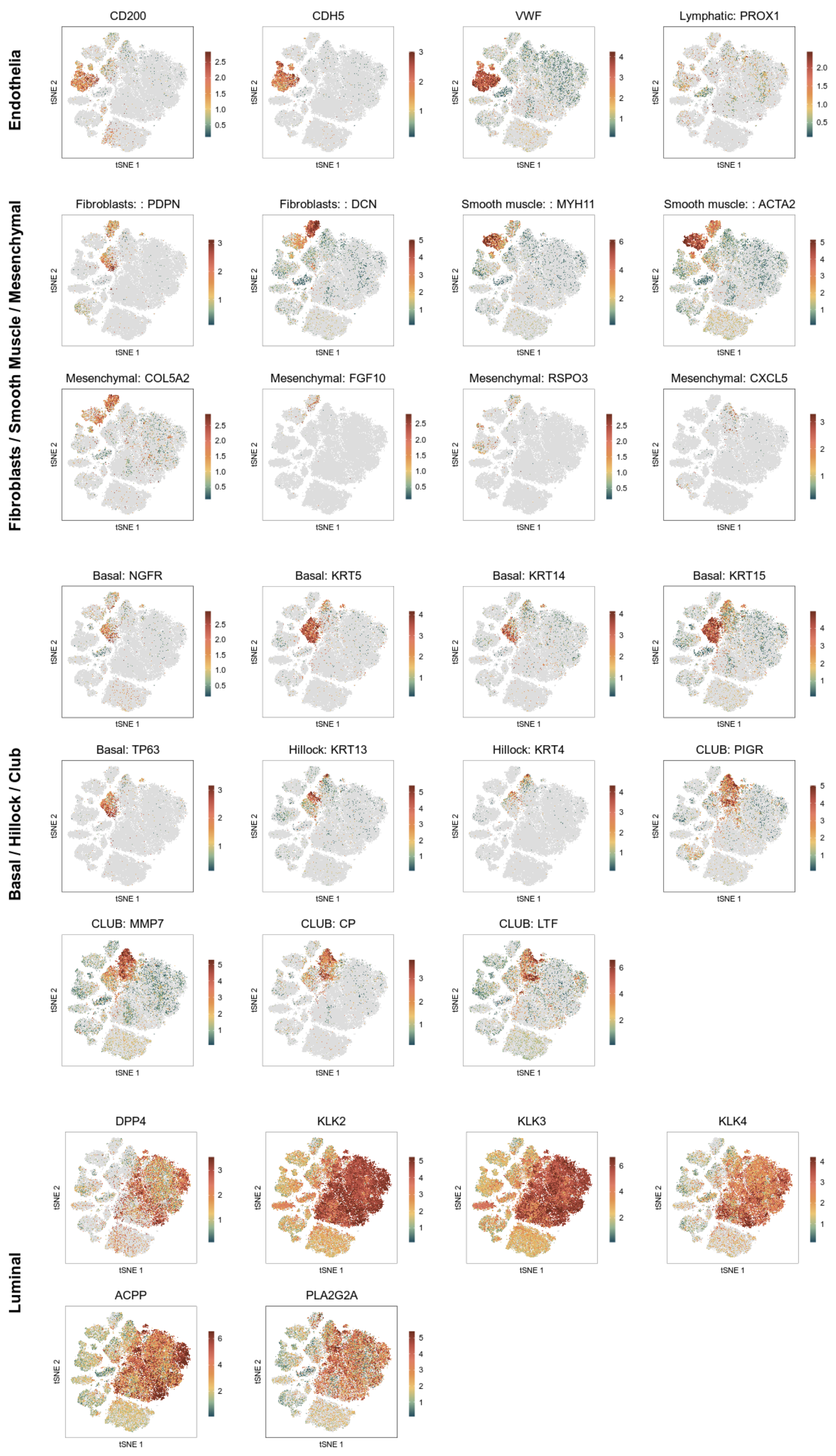

Figure S21: Expression of stromal and epithelial markers. tSNE expression plot of marker genes for stromal and epithelial cells. An increasing color gradient from blue to red corresponds to an increasing normalized expression level. Gray colored cells indicate no expression.

11.2.1 Coarse grain cell cluster annotation

The cluster resolution value was empirically determined for the `FindClusters` function implemented in `Seurat`. Cluster patterns were analyzed by iteratively evaluating the resolution value between 0.1 and 1. The resolution was set at 0.5, representing a balance between the over-segmentation of cell types and the effective separation of clusters embedded in tSNE. In other words, we tried to select a cluster resolution that results in the highest possible granularity of clusters while still being biologically distinct, as determined by inspection of cell type marker genes (see Figure S20 and S21). Results of the cluster analysis are shown in Figure S22A. Cluster cell annotations of epithelial, stromal, and leukocyte cell types estimated by `clustifyr` using the reference from Henry et al. [35] (see 7.2) are shown in Figure S22C.

Cell cycle scores for each cell were calculated based on the expression of previously published G2/M and S-phase gene sets [44] using the CellCycleScoring function implemented in `Seurat` (Figure S22E and F). This score represents an average relative expression level of each gene set on single-cell level, subtracted by the aggregated expression of background gene sets. All analyzed genes were divided in 24 equal-frequency expression bins based on averaged expression, and the background genes were randomly selected from each bin. To identify significant cluster-level changes, we applied the `run_gsea` function implemented in the `clustifyr` package using the G2/M and S-phase gene sets as queries. The number of permutations was set to 1000 (Figure S22G). Cluster 12 was significantly (p-value <0.05) enriched with cell cycle genes from the G2/M phase. However, within the cell cycle cluster, cells of distinct lineages clustered together. These cells were then separated back into their source lineages.

11.2.2 Fine grain cell cluster annotation

Comparing the annotated cell clusters 3 and 6 (Figure S22A and C) with the cell annotations of the scRNA-Seq data from the study by Tuong et al, differences in annotation can be observed (Figure S22B). For example, according to Tuong et al, the cells in cluster 6 are divided into basal and club epithelial cells. The expression of marker genes for these two cell types can also be observed in Figure S21. In addition, the correlation coefficients for each cluster and each cell type estimated by `clustifyr` are depicted in Figure S22D. The 3 highest correlation coefficients for cluster 6 also indicate that this cluster consists of multiple cell types.

Since higher cluster resolution did not separate these cell types, we decided to use a sub-clustering approach outlined in Haber et al. [45]. To cluster single cells into distinct cell subsets, we sub-clustered the cells within cluster 6, which entails the selection of variable genes, integration, dimensionality reduction, graph clustering (see 7.1) and cluster annotation (see 7.2). Since this analysis is limited to a few cell types, we used 1000 variable genes, 10 principal components and a cluster resolution of 1. The same approach was performed for cluster 3. Using this strategy, we were able to re-annotate cluster 3 as club cells and cluster 6 as basal and hillock cells. In addition, basal and club

cell populations were distinguished by sub-clusters that had high KLK3 expression (data not shown). Differential gene expression analysis (see 8) showed that KLK3 was significantly regulated in these sub-clusters compared to the others (see Figure S23). We did not assume that the subclusters were exclusively luminal cells because they expressed both basal/club markers and luminal markers. For this, compare Figure 3A (cell cluster annotation) in the main part with Figure S21 (expression of cell type markers). These sub-clusters were annotated with the suffix "KLK3".

Cell clusters assigned to the leukocyte cell lineage were subdivided into myeloid and lymphoid cell types using RNA-Seq data of sorted immune cells (blood) from Racle et al. [36] as reference (Figure S24A). Cluster 10 was annotated by `clustifyr` as an NK cell cluster. However, Figure S20 shows that gene markers for mast cells and not NK cells are expressed in this cluster. In addition, a low correlation coefficient was observed between cluster 10 and NK cell type (Figure S24A). Because the reference used for annotation does not contain expression data for mast cells, we performed GSEA using a gene set with markers for mast cells [38] as a query. As shown in Figure S24C, cluster 10 was significantly enriched in the gene set for mast cells.

The sub-cluster approach also allowed us to further refine the annotations of lymphoid and myeloid cell types. For myeloid cell types, we additionally used scRNA-Seq data of monocytes and dendritic cells from the study Villani et al.[37] as a reference (see Figure S25 and S26).

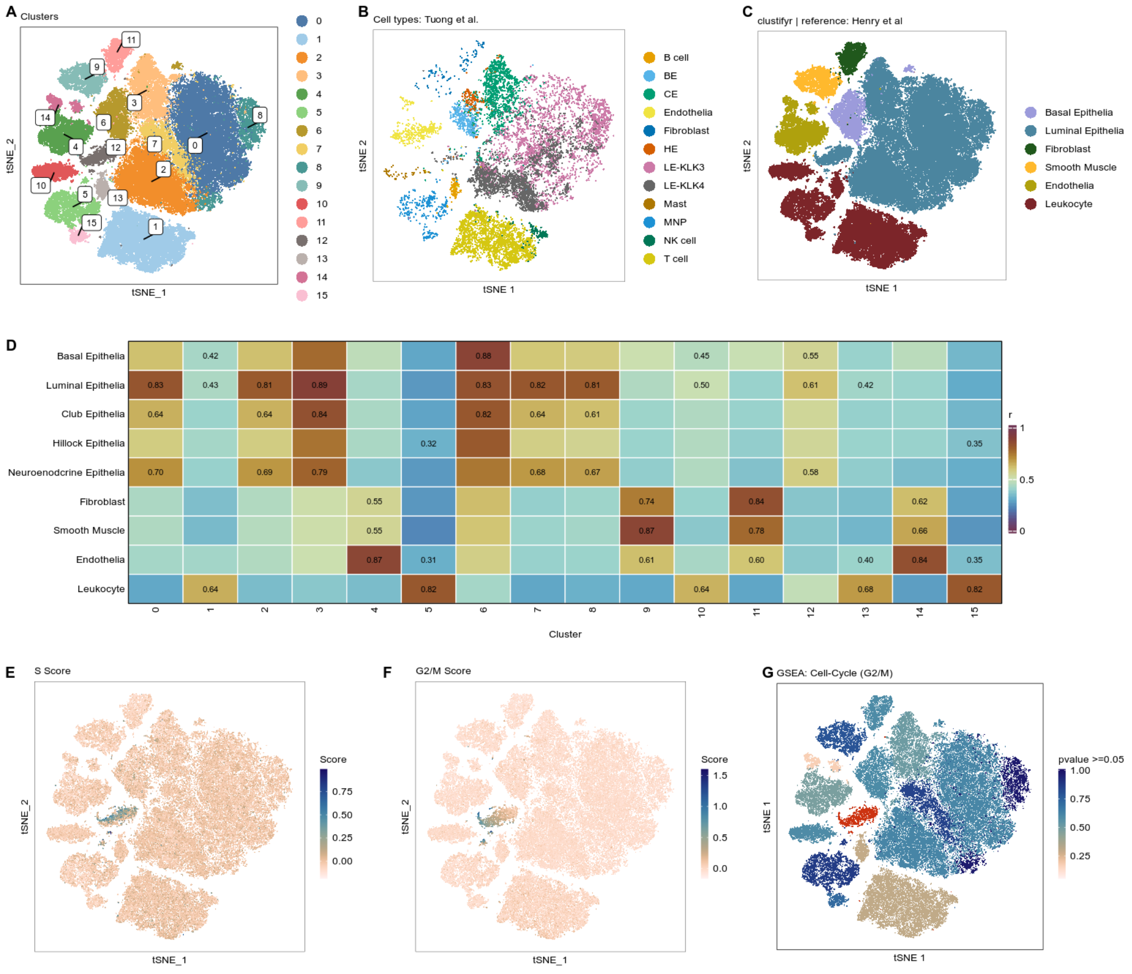

Figure S22: Cluster cell annotations of epithelial and stromal cell types and leukocyte lineage (A) Cell are colored by clusters. **(B)** Cell annotations of the scRNA-Seq data from the Tuong et al. study [31]. **(C)** Cell clusters color-coded by cell types using annotated scRNA-Seq data of healthy human prostate samples from Henry et al. [35] as a reference. Clusters were annotated with the `R` package `clustifyr`. **(D)** Correlation coefficients for each cluster and each cell type estimated by `clustifyr`. For each cluster, the 3 highest correlation coefficients are shown. **(E-F)** Cells are colored according to the cell cycle score for S and G2/M phase estimated using the `CellCycleScoring` function in `Seurat`. **(G)** Clusters are colored by p-values estimated from GSEA using the cell-cycle gene set for G2/M phase [44] as query. Red colored cluster has a p-value <0.05.

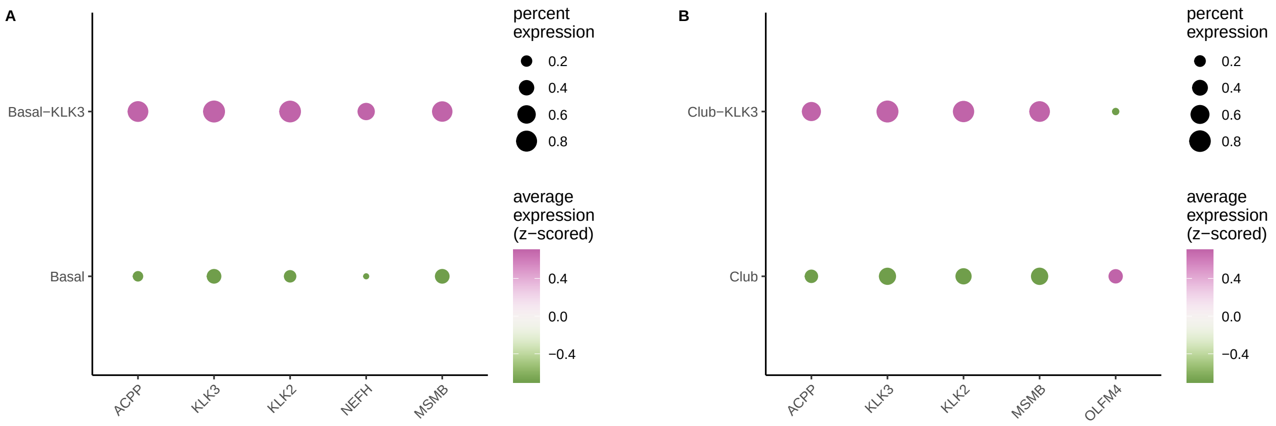

Figure S23: DGEA for basal and club cell types characterized by sub-clusters with high KLK3 expression. 5 highest ranked significantly (FDR <0.05) differentially expressed genes by log fold change for **(A)** basal cell and **(B)** club cells. The color intensity indicates the average standardized expression level and the circle size reflects the percentage of expressing cells within each sub-cluster.

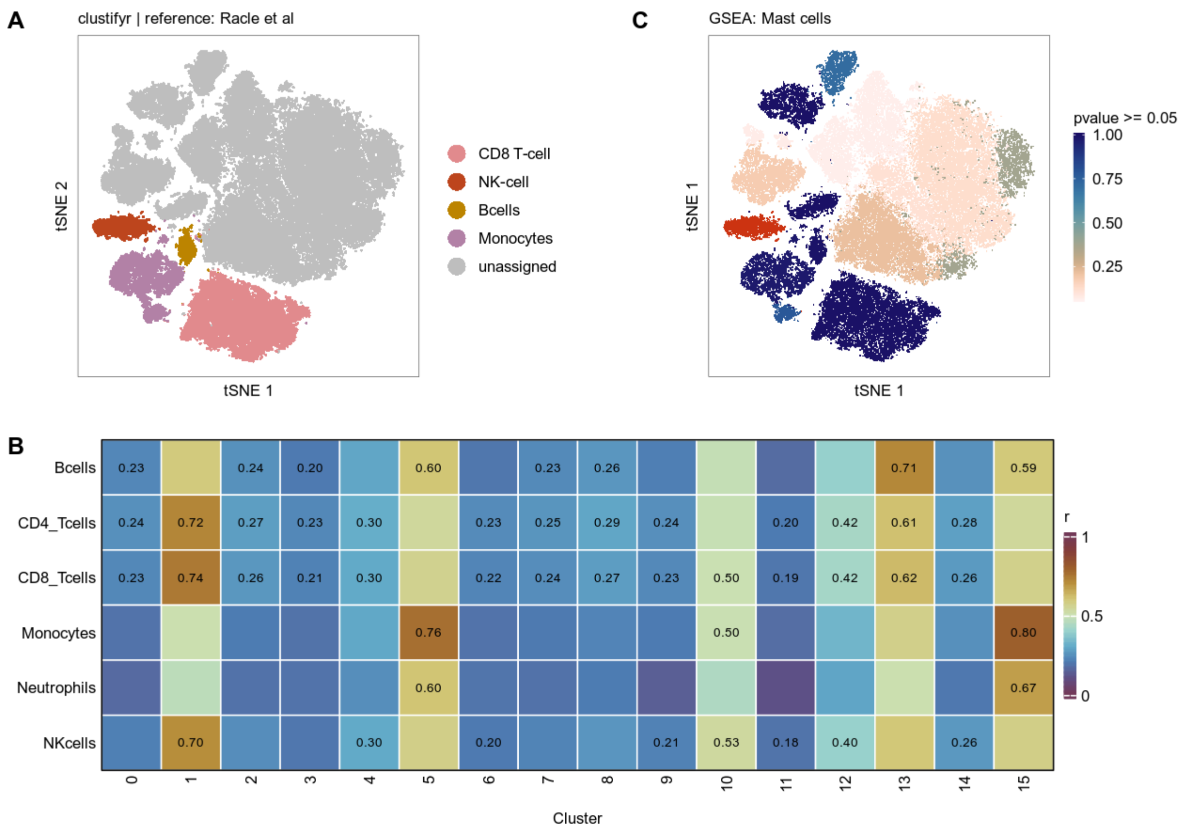

Figure S24: Cluster cell annotations of leukocyte cell types. **(A)** Cell clusters are color coded according to the highest correlated reference cell type using the R package `clustifyr`. **(B)** Correlation coefficients for each cluster and each cell type estimated by `clustifyr`. For each cluster, the 3 highest correlation coefficients are shown. **(C)** Clusters are colored according to p-values estimated from the GSEA with 1000 permutations using a gene set for mast cell [36] as query. Red colored cluster indicate a p-value <0.05.

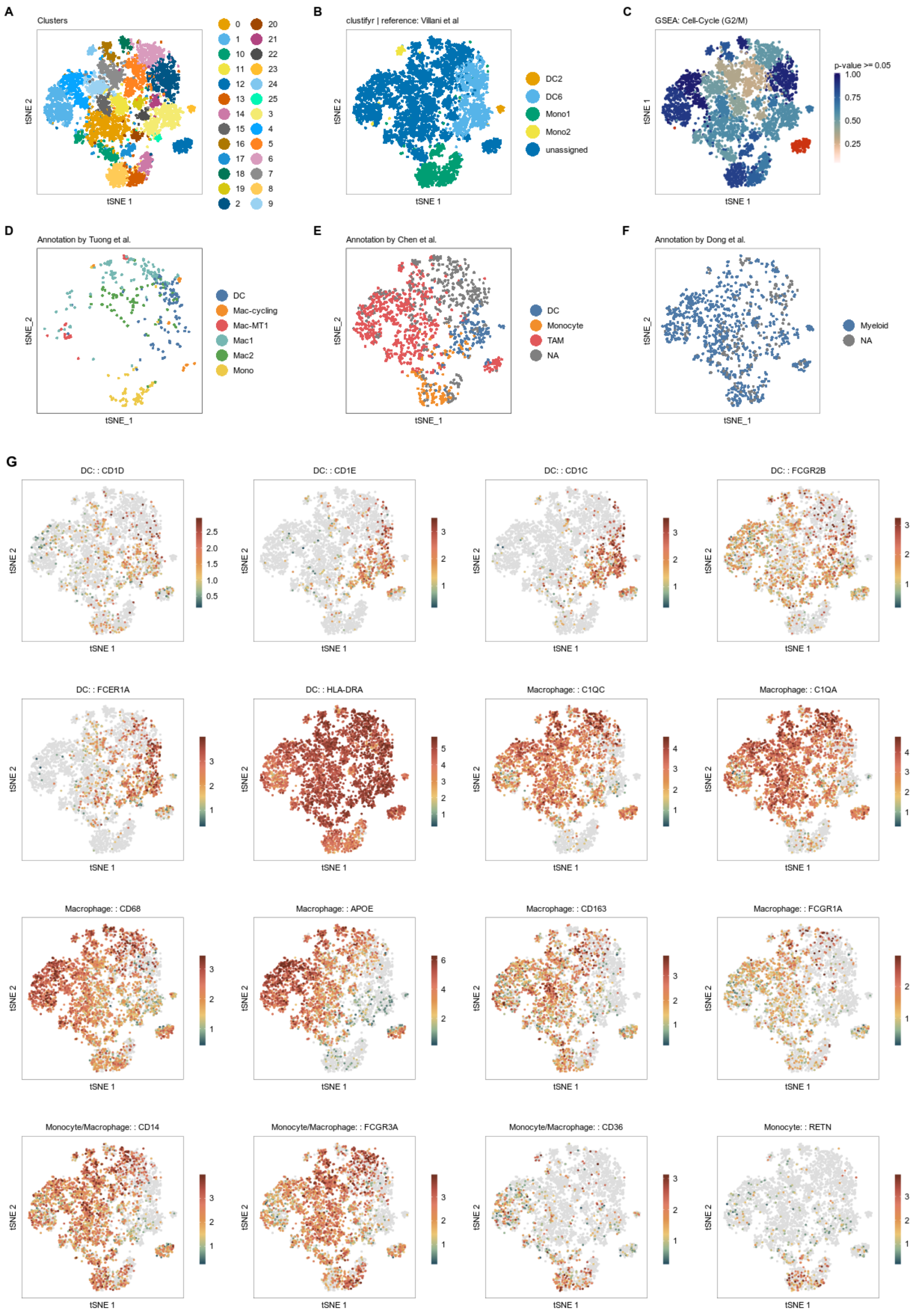

Figure S25: Identification of sub-clusters and cell type annotation for myeloid cells. **(A)** Cells are colored by clusters. **(B)** Cell clusters color-coded by cell types using scRNA-Seq data of monocytes and dendritic cells from Villani et al. [37] as a reference. Clusters were annotated with the R package `clustifyr`. **(C)** Clusters are colored by p-values estimated from GSEA using the cell-cycle gene set for G2/M phase [44] as query. Red colored cluster has a p-value <0.05. **(D)** Expression plot of marker genes for monocytes, dendritic and macrophage cells. Increasing color gradient from blue to red corresponds to increasing normalized expression value. Grey indicates no expression.

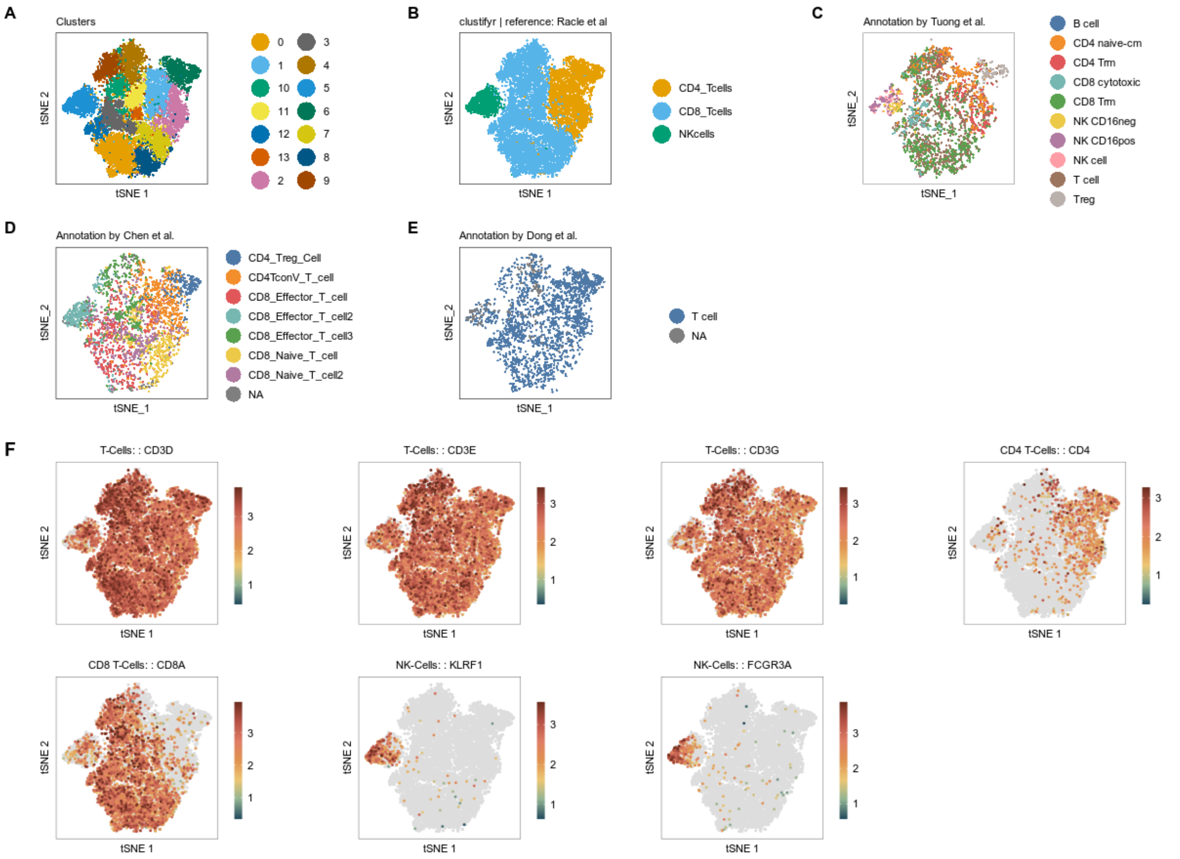

Figure S26: Identification of sub-clusters and cell type annotation for T-cells and NK-cells. (A) Cells are colored by clusters. **(B)** Cell clusters color-coded by cell types using RNA-Seq data of sorted immune cells (blood) from Racle et al. [36] as a reference. Clusters were annotated with the `R` package `clustifyr`. **(C-D)** Cell annotations of scRNA-Seq data from Tuong et al, Chen et al, and Dong et al studies. **(F)** Expression plot of marker genes for T-cells and NK-cells. Increasing color gradient from blue to red corresponds to increasing normalized expression value. Grey indicates no expression.

11.3 Final PCa atlas

Overall, we identified 15 cell types derived from lymphoid, myeloid stromal and epithelial cell lineages (Figure S27A). Regarding the composition of cell types, cells from all 5 single-cell studies were present in all cell types (Figure S27B). However, it should be noted that the cycling cell types of the study by Ma et al. are represented by only few cells (Figure S27B). Overall, the number of cells ranged from 41229 cells for the luminal cell type to 113 cells for cycling T-cells. Since prostate cancer is characterized by a luminal phenotype, we expected a high number of the corresponding cell type.

In addition, we quantified the diversity between patients for each cell type. For this purpose, we used the Shannon entropy-based metric as in Section **??**. In addition, we performed a bootstrap approach to account for the different number of cells per cell type. We sampled 100 cells from each cell type 1000 times with replacement and computed the Shannon entropy across patients m = 1,. . . ,N for each subsample H_s according to $H_s = \sum_{m=0}^{N} p_i^m \log p_i^m$, where p_i represents the fraction of cells of cell type i in patient m. Figure S27C indicates entropy values for each cell types, with the order of cell types based on their median entropy. Cell types with entropy of 0 denote entirely patient-specific cell types. Mast and cycling epithelial cells have the lowest degree of patient mixing, while club-KLK3, dendritic (DC) and luminal cells shows the highest degree. To evaluate whether the entropy values correlate with the number of cells, the cell types in Figure S27D were ordered according to the corresponding number of cells. The correlation (Spearman) between median entropy and the number of cells per cell type was 0.14 indicates that entropy does not correlate with size.

We also performed the entropy and cell type composition analysis separately for tumors and adjacent normal tissue (Figure S28). The correlation between median entropy and the number of cells per cell type was 0.13 for tumor specimens and 0.25 for normal specimens. In tumor (5 studies) and adjacent normal (2 studies) tissue samples, cells from all corresponding studies were present in all cell types (Figure S28E and F). Lastly, we associated genes with cell types by performing DGEA (see Section 8 for details). The 5 highest ranked DE genes for each cell type are shown in Figure S27E.

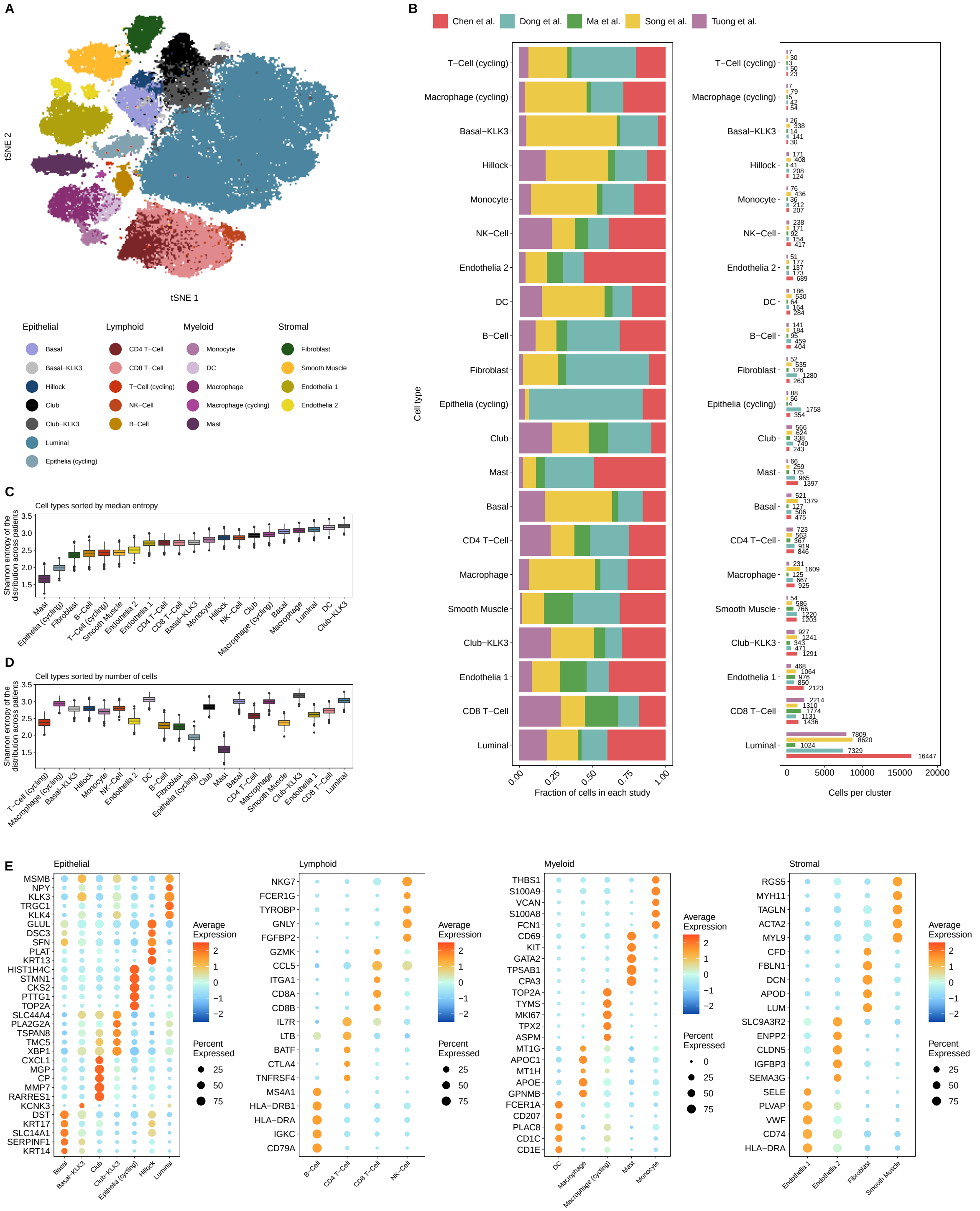

Figure S27: PCa single cell atlas. (A) Cell clusters are color coded by cell type. **(B)** The left stacked bar chart depicts the composition of cell types in each study. The right bar chart depicts the number of cells per cell type. **(C and D)** Boxplots indicate the entropy distribution of patients in each cell type. Entropy values computed with bootstrapping to correct for number of cells in each cell type. Entropy was calculated for each patient specimen type (Separate for tumor and adjacent normal tissue, if present). Cell type ordered by median entropy **(C)** and number of cells **(D)**. **(E)** DE genes for each cell type were determined using the Wilcoxon rank sum test. Genes with an FDR <0.05 and log fold change >0.25 were considered statistically significant. Genes are ranked by log fold changes. For each cell type, 5 significant genes are shown. The color intensity indicates the average expression level in a cell type and the circle size reflects the percentage of expressing cells within each cell type of the corresponding genes.

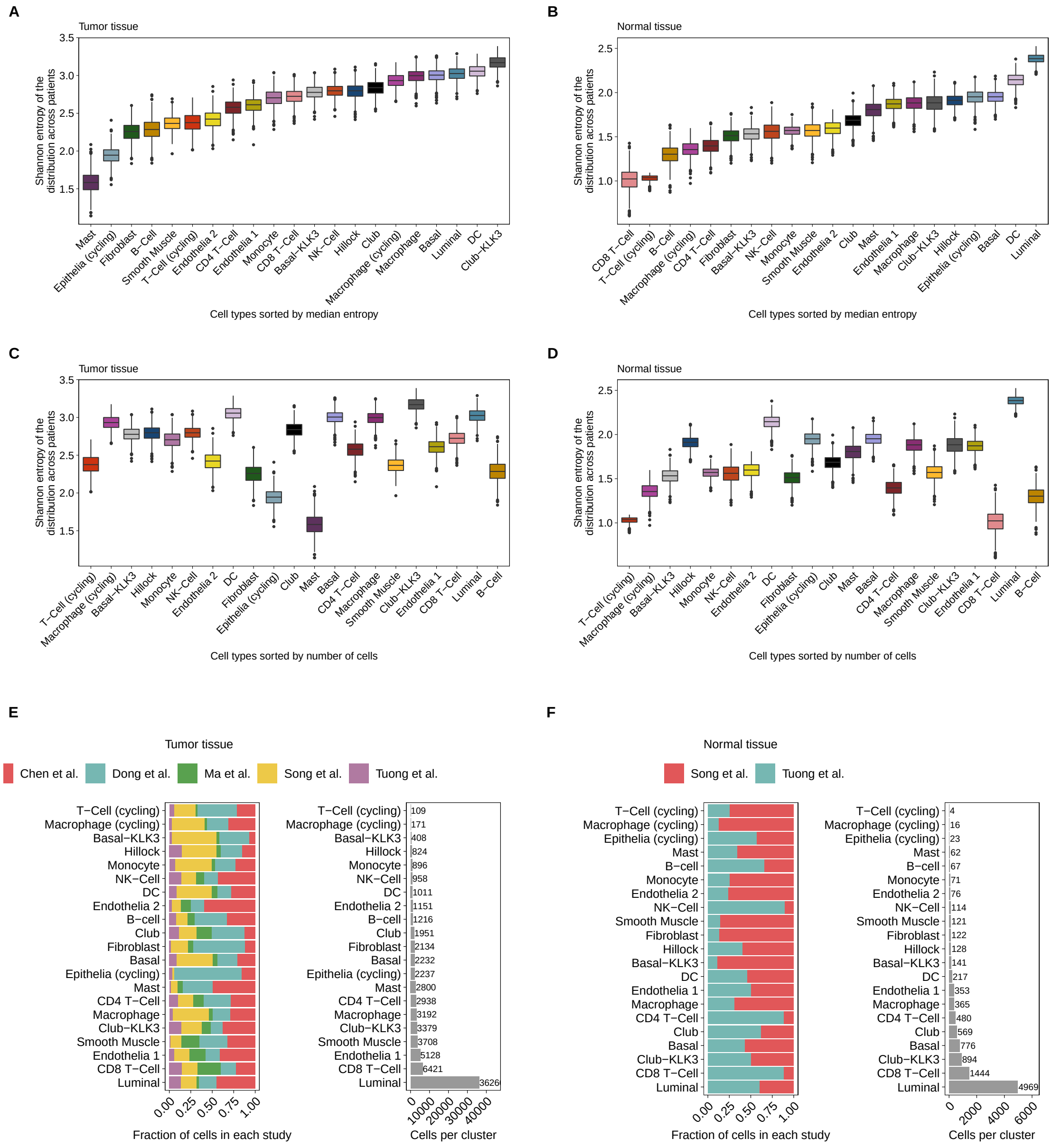

Figure S28: Inter-patient and study heterogeneity per cell type. (A-D) Boxplots indicate the entropy distribution of patients in each cell type. Entropy values computed with bootstrapping to correct for number of cells in each cell type (see 11.3). Entropy was calculated for **(A, C)** tumor and **(B, C)** adjacent normal specimens. Cell types ordered by median entropy **(A, B)** and number of cells **(C, D)**. **(E, F)** Left stacked bar chart depicts the composition of cell types in each study. The right bar chart depicts the number of cells per cell type.

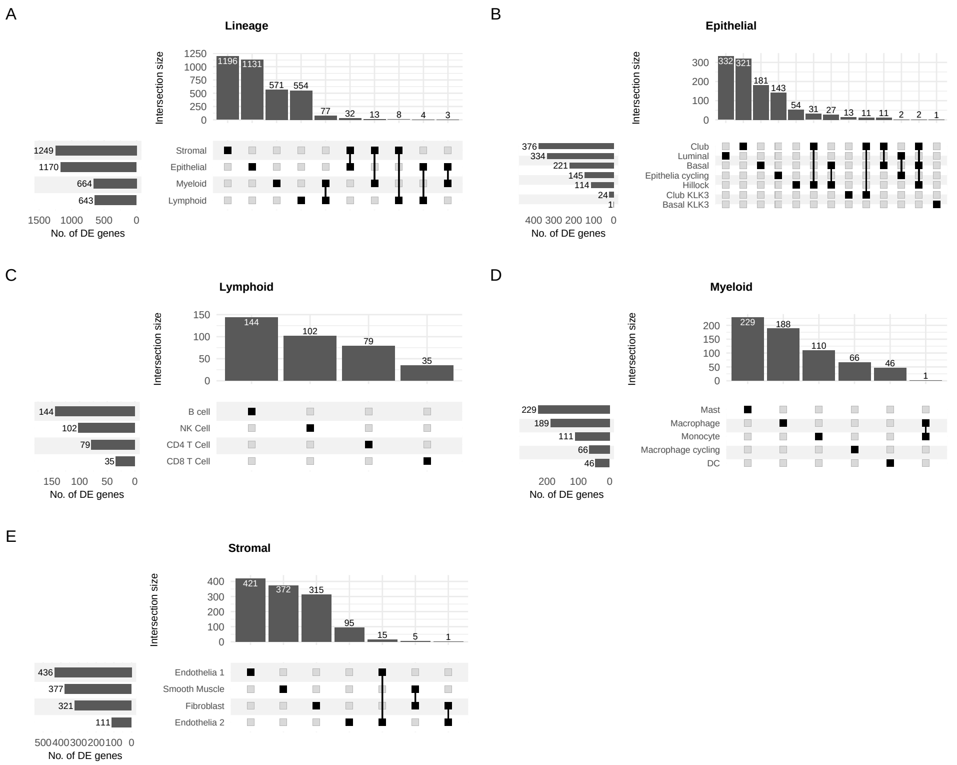

Figure S29: DGEA for cell lineages/types. UpSet plot of DE genes (FDR <0.05) of **(A)** cell lineages and **B-E** cell types. The number of DE genes are indicated as horizontal bar plots. The squares in the matrix represent unique and overlapping DE genes for the cell lineages/types. Connected squares indicate a certain intersection of DE genes between the cell lineages/types. The bar graph above the matrix summarizes the number of DE genes for each unique or overlapping combination.

A

Cell lineage	Gene_Ratio	Bg_Ratio	pvalue	p_value_adjusted
Epithelial	111/1084	1170/16058	0.000267	1.07e−03 *
Lymphoid	43/1152	643/16585	0.561000	1.00e+00
Myeloid	44/1151	664/16564	0.588000	1.00e+00
Stromal	87/1108	1249/15979	0.455000	1.00e+00

B

Cell type	Gene_Ratio	Bg_Ratio	pvalue	p_value_adjusted
Basal	12/1183	221/17007	7.70e−01	1.00e+00
Basal−KLK3	0/1195	1/17227	6.94e−02	4.86e−01
Club	42/1153	376/16852	8.46e−04	5.92e−03 *
Club−KLK3	0/1195	24/17204	8.22e−01	1.00e+00
Epithelia (cycling)	60/1135	145/17083	8.00e−33	5.60e−32 *
Hillock	7/1188	114/17114	5.39e−01	1.00e+00
Luminal	47/1148	334/16894	1.17e−06	8.22e−06 *
B−cell	8/1187	144/17084	6.75e−01	1.00e+00
CD4 T−Cell	4/1191	79/17149	6.48e−01	1.00e+00
CD8 T−Cell	2/1193	35/17193	4.42e−01	1.00e+00
NK−Cell	6/1189	102/17126	5.68e−01	1.00e+00
DC	3/1192	46/17182	3.97e−01	1.00e+00
Macrophage	13/1182	189/17039	4.39e−01	1.00e+00
Macrophage (cycling)	37/1158	66/17162	2.70e−27	1.35e−26 *
Mast	19/1176	229/16999	1.71e−01	8.53e−01
Monocyte	3/1192	111/17117	9.54e−01	1.00e+00
Endothelia 1	25/1170	436/16792	8.16e−01	1.00e+00
Endothelia 2	7/1188	111/17117	5.08e−01	1.00e+00
Fibroblast	31/1164	321/16907	2.46e−02	9.85e−02
Smooth Muscle	23/1172	377/16851	7.00e−01	1.00e+00

Table S9: Enrichment of DE gene with ProstaTrend genes. We performed a hypergeometric test to determine whether the DE from DGEA were enriched with DE genes from the ProstaTrend-ffpe signatures. Adjusted p-values for multiple testing were calculated using the Bonferroni method. Gene sets with an adjusted p-value <0.05 have an asterisk as suffix. Gene Ratio: Number of ProstaTrend genes present in the gene set. Bg Ratio: Number of genes from the gene sets present among all analyzed genes. **A** DE of cell lineages and **B** DE of cell types

12 TRS applied to cells from the PCa atlas

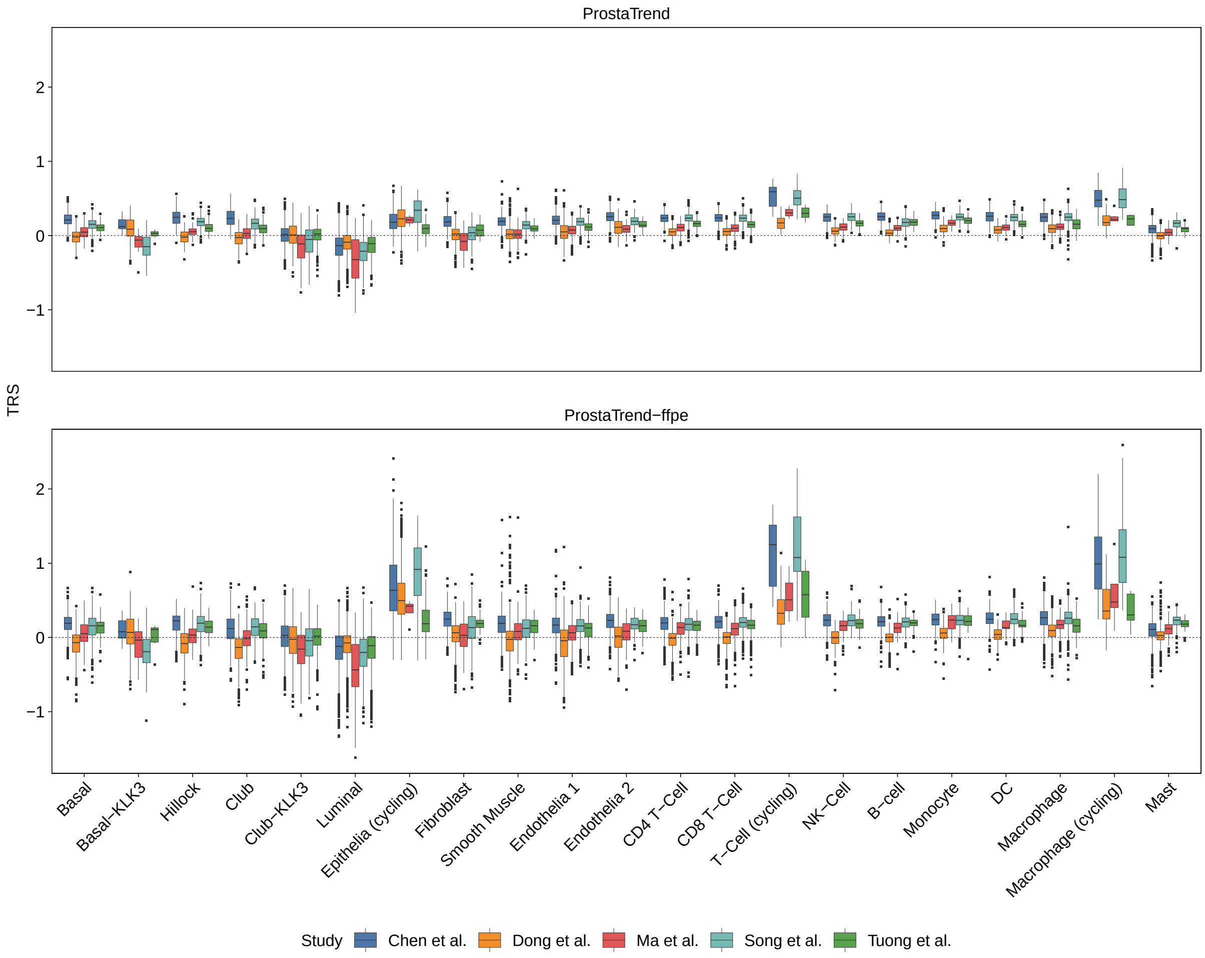

Figure S30: TRS for each cell type. We applied a simplified TRS to each cell from the tumor samples using the ProstaTrend **(A)** and ProstaTrend-ffpe **(B)** signatures. Boxplots are grouped by cell type and colored by study.

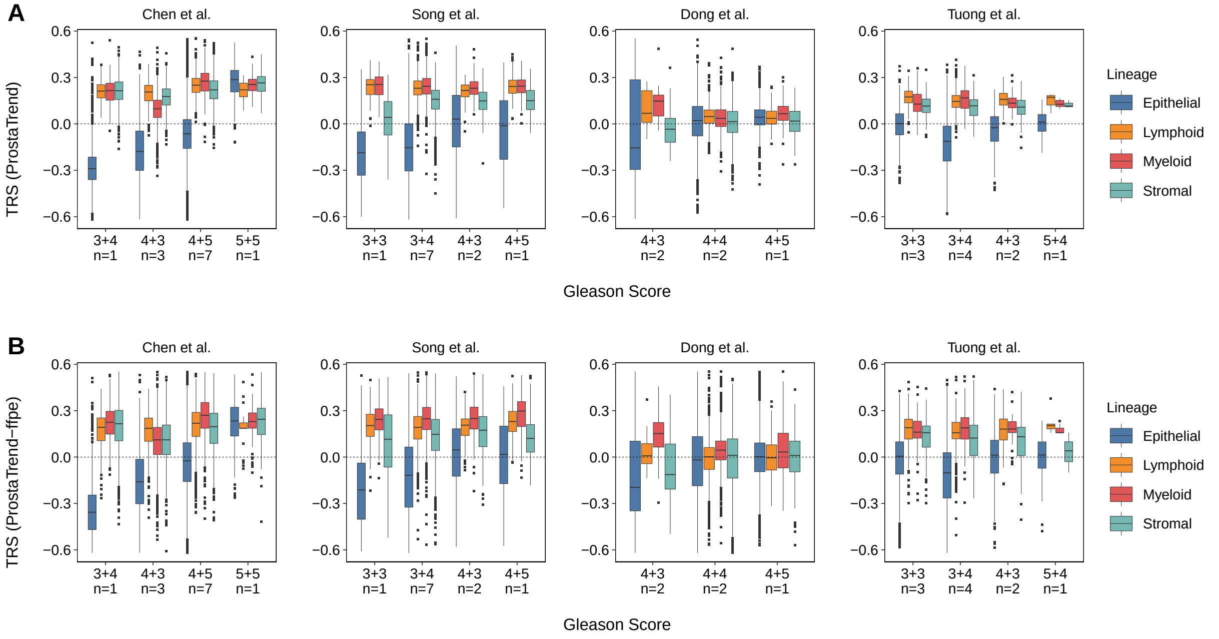

C

ProstaTrend

	Cor	pvalue	pvalue_adjusted
Epithelial; Chen et al.	0.434	<2.20e−16	<2.20e−16
Epithelial; Song et al.	0.199	<2.20e−16	<2.20e−16
Epithelial; Tuong et al.	−0.099	1.92e−14	3.83e−13
Epithelial; Dong et al.	0.167	<2.20e−16	<2.20e−16
Lymphoid; Chen et al.	0.235	<2.20e−16	<2.20e−16
Lymphoid; Song et al.	−0.031	1.73e−01	1.00e+00
Lymphoid; Tuong et al.	−0.174	1.70e−11	3.41e−10
Lymphoid; Dong et al.	−0.022	2.70e−01	1.00e+00
Myeloid; Chen et al.	0.452	<2.20e−16	<2.20e−16
Myeloid; Song et al.	−0.031	1.28e−01	1.00e+00
Myeloid; Tuong et al.	0.079	1.65e−01	1.00e+00
Myeloid; Dong et al.	0.037	1.14e−01	1.00e+00
Stromal; Chen et al.	0.022	1.53e−01	1.00e+00
Stromal; Song et al.	0.103	6.62e−06	1.32e−04
Stromal; Tuong et al.	−0.065	1.98e−01	1.00e+00
Stromal; Dong et al.	0.028	1.90e−01	1.00e+00

D

ProstaTrend−ffpe

	Cor	pvalue	pvalue_adjusted
Epithelial; Chen et al.	0.441	<2.20e−16	<2.20e−16
Epithelial; Song et al.	0.201	<2.20e−16	<2.20e−16
Epithelial; Tuong et al.	−0.029	2.34e−02	4.67e−01
Epithelial; Dong et al.	0.066	5.48e−08	1.10e−06
Lymphoid; Chen et al.	0.114	1.96e−10	3.91e−09
Lymphoid; Song et al.	0.054	1.64e−02	3.28e−01
Lymphoid; Tuong et al.	−0.068	8.48e−03	1.70e−01
Lymphoid; Dong et al.	−0.015	4.54e−01	1.00e+00
Myeloid; Chen et al.	0.311	<2.20e−16	<2.20e−16
Myeloid; Song et al.	0.050	1.28e−02	2.56e−01
Myeloid; Tuong et al.	0.047	4.09e−01	1.00e+00
Myeloid; Dong et al.	−0.047	4.26e−02	8.52e−01
Stromal; Chen et al.	−0.065	2.01e−05	4.03e−04
Stromal; Song et al.	0.051	2.45e−02	4.91e−01
Stromal; Tuong et al.	−0.088	8.15e−02	1.00e+00
Stromal; Dong et al.	0.026	2.28e−01	1.00e+00

E

ProstaTrend

	Cor	pvalue	pvalue_adjusted
Luminal; Chen et al.	0.413	<2.20e−16	<2.20e−16
Luminal; Song et al.	0.072	5.33e−09	1.07e−07
Luminal; Tuong et al.	−0.091	1.95e−10	3.90e−09
Luminal; Dong et al.	0.697	<2.20e−16	<2.20e−16

F

ProstaTrend−ffpe

	Cor	pvalue	pvalue_adjusted
Luminal; Chen et al.	0.424	<2.20e−16	<2.20e−16
Luminal; Song et al.	0.119	<2.20e−16	<2.20e−16
Luminal; Tuong et al.	−0.023	1.16e−01	1.00e+00
Luminal; Dong et al.	0.561	<2.20e−16	<2.20e−16

Figure S31: TRS grouped by Gleason scores and colored by cell lineages. We applied a simplified TRS to each cell from the tumor samples using the ProstaTrend **(A)** and ProstaTrend-ffpe **(B)** signatures. Boxplots are grouped by Gleason scores, divided by single-cell studies, and colored by cell lineages. n = number of patients. We did not include the data from Ma et al. because only two patients with low Gleason scores were available (Gleason scores of 6 (3 + 3) and 7 (3 + 4)). For one patient (Dong et al.), the Gleason score was reported as "small cell NEPC." The single-cell data for this patient was excluded for this analysis. For **(A)** and **(B)**, only data between the 0.01 and 0.99 percentile are shown. The Spearman correlation between the cell lineages for each study and the risk scores is shown in **(C)** and **(D)**. Significance was estimated using the `cor.test` function. Adjusted p-values for multiple testing were calculated using the Bonferroni method. In **(E)** and **(F)**, the correlation between luminal cell type for each study and risk scores is shown.

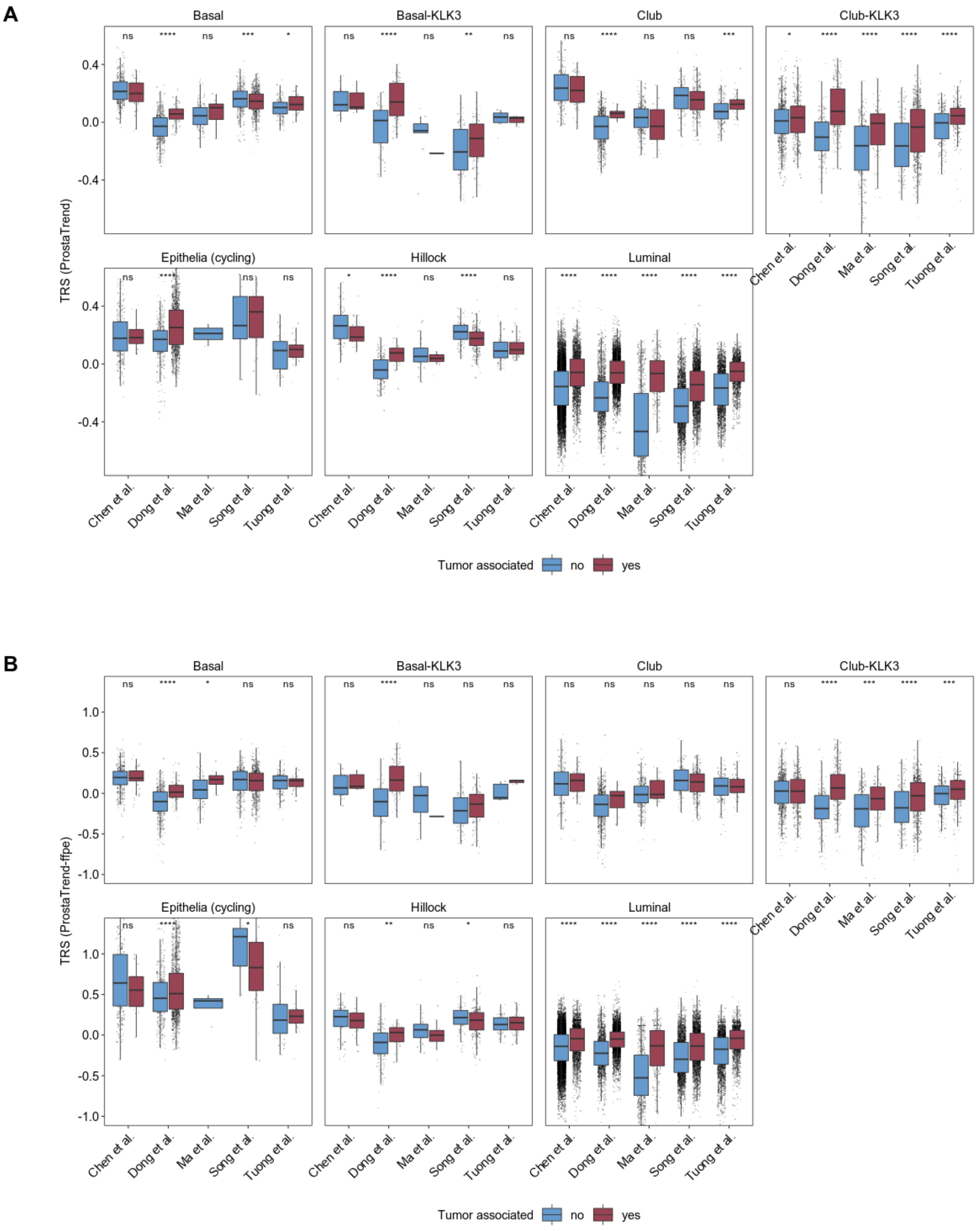

Figure S32: TRS in tumor specific and non-tumor epithelial cells. We applied a simplified TRS to each cell of the epithelial cell identity (tumor samples) using the ProstaTrend **(A)** and ProstaTrend-ffpe **(B)** signatures. Boxplots are grouped by epithelial cell types and colored by tumor specific and non-tumor cells. Classification into tumor and non-tumor cells was estimated using `inferCNV`. Differences in TRS between tumor and non-tumor cells were assessed using the Wilcoxon rank-sum test (* p <0.05, ** p <0.01, *** p <0.001, **** p <0.0001). For **(A)** and **(B)**, only data between the 0.01 and 0.99 percentile are shown.

13 Enrichment scores of gene sets for functional states of cancer

A

ProstaTrend

Gene_Set	Gene_Ratio	Bg_Ratio	p_value	p_value_adjusted
angiogenesis	8/1187	70/17158	5.20e−02	7.28e−01
apoptosis	10/1185	66/17162	5.39e−03	7.54e−02
cell_cycle	50/1145	136/17092	7.69e−25	1.08e−23 *
differentiation	16/1179	196/17032	2.02e−01	1.00e+00
dna_damage	15/1180	105/17123	2.32e−03	3.25e−02 *
dna_repair	18/1177	118/17110	4.75e−04	6.65e−03 *
emt	14/1181	88/17140	9.96e−04	1.39e−02 *
hypoxia	6/1189	81/17147	3.30e−01	1.00e+00
inflammation	5/1190	109/17119	7.76e−01	1.00e+00
invasion	17/1178	93/17135	6.07e−05	8.50e−04 *
metastasis	21/1174	162/17066	1.87e−03	2.62e−02 *
proliferation	14/1181	84/17144	6.03e−04	8.44e−03 *
quiescence	2/1193	62/17166	8.13e−01	1.00e+00
stemness	12/1183	156/17072	2.87e−01	1.00e+00

B

ProstaTrend−ffpe

Gene_Set	Gene_Ratio	Bg_Ratio	p_value	p_value_adjusted
angiogenesis	2/173	70/17158	3.44e−02	4.81e−01
apoptosis	1/174	66/17162	1.45e−01	1.00e+00
cell_cycle	22/153	136/17092	7.77e−22	1.09e−20 *
differentiation	2/173	196/17032	3.21e−01	1.00e+00
dna_damage	6/169	105/17123	9.76e−05	1.37e−03 *
dna_repair	4/171	118/17110	7.10e−03	9.95e−02
emt	5/170	88/17140	2.74e−04	3.84e−03 *
hypoxia	1/174	81/17147	1.99e−01	1.00e+00
inflammation	1/174	109/17119	3.04e−01	1.00e+00
invasion	1/174	93/17135	2.44e−01	1.00e+00
metastasis	4/171	162/17066	2.49e−02	3.49e−01
proliferation	6/169	84/17144	2.32e−05	3.25e−04 *
quiescence	0/175	62/17166	4.70e−01	1.00e+00
stemness	2/173	156/17072	2.12e−01	1.00e+00

Table S10: Gene set enrichment analysis. We performed a hypergeometric test to determine whether the ProstaTrend-ffpe signatures were enriched in gene sets for 14 functional cancer states [46]. Adjusted p-values for multiple testing were calculated using the Bonferroni method. Gene sets with an adjusted p-value <0.05 have an asterisk as suffix. Gene Ratio: Number of ProstaTrend genes present in the gene set. Bg Ratio: Number of genes from the gene sets present among all analyzed genes. In addition, we calculated TRS for each cell from the tumor samples for each of the 14 functional states (see Figure S33).

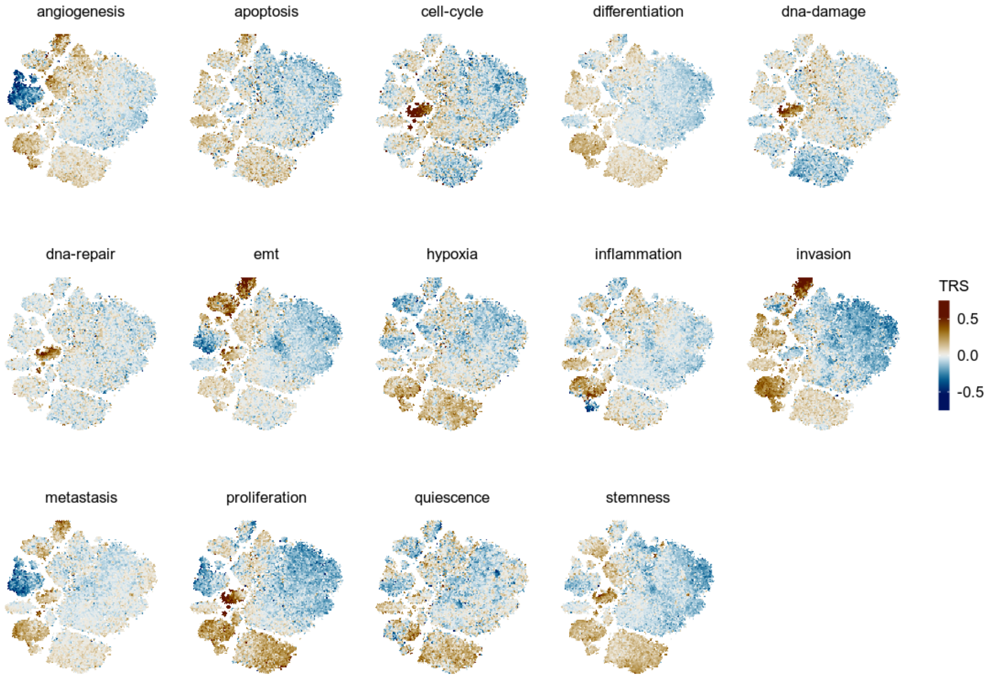

Figure S33: TRS for each cell using gene sets for functional states of cancer. We applied a simplified TRS to each cell from the tumor samples using gene sets for 14 functional cancer states [46]. To weight the gene sets, we used the estimated log hazard ratios from the ProstaTrend meta-analysis of the training cohorts [7]. Each area containing cells on the tSNE was divided into hexagonal bins, and cells within each bin were averaged. The bins are colored according to the risk scores.

Part III. Spatial transcriptomics data analysis

13.1 Enrichment scores of gene sets for cell types

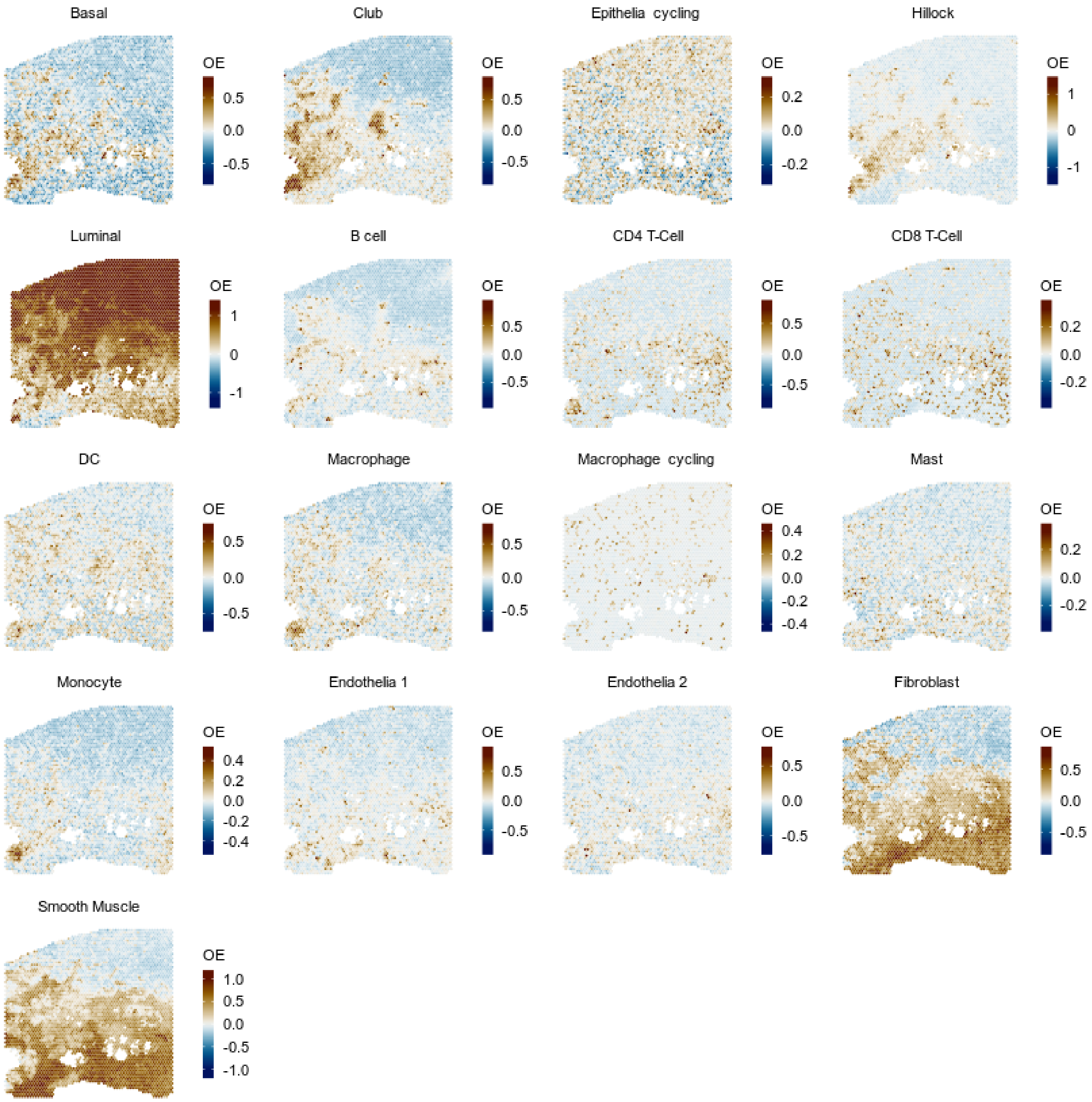

Figure S34: Enrichment of cell types in spatial transcriptomic data from a human prostate biopsy.. Each spot was scored for standardized overall enrichment of cell type markers (see chaper 9) using the function `AddModuleScore` in `Seurat`. Only gene sets whose enrichment score is at least 0.3 are shown.

13.2 Evaluation of spots associated with invasive cancer

13.2.1 Integration and clustering

We re-analyzed human normal prostate and human prostate stage III adenocarcinoma (Gleason score = 3 + 4) biopsies (Figure S35A and B). The biopsies were FFPE preserved and processed using the Visium spatial gene expression for FFPE workflow. The datasets are publicly available at 10x Genomics (https://www.10xgenomics.com/resources/datasets). Integration and clustering analysis were performed using the `Seurat` v4.1.0 `R` package [32]. For normal and tumor tissue, the 2000 most variable gene were each estimated by the "vst" method of the `FindVariableFeatures` function. We then used the `SelectIntegrationFeatures` function to select 2000 genes that were found to be highly variable across the two samples. The merged datasets were standardized with `ScaleData` and principal component analysis was performed on the standardized expression data of the variable genes using the function `RunPCA`. We used 15 principal components to integrate the two samples using the Harmony [33] method with the `Seurat` wrapper function `RunHarmony` with additional parameters (`dim.use = 1:15, group.by.vars = c("Sample")`). Using the Harmony-corrected spot embeddings, we performed clustering by computing a shared nearest neighbors (SNN) graph, as implemented in `FindNeighbors(reduction = "harmony", dims = 1:15)`. The SNN graph was used for identification of clusters using the `FindCluster` function (Louvain algorithm) with a resolution parameter set to 0.2.

13.2.2 Carcinoma associated cluster

The Harmony-corrected spot embeddings for the two samples were projected into a two-dimensional space using the tSNE method (Figure S35C). We also colored the spots according to their cluster assignment and observed that cluster 1 and 2 were exclusively present in the tumor sample (Figure S35D-F). These 2 clusters showed a high degree of conformity with the regions annotated as carcinoma in Figure S35B.

13.2.3 Spots associated with luminal cells

Since primary prostate cancer has a luminal phenotype, we next wanted to evaluate whether clusters 1 and 2 are enriched with tumor-associated luminal markers. First, in Section 8, we developed cell type markers using the PCa cell atlas. Here, we used the luminal marker gene set to evaluate whether the spots of the tumor sample contained luminal cells. Each spot was scored for overall enrichment (OE) of the luminal gene set using the function `AddModuleScore` implemented in `Seurat`.Spots with a score of >0.75 were annotated as spots containing luminal cells (Fig S35G). Using a hypergeometric test, we observed that of clusters 1, 2 and 3 were significantly enriched (adjusted p-value <0.05, Bonferroni correction) with spots containing luminal cells (Fig S35H). Second, in Section 8.2 we performed DGEA between T-luminal (Tumor associated luminal) and luminal cells from the PCa cell atlas. Here, we used the DE genes to analyze the OE in clusters associated with

carcinoma regions (cluster 1 and 2) and normal gland (cluster 3) according to the pathological annotation. The OE for T-luminal DE genes with LFC >0 is depicted in Figure S35I, LFC <0 in S35J. Figure S35I indicates that clusters 1+2 were enriched with T-luminal DE genes with LFC >0, whereas cluster 2 is enriched with T-luminal DE genes with LFC <0 (Figure S35J). We evaluated differences in OE between spots in cluster 1+2 and cluster 3 using the Wilcoxon rank-sum test. Significant differences (p-value <0.05) were observed for both gene sets.

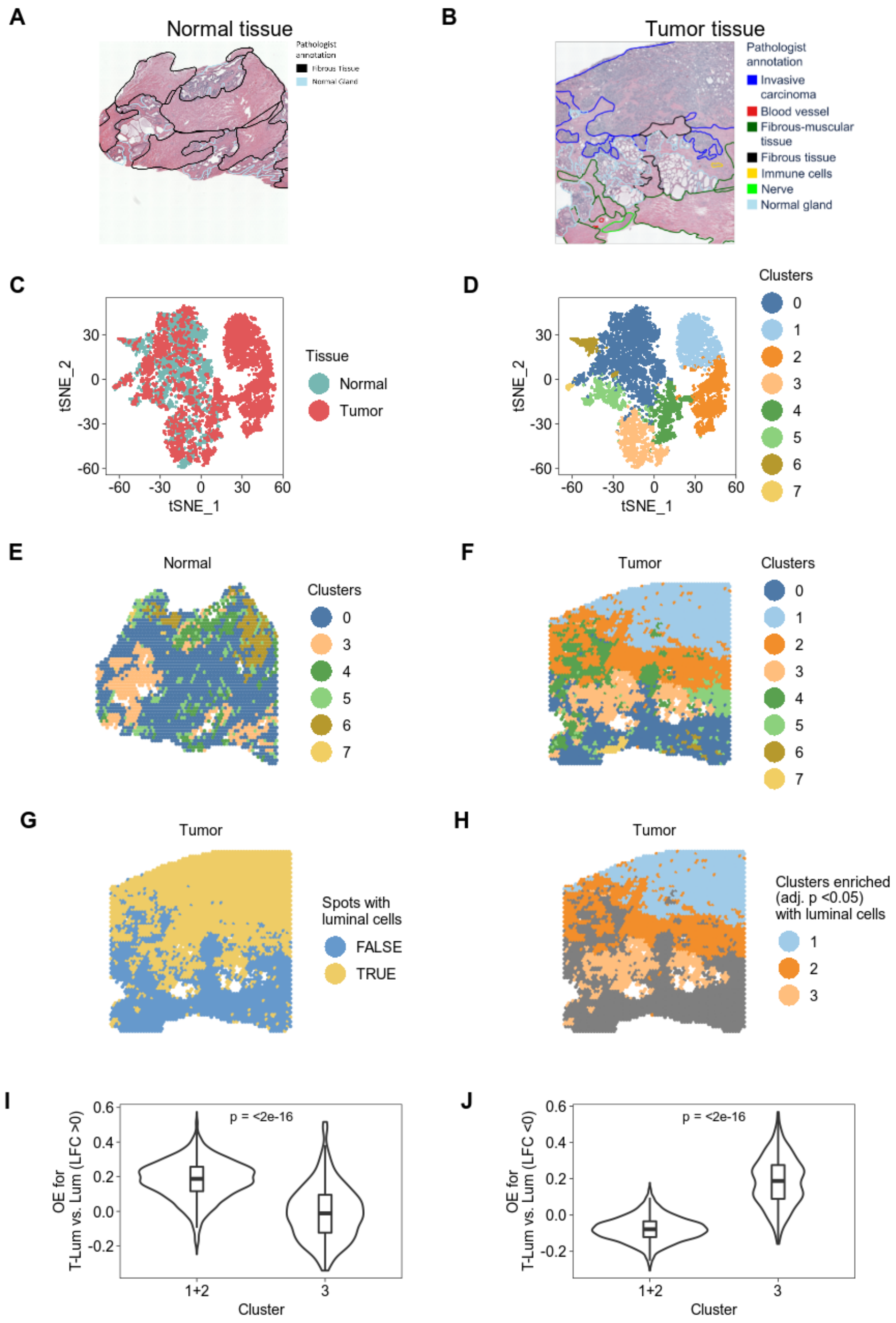

Figure S35: Integration analysis of spatial transcriptome data from human normal and cancerous prostate biopsies. see section 13.2 for methods. **(A and B)** Human normal prostate and human PCa biopsies, which was FFPE preserved and processed using the Visium spatial gene expression for FFPE workflow. Pathological annotations for the tissue were done at an overview level and were performed by Agoko NV, Belgium. **(C)** To correct for the batch effect, we integrated the spatial data for tumor and normal tissues using the Harmony method (see section 13.2). The Harmony-corrected principal components were then used to create a uniform tSNE embedding space. Dots (spot expression) are colored by tissue. **(D)** We performed graph-based clustering and colored the spots according to the identified clusters. **(E and F)** Spots for normal and cancerous tissue are colored according to assigned clusters. **(G)** Spots with and overall enrichment (OE) score >0.75 for luminal marker. **(H)** Significantly enriched clusters with spots where luminal cell were present. For this, a hypergeometric test was performed. Grey colored clusters have a p-value >0.05. **(i)** OE scores for T-luminal DE genes (LFC >0 and <0). Differences between clusters 1+2 and 3 were evaluated using the Wilcoxon rank-sum test.

13 References

1. Cancer Genome Atlas Research Network. “The Molecular Taxonomy of Primary Prostate Cancer”. en. *Cell* 163:4, 2015, pp. 1011–1025.
2. C. Kämpf, M. Specht, A. Scholz, S.-H. Puppel, G. Doose, K. Reiche, J. Schor, and J. Hackermüller. “uap: reproducible and robust HTS data analysis”. *BMC Bioinformatics* 20, 2019.
3. *bcl2fastq*. https://emea.support.illumina.com/sequencing/sequencing_software/bcl2fastq-conversion-software.html.
4. M. Schubert, S. Lindgreen, and L. Orlando. “AdapterRemoval v2: rapid adapter trimming, identification, and read merging”. *BMC Res. Notes* 9:1, 2016, p. 88.
5. D. Kim, J. M. Paggi, C. Park, C. Bennett, and S. L. Salzberg. “Graph-based genome alignment and genotyping with HISAT2 and HISAT-genotype”. *Nat. Biotechnol.* 37:8, 2019, pp. 907–915.
6. S. Anders, P. T. Pyl, and W. Huber. “HTSeq—a Python framework to work with high-throughput sequencing data”. *Bioinformatics* 31:2, 2015, pp. 166–169.
7. M. Kreuz, D. J. Otto, S. Fuessel, C. Blumert, C. Bertram, S. Bartsch, D. Loeffler, S.-H. Puppel, M. Rade, T. Buschmann, S. Christ, K. Erdmann, M. Friedrich, M. Froehner, M. H. Muders, S. Schreiber, M. Specht, M. I. Toma, F. Benigni, M. Freschi, G. Gandaglia, A. Briganti, G. B. Baretton, M. Loeffler, J. Hackermüller, K. Reiche, M. Wirth, and F. Horn. “ProstaTrend-A Multivariable Prognostic RNA Expression Score for Aggressive Prostate Cancer”. en. *Eur. Urol.* 78:3, 2020, pp. 452–459.
8. R. M. Kuhn, D. Haussler, and W. J. Kent. “The UCSC genome browser and associated tools”. en. *Brief. Bioinform.* 14:2, 2013, pp. 144–161.
9. *Andrews S: FastQC. 2010*. https://www.bioinformatics.babraham.ac.uk/projects/fastqc/.
10. T. Daley and A. D. Smith. “Predicting the molecular complexity of sequencing libraries”. *Nat. Methods* 10:4, 2013, pp. 325–327.
11. L. Wang, S. Wang, and W. Li. “RSeQC: quality control of RNA-seq experiments”. *Bioinformatics* 28:16, 2012, pp. 2184–2185.
12. S. W. Wingett and S. Andrews. “FastQ Screen: A tool for multi-genome mapping and quality control”. *F1000Res.* 7, 2018, p. 1338.
13. B. Langmead and S. L. Salzberg. “Fast gapped-read alignment with Bowtie 2”. *Nat. Methods* 9:4, 2012, pp. 357–359.
14. *fastq-tools*. https://homes.cs.washington.edu/~dcjones/fastq-tools/.
15. K. Okonechnikov, A. Conesa, and F. García-Alcalde. “Qualimap 2: advanced multi-sample quality control for high-throughput sequencing data”. en. *Bioinformatics* 32:2, 2016, pp. 292–294.

16. P. Ewels, M. Magnusson, S. Lundin, and M. Käller. “MultiQC: summarize analysis results for multiple tools and samples in a single report”. *Bioinformatics* 32:19, 2016, pp. 3047–3048.

17. M. I. Love, W. Huber, and S. Anders. “Moderated estimation of fold change and dispersion for RNA-seq data with DESeq2”. *Genome Biol.* 15:12, 2014, p. 550.

18. R. Edgar, M. Domrachev, and A. E. Lash. “Gene Expression Omnibus: NCBI gene expression and hybridization array data repository”. eng. *Nucleic Acids Res.* 30:1, 2002, pp. 207–210.

19. S. Davis and P. S. Meltzer. “GEOquery: a bridge between the Gene Expression Omnibus (GEO) and BioConductor”. en. *Bioinformatics* 23:14, 2007, pp. 1846–1847.

20. B. S. Carvalho and R. A. Irizarry. “A framework for oligonucleotide microarray preprocessing”. en. *Bioinformatics* 26:19, 2010, pp. 2363–2367.

21. M. Dai, P. Wang, A. D. Boyd, G. Kostov, B. Athey, E. G. Jones, W. E. Bunney, R. M. Myers, T. P. Speed, H. Akil, S. J. Watson, and F. Meng. “Evolving gene/transcript definitions significantly alter the interpretation of GeneChip data”. en. *Nucleic Acids Res.* 33:20, 2005, e175.

22. M. Fraser, V. Y. Sabelnykova, T. N. Yamaguchi, L. E. Heisler, J. Livingstone, V. Huang, Y.-J. Shiah, F. Yousif, X. Lin, A. P. Masella, N. S. Fox, M. Xie, S. D. Prokopec, A. Berlin, E. Lalonde, M. Ahmed, D. Trudel, X. Luo, T. A. Beck, A. Meng, J. Zhang, A. D’Costa, R. E. Denroche, H. Kong, S. M. G. Espiritu, M. L. K. Chua, A. Wong, T. Chong, M. Sam, J. Johns, L. Timms, N. B. Buchner, M. Orain, V. Picard, H. Hovington, A. Murison, K. Kron, N. J. Harding, C. P’ng, K. E. Houlahan, K. C. Chu, B. Lo, F. Nguyen, C. H. Li, R. X. Sun, R. de Borja, C. I. Cooper, J. F. Hopkins, S. K. Govind, C. Fung, D. Waggott, J. Green, S. Haider, M. A. Chan-Seng-Yue, E. Jung, Z. Wang, A. Bergeron, A. Dal Pra, L. Lacombe, C. C. Collins, C. Sahinalp, M. Lupien, N. E. Fleshner, H. H. He, Y. Fradet, B. Tetu, T. van der Kwast, J. D. McPherson, R. G. Bristow, and P. C. Boutros. “Genomic hallmarks of localized, non-indolent prostate cancer”. en. *Nature* 541:7637, 2017, pp. 359–364.

23. J. T. Leek, W. E. Johnson, H. S. Parker, A. E. Jaffe, and J. D. Storey. “The sva package for removing batch effects and other unwanted variation in high-throughput experiments”. en. *Bioinformatics* 28:6, 2012, pp. 882–883.

24. J. Gao, B. A. Aksoy, U. Dogrusoz, G. Dresdner, B. Gross, S. O. Sumer, Y. Sun, A. Jacobsen, R. Sinha, E. Larsson, E. Cerami, C. Sander, and N. Schultz. “Integrative analysis of complex cancer genomics and clinical profiles using the cBioPortal”. en. *Sci. Signal.* 6:269, 2013, p. l1.

25. J. Li, C. Xu, H. J. Lee, S. Ren, X. Zi, Z. Zhang, H. Wang, Y. Yu, C. Yang, X. Gao, J. Hou, L. Wang, B. Yang, Q. Yang, H. Ye, T. Zhou, X. Lu, Y. Wang, M. Qu, Q. Yang, W. Zhang, N. M. Shah, E. C. Pehrsson, S. Wang, Z. Wang, J. Jiang, Y. Zhu, R. Chen, H. Chen, F. Zhu, B. Lian, X. Li, Y. Zhang, C. Wang, Y. Wang, G. Xiao, J. Jiang, Y. Yang, C. Liang, J. Hou, C. Han, M. Chen, N. Jiang, D. Zhang, S. Wu, J. Yang, T. Wang, Y. Chen, J. Cai, W. Yang, J. Xu, S. Wang, X. Gao, T. Wang, and Y. Sun. “A genomic and epigenomic atlas of prostate cancer in Asian populations”. en. *Nature* 580:7801, 2020, pp. 93–99.

26. R. Li, J. Zhu, W. Zhong, and Z. Jia. "Comprehensive evaluation of machine learning models and gene expression signatures for prostate cancer prognosis using large population cohorts". en. *Cancer Res.*, 2022.

27. B. Dong, J. Miao, Y. Wang, W. Luo, Z. Ji, H. Lai, M. Zhang, X. Cheng, J. Wang, Y. Fang, H. H. Zhu, C. W. Chua, L. Fan, Y. Zhu, J. Pan, J. Wang, W. Xue, and W.-Q. Gao. "Single-cell analysis supports a luminal-neuroendocrine transdifferentiation in human prostate cancer". en. *Commun Biol* 3:1, 2020, p. 778.

28. H. Song, H. N. W. Weinstein, P. Allegakoen, M. H. Wadsworth, J. Xie, H. Yang, E. A. Castro, K. L. Lu, B. A. Stohr, F. Y. Feng, P. R. Carroll, B. Wang, M. R. Cooperberg, A. K. Shalek, and F. W. Huang. "Single-cell analysis of human primary prostate cancer reveals the heterogeneity of tumor-associated epithelial cell states". en. *Nat. Commun.* 13:1, 2022, pp. 1–20.

29. S. Chen, G. Zhu, Y. Yang, F. Wang, Y.-T. Xiao, N. Zhang, X. Bian, Y. Zhu, Y. Yu, F. Liu, K. Dong, J. Mariscal, Y. Liu, F. Soares, H. Loo Yau, B. Zhang, W. Chen, C. Wang, D. Chen, Q. Guo, Z. Yi, M. Liu, M. Fraser, D. D. De Carvalho, P. C. Boutros, D. Di Vizio, Z. Jiang, T. van der Kwast, A. Berlin, S. Wu, J. Wang, H. H. He, and S. Ren. "Single-cell analysis reveals transcriptomic remodellings in distinct cell types that contribute to human prostate cancer progression". en. *Nat. Cell Biol.* 23:1, 2021, pp. 87–98.

30. X. Ma, J. Guo, K. Liu, L. Chen, D. Liu, S. Dong, J. Xia, Q. Long, Y. Yue, P. Zhao, F. Hu, Z. Xiao, X. Pan, K. Xiao, Z. Cheng, Z. Ke, Z.-S. Chen, and C. Zou. "Identification of a distinct luminal subgroup diagnosing and stratifying early stage prostate cancer by tissue-based single-cell RNA sequencing". en. *Mol. Cancer* 19:1, 2020, p. 147.

31. Z. K. Tuong, K. W. Loudon, B. Berry, N. Richoz, J. Jones, X. Tan, Q. Nguyen, A. George, S. Hori, S. Field, A. G. Lynch, K. Kania, P. Coupland, A. Babbage, R. Grenfell, T. Barrett, A. Y. Warren, V. Gnanapragasam, C. Massie, and M. R. Clatworthy. "Resolving the immune landscape of human prostate at a single-cell level in health and cancer". en. *Cell Rep.* 37:12, 2021, p. 110132.

32. Y. Hao, S. Hao, E. Andersen-Nissen, W. M. Mauck 3rd, S. Zheng, A. Butler, M. J. Lee, A. J. Wilk, C. Darby, M. Zager, P. Hoffman, M. Stoeckius, E. Papalexi, E. P. Mimitou, J. Jain, A. Srivastava, T. Stuart, L. M. Fleming, B. Yeung, A. J. Rogers, J. M. McElrath, C. A. Blish, R. Gottardo, P. Smibert, and R. Satija. "Integrated analysis of multimodal single-cell data". en. *Cell* 184:13, 2021, 3573–3587.e29.

33. I. Korsunsky, N. Millard, J. Fan, K. Slowikowski, F. Zhang, K. Wei, Y. Baglaenko, M. Brenner, P.-R. Loh, and S. Raychaudhuri. "Fast, sensitive and accurate integration of single-cell data with Harmony". en. *Nat. Methods* 16:12, 2019, pp. 1289–1296.

34. R. Fu, A. E. Gillen, R. M. Sheridan, C. Tian, M. Daya, Y. Hao, J. R. Hesselberth, and K. A. Riemondy. "clustifyr: an R package for automated single-cell RNA sequencing cluster classification". en. *F1000Res.* 9, 2020, p. 223.

35. G. H. Henry, A. Malewska, D. B. Joseph, V. S. Malladi, J. Lee, J. Torrealba, R. J. Mauck, J. C. Gahan, G. V. Raj, C. G. Roehrborn, G. C. Hon, M. P. MacConmara, J. C. Reese, R. C. Hutchinson, C. M. Vezina, and D. W. Strand. “A Cellular Anatomy of the Normal Adult Human Prostate and Prostatic Urethra”. en. *Cell Rep.* 25:12, 2018, 3530–3542.e5.

36. J. Racle, K. de Jonge, P. Baumgaertner, D. E. Speiser, and D. Gfeller. “Simultaneous enumeration of cancer and immune cell types from bulk tumor gene expression data”. en. *Elife* 6, 2017.

37. A.-C. Villani, R. Satija, G. Reynolds, S. Sarkizova, K. Shekhar, J. Fletcher, M. Griesbeck, A. Butler, S. Zheng, S. Lazo, L. Jardine, D. Dixon, E. Stephenson, E. Nilsson, I. Grundberg, D. McDonald, A. Filby, W. Li, P. L. De Jager, O. Rozenblatt-Rosen, A. A. Lane, M. Haniffa, A. Regev, and N. Hacohen. “Single-cell RNA-seq reveals new types of human blood dendritic cells, monocytes, and progenitors”. *Science* 356:6335, 2017, eaah4573–eaah4573.

38. J. Jiang, A. Faiz, M. Berg, O. A. Carpaij, C. J. Vermeulen, S. Brouwer, L. Hesse, S. A. Teichmann, N. H. T. Ten Hacken, W. Timens, M. van den Berge, and M. C. Nawijn. “Gene signatures from scRNA-seq accurately quantify mast cells in biopsies in asthma”. en. *Clin. Exp. Allergy* 50:12, 2020, pp. 1428–1431.

39. T. S. Adams, J. C. Schupp, S. Poli, E. A. Ayaub, N. Neumark, F. Ahangari, S. G. Chu, B. A. Raby, G. DeIuliis, M. Januszyk, Q. Duan, H. A. Arnett, A. Siddiqui, G. R. Washko, R. Homer, X. Yan, I. O. Rosas, and N. Kaminski. “Single-cell RNA-seq reveals ectopic and aberrant lung-resident cell populations in idiopathic pulmonary fibrosis”. en. *Sci Adv* 6:28, 2020, eaba1983.

40. E. Z. Macosko, A. Basu, R. Satija, J. Nemesh, K. Shekhar, M. Goldman, I. Tirosh, A. R. Bialas, N. Kamitaki, E. M. Martersteck, J. J. Trombetta, D. A. Weitz, J. R. Sanes, A. K. Shalek, A. Regev, and S. A. McCarroll. “Highly Parallel Genome-wide Expression Profiling of Individual Cells Using Nanoliter Droplets”. en. *Cell* 161:5, 2015, pp. 1202–1214.

41. E. Azizi, A. J. Carr, G. Plitas, A. E. Cornish, C. Konopacki, S. Prabhakaran, J. Nainys, K. Wu, V. Kiseliovas, M. Setty, K. Choi, R. M. Fromme, P. Dao, P. T. McKenney, R. C. Wasti, K. Kadaveru, L. Mazutis, A. Y. Rudensky, and D. Pe’er. “Single-Cell Map of Diverse Immune Phenotypes in the Breast Tumor Microenvironment”. en. *Cell* 174:5, 2018, 1293–1308.e36.

42. S. C. van den Brink, F. Sage, Á. Vértesy, B. Spanjaard, J. Peterson-Maduro, C. S. Baron, C. Robin, and A. van Oudenaarden. “Single-cell sequencing reveals dissociation-induced gene expression in tissue subpopulations”. en. *Nat. Methods* 14:10, 2017, pp. 935–936.

43. L. Machado, F. Relaix, and P. Mourikis. “Stress relief: emerging methods to mitigate dissociation-induced artefacts”. en. *Trends Cell Biol.* 31:11, 2021, pp. 888–897.

44. I. Tirosh, B. Izar, S. M. Prakadan, M. H. Wadsworth 2nd, D. Treacy, J. J. Trombetta, A. Rotem, C. Rodman, C. Lian, G. Murphy, M. Fallahi-Sichani, K. Dutton-Regester, J.-R. Lin, O. Cohen, P. Shah, D. Lu, A. S. Genshaft, T. K. Hughes, C. G. K. Ziegler, S. W. Kazer, A. Gaillard, K. E. Kolb, A.-C. Villani, C. M. Johannessen, A. Y. Andreev, E. M. Van Allen, M. Bertagnolli, P. K. Sorger, R. J.

Sullivan, K. T. Flaherty, D. T. Frederick, J. Jané-Valbuena, C. H. Yoon, O. Rozenblatt-Rosen, A. K. Shalek, A. Regev, and L. A. Garraway. “Dissecting the multicellular ecosystem of metastatic melanoma by single-cell RNA-seq”. en. *Science* 352:6282, 2016, pp. 189–196.

45. A. L. Haber, M. Biton, N. Rogel, R. H. Herbst, K. Shekhar, C. Smillie, G. Burgin, T. M. Delorey, M. R. Howitt, Y. Katz, I. Tirosh, S. Beyaz, D. Dionne, M. Zhang, R. Raychowdhury, W. S. Garrett, O. Rozenblatt-Rosen, H. N. Shi, O. Yilmaz, R. J. Xavier, and A. Regev. “A single-cell survey of the small intestinal epithelium”. en. *Nature* 551:7680, 2017, pp. 333–339.

46. H. Yuan, M. Yan, G. Zhang, W. Liu, C. Deng, G. Liao, L. Xu, T. Luo, H. Yan, Z. Long, A. Shi, T. Zhao, Y. Xiao, and X. Li. “CancerSEA: a cancer single-cell state atlas”. en. *Nucleic Acids Res.* 47:D1, 2019, pp. D900–D908.
